# Blockade of TGF-β signalling alleviates human adipose stem cell senescence induced by native ECM in obesity visceral white adipose tissue

**DOI:** 10.1186/s13287-023-03525-y

**Published:** 2023-10-08

**Authors:** Xueya Han, Weihong Li, Xu He, Xin Lu, Yu Zhang, Yaqiong Li, Guoyun Bi, Xuqing Ma, Xiaowu Huang, Rixing Bai, Haiyan Zhang

**Affiliations:** 1 Department of Cell Biology, School of Basic Medical Science, Capital Medical University, Beijing, 100069 China; 2 Experimental Center for Basic Medical Teaching, School of Basic Medical Science, Capital Medical University, Beijing, 100069 China; 3 Fu Xing Hospital, Capital Medical University, Beijing, 100038 China; 4 Department of General Surgery, Beijing Tian Tan Hospital, Capital Medical University, Beijing, 100070 China

**Keywords:** Human adipose stromal/stem cell, Obesity, visceral white adipose tissue, Cell-derived extracellular matrix, Mitochondria, TGF-β1, Ageing, Neutralizing antibody

## Abstract

**Background:**

Abdominal obesity is appreciated as a major player in insulin resistance and metabolically dysfunctional adipose tissue. Inappropriate extracellular matrix (ECM) remodelling and functional alterations in human adipose stromal/stem cells (hASCs) have been linked with visceral white adipose tissue (vWAT) dysfunction in obesity. Understanding the interactions between hASCs and the native ECM environment in obese vWAT is required for the development of future therapeutic approaches for obesity-associated metabolic complications.

**Methods:**

The phenotypes and transcriptome properties of hASCs from the vWAT of obese patients and lean donors were assessed. The hASC-derived matrix from vWAT of obese or lean patients was generated in vitro using a decellularized method. The topography and the major components of the hASC-derived matrix were determined. The effects of the obese hASC-derived matrix on cell senescence and mitochondrial function were further determined.

**Results:**

We showed that hASCs derived from the vWAT of obese patients exhibited senescence and were accompanied by the increased production of ECM. The matrix secreted by obese hASCs formed a fibrillar suprastructure with an abundance of fibronectin, type I collagen, and transforming growth factor beta 1 (TGF-β1), which resembles the native matrix microenvironment of hASCs in vWAT derived from obese patients. Furthermore, the obese hASC-derived matrix promoted lean hASC ageing and induced mitochondrial dysfunction compared to the lean hASC-derived matrix. Blockade of TGF-β1 signalling using an anti-TGF-β1 neutralizing antibody alleviated the lean hASC senescence and mitochondrial dysfunction induced by the obese hASC-derived matrix.

**Conclusions:**

Native ECM in obesity vWAT initiates hASC senescence through TGF-β1-mediated mitochondrial dysfunction. These data provide a key mechanism for understanding the importance of cell-ECM interactions in hASCs senescence in obesity.

**Supplementary Information:**

The online version contains supplementary material available at 10.1186/s13287-023-03525-y.

## Background

The constantly increasing prevalence of obesity poses a global challenge [1–3]. Obesity, especially abdominal obesity, is associated with an increased risk of insulin resistance, type 2 diabetes mellitus, cardiovascular diseases, fatty liver disease, coronary heart disease, stroke, osteoarthritis, and several cancers [1, 4–7]. With chronic obesity, visceral white adipose tissue (vWAT) expansion, unresolved inflammation, inappropriate extracellular matrix (ECM) remodelling and insufficient angiogenic potential contribute to the pathogenesis of dysfunctional adipose tissue [8–11].

The major adaptive processes in adipose tissue, including expansion and maintenance of adipocyte number, all involve de novo adipogenesis and therefore rely on the proper functioning of human adipose stromal/stem cells (hASCs), an important resident stem cell population in the stromal vascular fraction (SVF) [12–15]. Recent single-cell transcriptomic studies have further refined this concept, and identifying hASCs on the basis of unique gene-expression signatures [16, 17].

Studies have shown that a reduced number of pre-adipocytes are able to differentiate into adipocytes in abdominal subcutaneous WAT of obese patients and this correlated with body mass index (BMI) [18]. Recent evidence indicates that hASCs isolated from obese patients show changes in their properties and lose their beneficial functions, including multipotent state, metabolism, and immunomodulation [19–23]. The alteration of stem cells properties occurs in response to many different triggers, including cell-intrinsic damage and cell-extrinsic changes in stem cell niches [24, 25]. Whether the obesity-associated local microenvironment activates the functional alteration of hASCs remains to be elucidated.

As a critical structural support and dynamic microenvironment, the ECM provides a scaffold in which insoluble ECM proteins can integrate complex, multivalent exogenous bioactive factors, and biomechanical cues into tissue-specific stem cells in a spatially patterned and regulated fashion [26–28]. On the other hand, the molecular composition, structural features, and nanomechanical properties of native ECM are determined by the stem cell itself [29–31]. Stem cells produce the matrix via posttranslational modification, fibrillogenesis, aligned deposition, and remodelling of matrix molecules under a physical or pathological state [30, 32–34].

To address the exact role of the native-like multicomponent ECM of tissue-resident stem cells in vitro*,* a methodology enabling the acquisition of bone marrow mesenchymal stem cell (BMSC)- and hASC-secreted matrix has been developed [30, 35]. BMSC-derived matrices have emerged as in vitro cell culture substrates to better recapitulate the native stem cell microenvironment outside the body and the interaction between the native niche and tissue-resident stem cells under a physical state [30, 31, 35, 36]. Native BMSC- and hASC-derived matrices are different in their ability to control MSC behaviour. Culture on BMSC-derived matrices significantly increased BMSC-responsiveness to rhBMP-2 (an osteogenic inducer) [37] and enhanced osteoregeneration [38, 39], while culture on hASC-derived matrices enhanced responsiveness to rosiglitazone (an adipogenic inducer) [37]. While many structural proteins (e.g., collagen and fibronectin) were found at equivalent levels in both BMSC- and hASC-derived matrices, the architecture (e.g., fibre orientation; surface roughness) and physical properties (storage modulus and surface energy) of each were unique [40, 41]. Differences in characteristics were also found in ASC matrices derived from obese mice and lean control mice. The ASC matrices derived from obese mice deposited denser and stiffer ECMs relative to ASCs from lean control mice [42, 43].

Several studies have shown that hASCs from obese patients have a declined proliferative ability, telomerase activity and DNA telomere length, a loss of osteogenic and adipogenic potential, mitochondrial dynamics deterioration and a reduction in typical immunosuppressive activities [21–23, 44–49]. However, little is known about the alterations in the properties of the hASC-derived matrix derived from obese patients or its impact on itself.

In the present study, we found that the obese hASC-derived matrix has abundant fibronectin, type I collagen, and transforming growth factor beta 1 (TGF-β1), which resembles the native matrix microenvironment of hASCs in vWAT derived from obese patients. Furthermore, the obese hASC-derived matrix increased the proportion of aged cells and induced mitochondrial dysfunction in hASCs derived from the lean donor. Blockade of TGF-β1 signalling using an anti-TGF-β1 neutralizing antibody alleviated the lean hASC senescence and mitochondrial dysfunction induced by the obese hASC-derived matrix.

## Methods

### Human adipose tissues

All human vWAT samples from obese patients (BMI > 30 kg/m^2^) and age-matched normal weight donors (BMI < 24 kg/m^2^) were obtained with informed patient consent and under the approval of of IRB of Beijing Tiantan Hospital, Capital Medical University (Beijing, China) as previously described [50]. Age and gender of human patients are provided in Table 1. All experimental protocols were approved and carried out in accordance with the relevant guidelines and regulations of the Ethics Committee of Capital Medical University, China. All investigations were conducted according to the principles expressed in the Declaration of Helsinki.

**Table 1:** Characteristics of the patients

	Lean (*n* = 3)	Obese (*n* = 8)	*p* Value
Body mass index (kg/m^2^)	20.93 ± 0.04	45.35 ± 1.11	0.0020
Age (years)	26.67 ± 1.91	31.13 ± 0.82	0.3717
Gender (male/female)	3 (0/3)	8 (4/4)	–
Glucose (mmol/L)	4.76 ± 0.11	7.92 ± 0.42	0.1748
Fasting serum insulin (uU/mL)	–	20.62 ± 0.98	–
HOMA-IR	–	2.95 ± 0.16	–
Triglyceride (mmol/L)	0.85 ± 0.13	2.67 ± 0.23	0.1623
Total cholesterol (mmol/L)	4.78 ± 0.26	4.49 ± 0.06	0.5225
HDL-cholesterol (mmol/L)	1.44 ± 0.01	1.01 ± 0.02	0.0047
LDL-cholesterol (mmol/L)	2.69 ± 0.22	2.79 ± 0.08	0.8418

HOMA-IR, homeostasis model assessment of insulin resistance; HDL, high-density lipoprotein; LDL, low-density lipoprotein. Data are expressed as the means ± SEM

### Cell culture

hASC from three different lean donors and eight obese donors was developed individually as our previous protocols [13]. Briefly, the adipose tissue was minced into pieces, washed twice in phosphate-buffered saline (PBS, pH 7.2) with 5% penicillin–streptomycin (HyClone, Logan, UT, USA), and then digested with 0.075% type I collagenase (Invitrogen, Grand Island, NY) for 180 min at 37 °C with gentle shaking. The digests were then centrifuged at 1,500 rpm for 5 min at 4 °C. The pellets were washed twice with PBS, resuspended in DMEM/F12 (Invitrogen, Grand Island, NY, USA) supplemented with 10% foetal bovine serum (FBS, Invitrogen), 100 U/ml penicillin and 100 µg/ml streptomycin (HyClone) at 37 °C with 5% CO_2_. Once reaching at 80–90% confluence, the cells were passaged using 0.05% trypsin-0.02% EDTA solution and plated at a density of 5000 cells/cm^2^. Cells from passages four to seven were used in this study.

### Preparation and assessment of decellularized hASC-derived matrix

Prior to cell seeding, the glass coverslips (NEST Biotechnology, Wuxi, China) were prepared as our previous protocols [51]. Briefly, the coverslips were placed into 24-well cell culture plates (Sigma-Aldrich, St. Louis, MO, USA), were coated with 0.2% gelatin solution for 1 h at 37 °C, and washed with in PBS, then cross-linking of gelatin with 1% glutaraldehyde for 30 min at room temperature. The coverslips were washed with PBS, then treated for 30 min with PBS containing 1 M ethanolamine at room temperature and then washed three times extensively with PBS.

To prepare the hASC-derived matrix, hASCs were seeded at a density of 20,000 cells/cm^2^ in 24-well tissue culture plates with treated glass coverslips, respectively. Cells were cultured for 7 days, with medium changes every other day. Then, the cultured cells were washed twice with PBS and incubation 50 mM EDTA for 4 h at 4 °C, decellularized by incubation with PBS containing 1% Triton X-100 and 50 mM NH_4_OH for 10 min at 37 °C [41, 52], washed twice with PBS. The obtained matrices were stored at 4 °C until usage for further experiments.

For a qualitative evaluation of the decellularization efficiency, hASC-derived matrix was counterstained with 4′,6-diamidino-2-phenylindole (DAPI, Sigma-Aldrich) after decellularization.

For blocked TGF-β1 signalling, the obese hASC-derived matrix was treated with anti-TGF-β1 neutralizing antibody (NeutraKine^®^ TGF beta 1 Monoclonal antibody, 10 μg/ml, proteintech, Wuhan, China) or control IgG1 (proteintech) for 24 h [53, 54].

### Culture of cells on hASC-derived matrix

Before cell seeding, hASC-derived matrix was incubated overnight with growth medium. Lean hASCs were seeded at a density of 27,500 cells/cm^2^ on lean hASC-derived matrix, obese hASC-derived matrix, obese hASC-derived matrix treated with anti-TGF-β1 neutralizing antibody or control IgG1 separately. Cells were cultured for 5 days, with medium changes every other day.

### Cellular growth curves analysis

hASCs were seeded at a density of 4000 cells/well that were plated in triplicate in growth medium in E-Plate 16 (ACEA Biosciences, San Diego, CA) according to the manufacturer's instructions. During cell growth, the interactions of the living cells with the biocompatible microelectrode surface changed the electrical impedance, which was monitored by the xCELLigence Real-Time Cell Analyzer-MP system (ACEA Biosciences). The cell index (CI) was read automatically and continuously recorded every 1 h for 120 h as CI ± standard deviation (SD). The software of the real-time cellular analysis (RTCA) systems calculates the slope at 20–120 h.

### Flow cytometry

For the flow cytometric detection of surface antigens, hASCs (1 × 10^6^ cells) were washed and resuspended in stain buffer (BD Biosciences, San Jose, CA) containing saturating concentrations (1:100 dilution) of the following conjugated mouse monoclonal antibodies against human antigens (BD Biosciences) on ice for 30 min in the dark: PE-CD105 (endoglin, ENG), FITC-CD90 (Thy-1 cell surface antigen, THY1), PE-CD73 (5′-nucleotidase ecto, NT5E), and PE-CD34. FITC-labelled mouse IgG1κ Isotype control and PE-labelled mouse IgG2ακ Isotype control were also included. The cell suspensions were washed twice and resuspended in FBS for flow cytometer (BD Accuri C6; BD Biosciences) using FLOWJOTM software (TreeStar, Inc., Ashland, OR). The antibodies used are listed in Additional file 1: Table S1.

### SA-β-galactosidase staining

The senescence β-galactosidase (SA-β-gal) staining was performed by a cellular senescence assay kit (Beyotime Biotechnology, Shanghai, China) following the manufacture’s instruction. Briefly, the cells were treated with SA-β-Gal fixing buffer at room temperature for 15 min. Cells were washed 3 times with PBS and stained with working solution (10 μl buffer A, 10 μl buffer B, 930 μl buffer C, and 50 μl X-Gal solution) overnight at 37 °C without CO_2_ for 12–16 h. The images were captured using an Axio Imager A2 microscope (Zeiss, Oberkochen, Germany). The analysis of the percentage of SA-β-gal-positive area was performed using the plug-in Cell Counter in the ImageJ software (National Institutes of Health). SA-β-Gal-positive area was determined as the percentage of cells staining blue (light or dark blue) with respect to the total area of cells. The population of SA-β-Gal-positive cells was determined by counting 15–20 fields in each group.

### DNA damage assay

The DNA damage was performed by a H2A histone family member X (H2AX) Phosphorylation Assay Kit (Beyotime biotechnology, Shanghai, China) following the manufacture’s instruction. Briefly, the cells were treated with fixing buffer at room temperature for 15 min. Cells then were washed 5 times with wash buffer, followed by rinsed and blocked with blocking buffer for 20 min at room temperature. The cells were then incubated with the anti-γ-H2AX primary antibodies at 4 °C overnight. Following three 5-min washes in wash buffer with gentle agitation, an Alexa Fluor 488 conjugated secondary antibody was added, and the samples were incubated for 1 h at room temperature. The nuclei were counter-stained with DAPI. The images of fluorescently labelled were captured using a Leica TCS SP5 confocal laser scanning microscope (Leica, Wetzlar, Germany). The relative density of γ-H2AX and DAPI in each cell nuclei was assessed using ImageJ software [55].

### Immunofluorescence staining

For immunofluorescence analysis, the cells were fixed with 4% paraformaldehyde for 20 min at room temperature, followed by permeabilization with 0.3% Triton X-100 in PBS for 5 min. The cells were blocked with 10% goat serum (ZSGB-BIO, Beijing, China) for 60 min at room temperature. The cells were then incubated with the primary antibodies at 4 °C overnight. Following three 5-min washes in PBS with gentle agitation, an Alexa Fluor-conjugated secondary antibody (Invitrogen) at 1:500 was added, and the samples were incubated for 1 h at 37 °C. The nuclei were counter-stained with DAPI (Sigma-Aldrich). The images of fluorescently labelled were captured using a Leica TCS SP8 STED confocal laser scanning microscope and a Leica TCS SP5 confocal laser scanning microscope (Leica, Wetzlar, Germany). Relative intensities of staining were quantitatively assessed using Image J software.

To visualize F-actin, the cells were stained with Alexa Fluor 568-conjugated phalloidin (Invitrogen) for 30 min at room temperature. The images of fluorescently labelled were captured using a Leica TCS SP8 STED confocal laser scanning microscope and a Leica TCS SP5 confocal laser scanning microscope (Leica, Wetzlar, Germany). The fluorescent co-localization between F-actin signal and vinculin was quantified by Pearson’s coefficient analysis using ImageJ ‘Colocalization Threshold’ software.

### Haematoxylin–eosin, Sirius Red Stain and immunohistochemistry

For histological preparation, parts of the adipose tissue were fixed with 10% formalin for 24 h at room temperature, followed by dehydration with graded alcohols, and embedding in paraffin. The embedded tissues were then consecutively sliced into 7-μm-thick sections, followed by rehydration and routinely stained with haematoxylin–eosin (H&E) or Sirius Red Stain (Solarbio, Beijing, China). The sections were imaged under an Axio Imager A2 microscope (Zeiss).

For immunohistochemistry, after deparaffinized and rehydrated, the sections were heated to recover antigens and were examined with the PV-8000 DAB Kit (ZSGB-BIO). Briefly, the sections were incubated in 3% H_2_O_2_ for 10 min at room temperature. Subsequently, the sections were incubated with primary antibodies, which are listed in Additional file 1: Table S1, at 4 °C overnight. The sections were then incubated with a tagged goat anti-mouse/anti-rabbit IgG secondary antibody (ZSGB-BIO) for 20 min at room temperature. The DAB colour reagent kit (ZSGB-BIO) was used for colour development, and the positive cells showed a brown colour. The sections were imaged under an Axio Imager A2 microscope (Zeiss). The percentage of positive area of Sirius Red Stain, collagen I and fibronectin in lean and obese donors was analysed using ImageJ software.

For quantifying the area of the adipocytes using ImageJ software, open the picture, use the straight line tool to draw the ruler line segment, click Analyze, and Set Scale, enter the length of the known line segment in the Known Distance Box, and enter the unit in the Unit Of Length, and click OK. Use the Freehand Selection tool to draw the adipocyte to be measured, click Analyze, and then Measure.

### Scanning electron microscopy and quantitative analysis

Scanning electron microscopy (SEM) analysis was performed as previously described [51]. The samples were then examined under a Hitachi S-4800 scanning electron microscope (Hitachi, Tokyo, Japan).

For quantitative analysis of hASC-derived matrix, the main orientation and anisotropy of fibrillar structures were measured and assessed in images using the ImageJ plug-in FibrilTool. Fibre yields and the image channel of ECM were manually selected. All orientation data were averaged, and the mean value corresponds to the main orientation of fibres in each image. Angles of the same image were normalized by mean value and marked as reorientation angle, which ensures independence of variations in the fibril direction. The anisotropy of arrays ranges from 0 when the orientation is random to a maximum theoretical value of 1 when all fibres are oriented in the same direction. The mean is based on 12 regions of interest in three different donors per group.

### Mitochondrial labelling and imaging

Cells were plated on confocal chambers (NEST) at a density of 35,000 cells per well. Next, cells were stained with Hoechst 33,342 (0.2 μg/ml, Thermo Fisher Scientific, Waltham, MA, USA) for 20 min, following incubation, 50 nM MitoTracker Green FM (M7514, Thermo Fisher Scientific) or 25 nM tetramethylrhodamine methyl ester (TMRM) (I34361, Thermo Fisher Scientific) was added, for 30 min at 37 °C, in a CO_2_ incubator, then washed three times with PBS. After washing, the images of fluorescently labelled mitochondria and nuclei were captured using Leica TCS SP8 STED confocal laser scanning microscope (Leica). The relative intensities of Mito Tracker Green FM and TMRM were calculated in each cell using ImageJ software as previously described [50].

### Mitochondrial stress test

Mitochondrial function was detected in live cells using the Agilent Seahorse XF Cell MitoStress Test Kit (Seahorse Bioscience, Billerica, MA, USA) [50]. Briefly, hASCs were seeded at a density of 3 × 10^4^ cells/well on 24-well XFe24 cell culture microplates for 5 days. Before the measurement, the cells were washed twice with warmed DMEM assay medium (XF assay modified DMEM supplemented with 10 mM XF glucose, 1 mM XF pyruvate, 2 mM XF glutamine, pH 7.4), and then, the cells incubated at 37 °C without CO_2_ for 45 min with DMEM assay medium to allow cells to pre-equilibrate. The oxygen consumption rate (OCR) measurements were then assessed using a Seahorse XF Cell Mito Stress Test Kit (Seahorse Bioscience). During the assay, we made the following at the final concentrations shown: 1.0 μM oligomycin (Oligo), 2.0 μM the uncoupler carbonyl cyanide 4-(trifluoromethoxy) phenylhydrazone (FCCP) and 0.5 μM rotenone/antimycin A (rot/ AA). After measurement, OCR was calculated by plotting the O_2_ tension of media as a function of time (pmol/min/10^4^ cells), and data were normalized by the number of cells measured in each individual well. Each sample was measured in four wells.

### siRNA transfection

hASCs were plated in growth medium 24 h prior to transfection. The siRNA transfection was performed following the manufacturer’s protocol as previously described [56]. Briefly, ON-TARGET SMARTpool siRNAs directed against human α5 integrin (L-008003-00-0005, Dharmacon, Lafayette, LA, USA) or non-targeting siRNAs (D-001810-10-05, Dharmacon) were mixed with Transfection DharmaFECT1 (Dharmacon). After a 20-min incubation at room temperature, the complexes were added to the cells at a final siRNA concentration of 50 nM. The medium was replenished with fresh medium for 24 h post-transfection. Experiments were performed 48–72 h after transfection.

### Quantitative RT-PCR

Quantitative RT-PCR was performed as previously described [57]. Total cellular RNA was extracted from 3.0 × 10^5^cells with the RNeasy Mini Kit (QIAGEN, Hilden, Germany), according to the manufacturer’s instructions. Reverse transcription of RNA was performed using Superscript III reverse transcriptase and random hexamer primers (Invitrogen). Real-time PCR analysis was performed on a Thermo Fisher Scientific applied Biosystems QuantStudio 5 system (Applied Biosystems) using the SYBR Green PCR Master Mix (Applied Biosystems). The reaction consisted of 10 μl of SYBR Green PCR Master Mix, 1 μl of a 5 μM mix of forward and reverse primers, 1 μl of template cDNA, and 8 μl of water in a total volume of 20 μl. Cycling was performed using the QuantStudio Design Analysis Software. The relative expression of each gene was normalized against 18S rRNA. The primers used are summarized in Additional file 1: Table S2.

### RNA sequencing and analysis

Approximately 1–2 µg of total RNA isolated from each individual biological sample was used for RNA sequencing library construction. We used NEB Next^®^ Poly(A) mRNA magnetic separation module (New England Biolabs) to isolate mRNA from total RNA. The KAPA Stranded RNA-Seq Library Prep kit (Illumina) was used for library construction. The quality of the library was analysed using Agilent 2100 Bioanalyzer. The library was sequenced using the Illumina NovaSeq 6000 platform by Aksomics Biotech Company (Shanghai, China).

Basecalls were performed using Solexa pipeline software (version 1.8). The quantity of trimmed reads (trimmed 5′,,3′-adaptor bases using cutadapt) was assessed using FastQC software (version 0.11.7). The trimmed reads were aligned to human reference genome GRCh37 (also known as hg19) using Hisat2 (2.1.0) software. Only unique mapping reads were considered for further analysis. FPKM calculations at the gene level and transcript level were performed using the R software Ballgown (version 2.8.4). Only a gene or transcript which expression means in each group was greater than or equal to 0.5 were used for further analysis. We defined the expressed genes which fold change ≥ 1.5, *p*-value ≤ 0.05, and FPKM ≥ 0.5 as differentially expressed gene and used differentially expressed gene data to perform gene set enrichment analysis (GSEA), Kyoto Encyclopedia of Genes and Genomes (KEGG) analysis and gene ontology (GO) analysis.

### Statistical analysis

Statistical analysis was performed using with GraphPad Prism 7 software. Statistically significant differences were assessed by an unpaired two-tailed Student’s *t*-test. The differences were considered significant if *p* < 0.05.

## Results

### Obesity increases native ECM accumulation in the vWAT microenvironment of hASCs

**Fig. 1:**
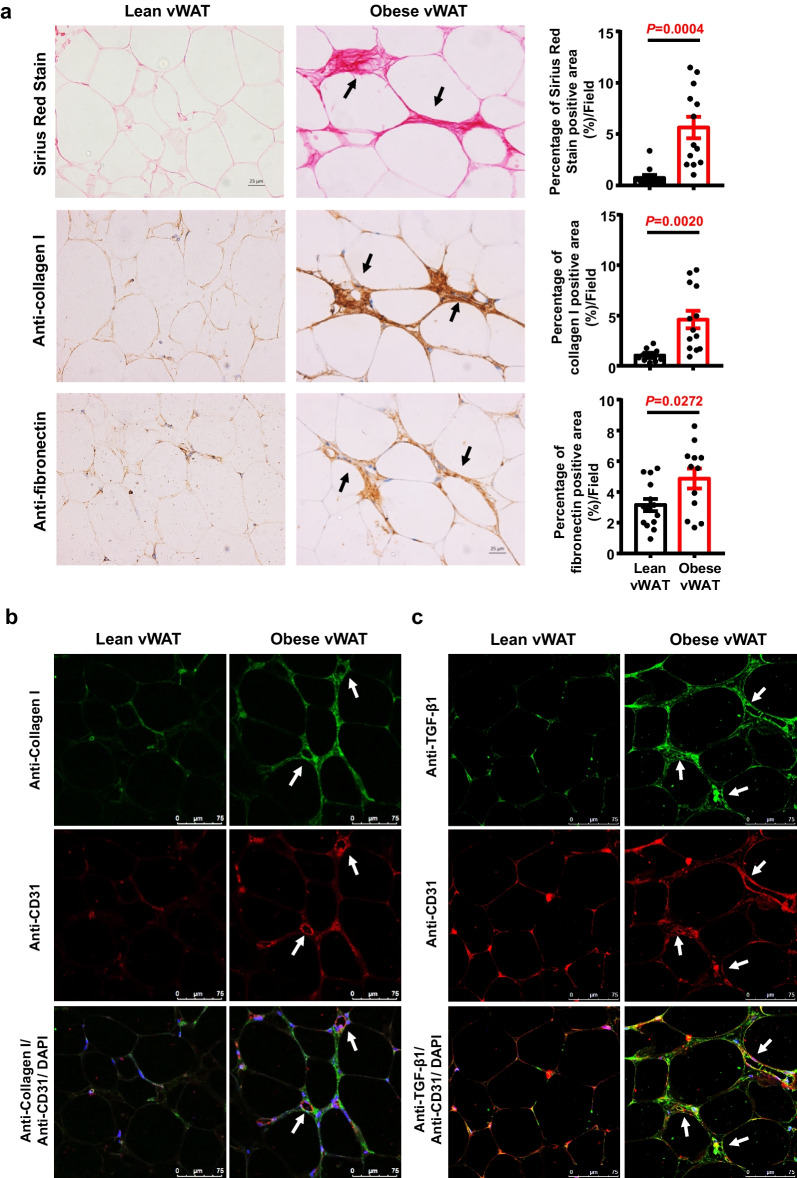
The properties of ECM in vWAT from lean and obese objects were determined. **a** Representative image of ECM for Sirius Red stain, immunostained with antibody against type I collagen, and fibronectin. The black arrows indicate staining positive area. Histogram showing the difference of the positive area percentage of Sirius Red, type I collagen, and fibronectin in each field from lean hASCs and obese hASCs. The number of fields was 10–14 in each group, respectively. Data are presented as the means ± SEM. Significant difference by unpaired two-tailed paired *t* test. Scale bars, 25 μm. **b** Representative image of ECM for immunostained with antibody against type I collagen (green) and CD31 (red) as well as DAPI nuclear staining (blue). The white arrows indicate staining positive area. Scale bars, 75 μm. **c** Representative image of ECM for immunostained with antibody against TGF-β1 (green) and CD31 (red) and DAPI nuclear staining (blue). The white arrows indicate staining positive area. Scale bars, 75 μm. vWAT, visceral white adipose tissue

To assess the alteration of the ECM components, the vWAT from lean and obese donors (Table 1) was determined. Histological evaluation of H&E-stained sections showed global differences in obese vWAT versus lean vWAT, including larger adipocytes in obese vWAT (Additional file 1: Figure S1). Sirius red staining analysis suggested that ECM components were significantly increased in obese vWAT compared with lean vWAT (Fig. 1a, upper panel). Immunohistochemical staining demonstrated that the fibres organized in density bundles surrounding adipocytes were type I collagen (Fig. 1a, middle panel) and fibronectin (Fig. 1a, lower panel). The protein levels of type I collagen and fibronectin were enhanced in obese vWAT compared with lean vWAT (Fig. 1a, right panel). These data suggest that native matrix deposits were significantly increased in vWAT derived from obese patients.

To further analyse the ECM microenvironment component of hASCs, coimmunofluorescence staining with anti-type I collagen and anti-CD31 or anti-TGF-β1 and anti-CD31 was performed. The results showed that abundant type I collagen fibres and TGF-β1, a critical factor for tissue fibrosis, were localized in SVF fractions in obese vWAT (Fig. 1b, c). These data indicated that the protein levels of type I collagen, fibronectin and TGF-β1 in the native microenvironment of hASCs were significantly increased in vWAT derived from obese patients compared with the lean control.

### Obese hASCs exhibit lower proliferation rates and senescence properties

To investigate cellular and molecular differences in obese and lean patients, the hASCs isolated from the vWAT of eight obese patients and three lean control donors were evaluated as previously described [50]. The majority of the cells from both lean control and obese donors exhibited the typical uniform spindle-shaped appearance of morphogenic fibroblasts at the 6th passage (Additional file 1: Figure S2A). To quantitatively assess the characteristics of the cells, they were further analysed by evaluating forward scatter (FSC, representing cell size) and side scatter (SSC, representing granularity in cells) using flow cytometry. The results showed that approximately 9.59% of the lean hASCs and 43.66% of the obese hASCs showed higher SSC in the upper left (UL) and upper right (UR) quadrants (Additional file 1: Figure S2B).

To define the phenotype of these fibroblast-like cells, the surface protein expression of hASCs was analysed with specific antibodies using flow cytometry. The results showed that both the obese and lean hASCs were positive for CD90, CD105, CD73, and negative for CD34 (Additional file 1: Figure S2C) and possessed the multipotent markers OCT4 (POU class 5 homeobox 1, POU5F1), NANOG (Nanog homeobox), SOX2 (SRY-box transcription factor 2), and SALL4 (spalt-like transcription factor 4) (Additional file 1: Figure S2D). The data confirmed that the hASCs from the obese patients maintained a similar morphology and phenotype to those from the control donors, as previously described [13, 50].

**Fig. 2:**
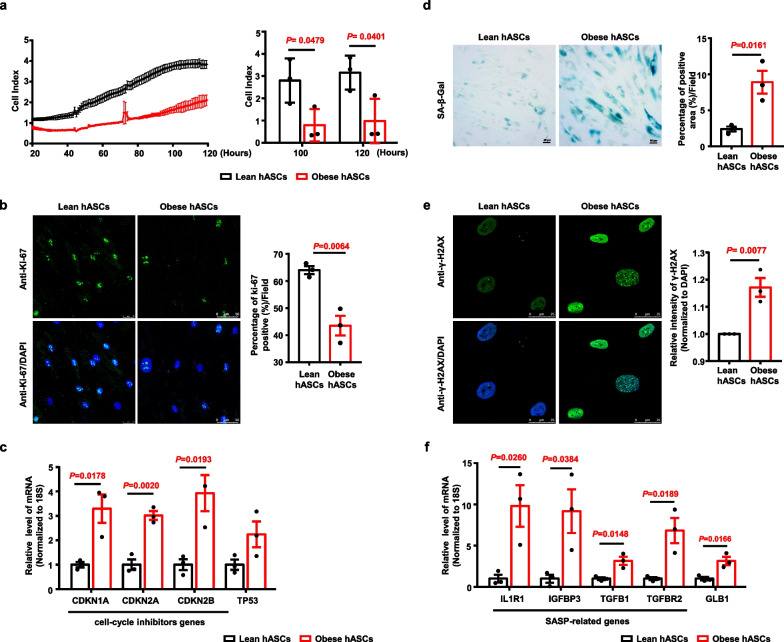
The properties of senescence in lean hASCs and obese hASCs were determined. **a** Graph showing the cell growth curve of hASCs from lean donor and obese donor monitored and recorded by Realtime xCELLigence analysis. Histogram showing the significant difference of the cell index of hASCs from two groups. *n* = 3 different donors. Error bars represent SD. **b** Representative image of hASCs in the two groups immunostained with antibody against Ki-67. Histogram showing the difference of the positive cell percentage of Ki-67 from lean hASCs and obese hASCs. The number of fields was 22–24 in each group, respectively. *n* = 3 different donors. Data are presented as the means ± SEM. Scale bars, 50 μm. **c** Relative mRNA levels of cell cycle inhibitor genes (*CDKN1A*, *CDKN2A*, *CDKN2B*, and *TP53*) in lean hASCs and obese hASCs were determined by quantitative RT-PCR. The relative expression of each gene was normalized against 18S rRNA. Data are presented as the means ± SEM. *n* = 3 different donors. **d** Representative image of hASCs in the two groups for SA-β-Gal staining. Histogram showing the difference of the positive area percentage of SA-β-Gal from lean hASCs and obese hASCs. The number of fields was 15–19 in each group, respectively. *n* = 3 different donors. Data are presented as the means ± SEM. Scale bars, 50 μm. **e** Representative images of hASCs from lean and obese donors for immunostained with antibody against γ-H2AX. Histogram showing the difference of the relative intensity of γ-H2AX normalized to DAPI each nuclear of cells. Fold represents the relative intensity was normalized against lean hASCs from each independent experiment. The number of cells for the expression of γ-H2AX was 204–309 in each group, respectively. *n* = 3 different donors. Data are presented as the means ± SEM. Scale bars, 25 μm. **f** Relative mRNA levels of SASP-related genes (*IL1R1*, *IGFBP3*,* TGFB1*, and *TGFBR2*), and GLB1 in lean hASCs and obese hASCs were determined by quantitative RT-PCR. The relative expression of each gene was normalized against 18S rRNA. Data are presented as the means ± SEM. *n* = 3 different donors. Significant difference by unpaired two-tailed paired t test

Next, the proliferation properties of hASCs from obese and lean donors were quantified by performing xCELLigence growth index analysis. The results showed that the cell index of obese hASCs was significantly decreased compared to that of lean hASCs at 100 and 120 h (Fig. 2a and Additional file 1: Figure S3A). The proliferative capacity of hASCs (expressed as slope) from the 20th hour to the 120th hour was significantly downregulated in obese hASCs (Additional file 1: Figure S3B). Immunofluorescence analysis showed that the percentage of Ki-67-positive cells in lean hASCs and obese hASCs was ~ 63% and ~ 43.5%, respectively (Fig. 2b). The results of quantitative RT-PCR indicated that the mRNA levels of cell cycle inhibitor genes, including *CDKN1A (cyclin-dependent kinase inhibitor 1A), CDKN2A (cyclin-dependent kinase inhibitor 2A),* and *CDKN2B (cyclin-dependent kinase inhibitor 2B),* but not *TP53 *(tumour protein p53)*,* in obese hASCs were significantly increased compared to those in lean hASCs (Fig. 2c). These data indicated that the proliferative capacity of obese hASCs was decreased compared to that of lean hASCs.

To further investigate whether the reduced proliferative capacity of obese hASCs is responsible for cellular senescence, SA-β-gal staining and immunofluorescence analysis for phosphorylated (γ) H2AX were performed. The results showed that the percentages of positively stained areas for SA-β-gal (Fig. 2d) and γ-H2AX (Fig. 2e) were significantly increased in obese hASCs compared to those in lean hASCs. In addition, the expression levels of SASP-related genes, including *IL1R1 *(*interleukin 1 receptor type 1*)*, IGFBP3 *(*insulin like growth factor binding protein 3*),* TGFB1 *(*transforming growth factor beta 1*)*, TGFBR2 *(*transforming growth factor beta receptor 2*), and the galactosidase beta 1 (*GLB1*) gene (a gene that encodes a member of the glycosyl hydrolase 35 family of proteins, a family of proteins that catalyse the hydrolysis of a terminal beta-linked galactose residue from ganglioside substrates and other glycoconjugates), were also significantly increased in obese hASCs compared to those in lean hASCs (Fig. 2f). These data indicated that obese hASCs show decreased proliferation rates and increased senescence properties compared to those of lean hASCs.

### α5 integrin may not be directly involved in senescence in obese hASCs

Senescence activation is highly dependent on the interactions of cells with ligands in the ECM [58]. α5 integrin (ITGA5), the major transmembrane ECM receptor of fibronectin protein [59], which is responsible for cell–matrix interactions [56], was then evaluated in obese hASCs and lean hASCs. The results showed that the distribution of α5 integrin was aggregated in obese hASCs, and the protein levels of α5 integrin were increased in obese hASCs compared to lean hASCs (Additional file 1: Figure S4A). The intranuclear distribution of YAP (Yes1-associated transcriptional regulator), the mechanosignal-sensitive nuclear transcription factor [60], which is correlated with actin cytoskeleton stability and cellular tension, was significantly increased in obese hASCs (Additional file 1: Figure S4A, lower panel).

Focal adhesions are a major hub for cell mechanosensing that act as a bridge mediating the interaction between the ECM and the cytoskeleton in cells [61]. To clarify the origin of the response of obese hASCs to ECM in adipose tissue, the component of focal adhesions was determined in obese and lean hASCs. The results showed that high α-SMA (alpha-smooth muscle actin) expression localized in stress fibres, exhibited activation of focal adhesion kinase and was present in obese hASCs. The colocalization of vinculin and F-actin was increased in obese hASCs compared to lean hASCs (Additional file 1: Figure S4B). These data suggested that α5 integrin may mediate the higher intracellular cytoskeletal tension and the obese hASC senescence response to the altered native ECM microenvironment in obese adipose tissue.

**Fig. 3:**
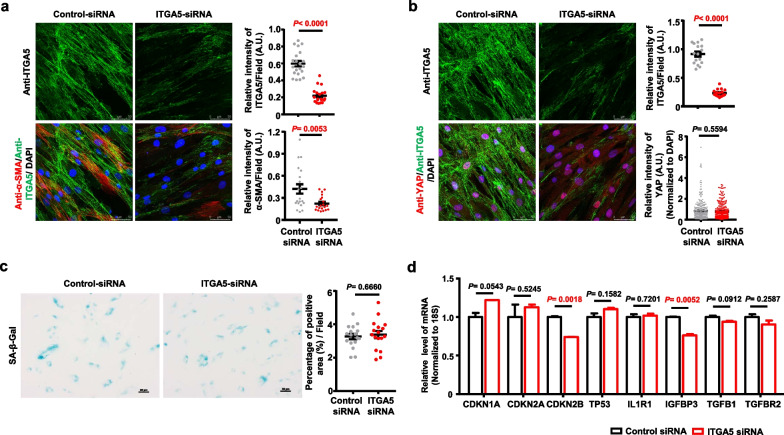
The effect of α5 integrin on senescence of the obese hASCs. **a** Representative images of the expression of α5 integrin and α-SMA in ITGA5 siRNA-treated and control siRNA-treated obese hASCs was determined by immunofluorescence. Histogram showing the difference of the relative intensity of α5 integrin and α-SMA in the field. The number of fields was 20–21 for the expression of α5 integrin and α-SMA in each group respectively. Data are presented as the means ± SEM. Scale bars, 50 μm. **b** Representative images of the expression of α5 integrin and YAP in ITGA5 siRNA-treated and control siRNA-treated obese hASCs was determined by immunofluorescence. Histogram showing the difference of the relative intensity of α5 integrin in the field and YAP normalized to DAPI each nuclear of cells. The number of fields for the expression of α5 integrin was 16–17 in each group respectively. The number of cells for the expression of YAP was 265–284 in each group respectively. Data are presented as the means ± SEM. Scale bars, 50 μm. **c** Representative images of SA-β-Gal staining in ITGA5 siRNA-treated obese hASCs and control siRNA-treated obese hASCs. Histogram showing the difference of the positive area percentage of SA-β-Gal. The number of fields for SA-β-Gal staining was 17 in each group respectively. Data are presented as the means ± SEM. Scale bars, 25 μm. **d** The relative mRNA levels of cell cycle inhibitors genes and SASP-related genes in ITGA5 siRNA-treated obese hASCs and control siRNA-treated obese hASCs were determined by quantitative RT-PCR. The relative expression of each gene was normalized against 18S rRNA. Data are presented as the means ± SEM. Significant difference by unpaired two-tailed paired t test. A.U. Arbitrary Unit; ITGA5, α5 integrin

To investigate the effect of α5 integrin on the senescence of obese hASCs, the expression of α5 integrin was knocked down in obese hASCs using α5 integrin siRNA (Additional file 1: Figure S5), and the properties of obese hASCs were determined. Immunofluorescence staining showed that α-SMA expression was significantly decreased in α5 integrin siRNA-treated cells, unlike in control siRNA-treated cells (Fig. 3a). However, the intranuclear distribution of YAP (Fig. 3b) and the percentage of positive areas for SA-β-gal staining (Fig. 3c) did not decrease in α5 integrin siRNA-treated cells compared with the control siRNA-treated cells.

The results of quantitative RT-PCR indicated that the mRNA levels of cell cycle inhibitors and SASP-related genes, except for *CDKN2B* and *IGFBP3*, did not change in α5 integrin siRNA-treated obese hASCs compared to those in the control siRNA-treated cells (Fig. 3d). These data indicated that α5 integrin may not be directly involved in the senescence of obese hASCs derived from patients.

### Obese hASCs exhibited ageing and ECM remodelling transcriptome properties

In an attempt to understand the mechanism of obese hASCs ageing, RNA sequencing was performed to obtain an initial perspective on global gene expression changes in obese hASCs and lean hASCs. The results revealed that 249 genes were significantly upregulated and 393 genes were significantly downregulated in the obese hASCs (Additional file 2: Table S3).

**Fig. 4:**
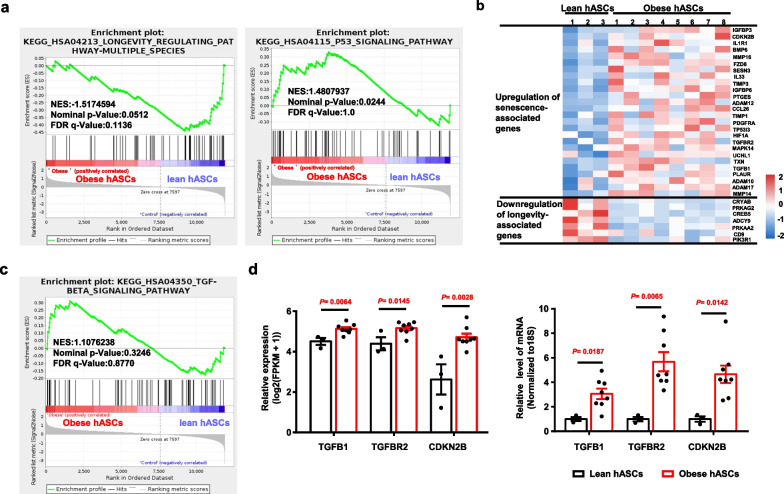
Comparative analysis of differentially expressed genes in the lean hASCs and obese hASCs based on RNA sequencing. **a** Graph showing GSEA for the KEGG pathways of the upregulated differentially expressed genes in the obese hASCs. Lean hASCs, *n* = 3 different donors. Obese hASCs, *n* = 8 different donors. **b** Heatmap showing the differential expression of senescence-associated genes (upper panel) and longevity-associated genes (lower panel) between the lean hASCs and obese hASCs. Higher expression is depicted in red, and lower expression is depicted in blue. The colour intensity represents the Row z score. Lean hASCs, *n* = 3 different donors. Obese hASCs, *n* = 8 different donors. **c** Graph showing GSEA revealed that genes enriched in the TGF-beta signalling pathway were upregulated in the obese hASCs. **d** Histogram showing the relative expression of TGFB1, TGFBR2 and CDKN2B was normalized log-scaled FPKM (log2 (FPKM + 1)) in lean hASCs and obese hASCs were determined by RNA sequencing (left panel). Histogram showing the relative mRNA expression levels of TGFB1, TGFBR2 and CDKN2B in lean hASCs and obese hASCs were determined by quantitative RT-PCR. The relative expression of each gene was normalized against 18S rRNA (righ panel). Lean hASCs, *n* = 3 different donors. Obese hASCs, *n* = 8 different donors. Data are presented as the means ± SEM. Significant difference by unpaired two-tailed paired *t* test. FPKM, fragments per kilobase of transcript per million mapped reads

GSEA revealed that the pathway related to “longevity regulating pathway-multiple species” (HSA04213) was significantly downregulated, while the “p53 signalling pathway” (HSA04115) was significantly upregulated (Fig. 4a). Genes involved in these pathways are listed in Additional file 2: Table S4. Comprehensive analysis of RNA sequencing data with age-related genes was extracted from https://ngdc.cncb.ac.cn/aging/age_related_genes. The results revealed that obese hASCs had 26 upregulated senescence-associated genes and 7 downregulated longevity-associated genes (Fig. 4b and Additional file 2: Table S4). Among the upregulated senescence-associated genes, *TGFB1*, *TGFBR2* and *CDKN2B* are involved in TGF-β signalling pathway (Fig. 4c). Relative mRNA levels of these genes in the lean hASCs and obese hASCs were determined by quantitative RT-PCR (Fig. 4d).

A functional gene annotation analysis of the identified upregulated differentially expressed genes between obese hASCs and lean hASCs was further analysed according to functional categories related to processes occurring during ECM remodelling. The results revealed that the significantly upregulated genes in obese hASCs were associated with the following functional groups: “extracellular matrix organization” (GO:0030198), “extracellular matrix assembly” (GO:0085029), “collagen fibril organization” (GO:0030199), “fibronectin binding” (GO:0001968), and “type I transforming growth factor beta receptor binding” (GO:0034713) (Additional file 1: Figure S6A and Additional file 2: Table S5).

The differentially expressed genes involved in three or more GO categories of extracellular matrix remodelling are reported in Additional file 1: Figure S6B, including tenascin XB (TNXB), fibulin 5 (FBLN5), fibulin 1 (FBLN1), TGFB1, fibulin 2 (FBLN2), TIMP metallopeptidase inhibitor 1 (TIMP1), type XVI collagen alpha 1 chain (COL16A1), laminin subunit gamma 2 (LAMC2), collagen triple helix repeat containing 1 (CTHRC1), endoglin (ENG), proteoglycan 4 (PRG4), and TIMP metallopeptidase inhibitor 3 (TIMP3). Relative mRNA levels of four genes of interest in the lean hASCs and obese hASCs were determined by quantitative RT-PCR (Additional file 1: Figure S6C). In Additional file 1: Figure S6D, the network view predicted the associations between the proteins encoded by the regulated genes.

These data indicated that obese hASCs exhibit increased senescence- and ECM remodelling-related transcriptome properties. TGF-β signalling pathway may play a key role in the ageing of obese hASCs and ECM remodelling in the native microenvironment of vWAT in obesity.

### Obese hASC-derived matrix partially mimics native ECM in adipose tissue from obesity patients

Given that the regulation of TGF-β signalling by ECM proteins [26] and stem cell-secreted matrix may mimic native ECM composition and structure in their resident tissue, we next tested whether the obesity-mediated effects on hASCs translate to altered hASC-derived matrix. hASCs were cultured on precoated glass dishes and were allowed to produce and deposit their substrate for 7 days. The cell layer was removed, and the underlying deposited matrix firmly attached to the glass dishes was left free of cell debris using an extraction procedure as previously described with modifications [41, 51, 52].

**Fig. 5:**
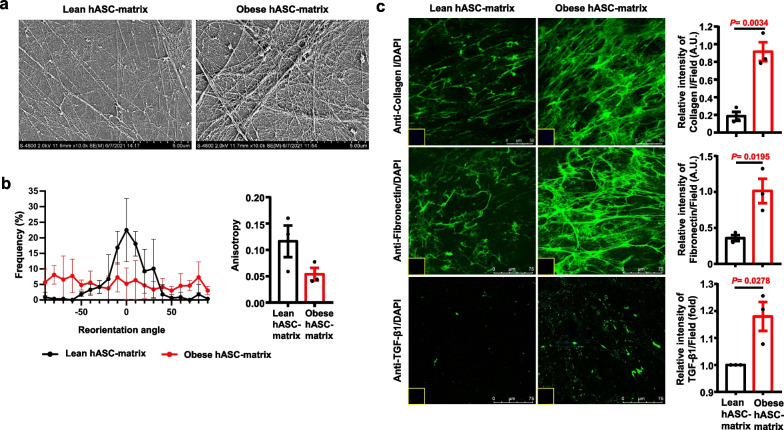
The characterization of the hASC-derived matrix response was determined. **a** Representative surface topography and microstructure images of hASC-derived matrix derived from lean and obese donors observed by SEM. Scale bars, 5 μm. **b** Graph showing the distribution of reorientation angle and anisotropy coefficient of the fibres in lean hASC-derived matrix and obese hASC-derived matrix determined by the ImageJ plug-in FibrilTool. The left panel is the distribution of normalized average fibre orientation (direction angle in degrees in the interval − 90–90° with respect to the major fibril direction of the image). The right panel represents anisotropy of fibre arrays in a given region of interest. 0 for no order (purely isotropic arrays) and 1 for perfectly ordered (parallel fibrils). *n* = 3 different donors. The number of fields for anisotropy was 12 in each group, respectively. **c** Representative images of lean hASC-derived matrix and obese hASC-derived matrix for immunostained with antibody against type I collagen, fibronectin, and TGF-β1. Histogram showing the difference of the relative intensity of type I collagen, fibronectin, and TGF-β1 in each field. *n* = 3 different donors from type I collagen and fibronectin, in which > 7 fields were quantified. Insets: The hASC-derived matrix was counterstained with DAPI. Data are presented as the means ± SEM. Scale bars, 50 μm and 75 μm Significant difference by unpaired two-tailed paired t test. A.U., Arbitrary Unit. Fold represents the relative intensity was normalized against lean hASC-derived matrix from each independent experiment. hASC-matrix, hASC-derived matrix

Then, the topography and the major components of the hASC-derived matrix in obese subjects and lean controls were determined. SEM analysis showed that the superstructure of the hASC-derived matrix was composed of a fibrillar network built on a layer of dense substance. The fibres on the obese hASC-derived matrix were more compact and aligned with multilayer density branch patterns within the matrix network than those on the lean hASC-derived matrix (Fig. 5a). To quantify the properties of the hASC-derived matrix, the main orientation and anisotropy of fibrillar structures were measured and assessed in images using the ImageJ plug-in FibrilTool [62] (Additional file 1: Figure S7). The results showed that the fibres in obese hASC-derived matrix were more randomly distributed than those in the lean hASC-derived matrix (Fig. 5b, left panel). Additionally, the decreased anisotropy coefficient also indicated a more random distribution of fibres in obese hASC-derived matrix (Fig. 5b, right panel).

Immunofluorescence staining showed that the type I collagen and fibronectin fibres in the obese hASC-derived matrix were denser and more reticulated than those in the lean hASC-derived matrix (Fig. 5c). Similarly, the intensity of TGF-β1 in the obese hASC-derived matrix was significantly higher than that in the lean hASC-derived matrix (Fig. 5c, lower panel). These findings indicated that the obese hASC-derived matrix partially mimics the native ECM in obese adipose tissue*.*

To investigate the response of hASCs to the obese hASC-derived matrix, lean hASCs were cultured on the obese hASC-derived matrix or lean hASC-derived matrix for 5 days, and the expression levels of integrin, F-actin, and YAP were then determined. Quantitative RT-PCR showed that the mRNA levels of α5 integrin, αV integrin (ITGAV), and β8 integrin (ITGB8) in lean hASCs cultured on the obese hASC-derived matrix were significantly increased compared to those of the lean hASCs cultured on the lean hASC-derived matrix (Additional file 1: Figure S8A). Immunofluorescence staining demonstrated that the protein levels of α5 integrin were significantly increased in lean hASCs when they were cultured on the obese hASC-derived matrix (Additional file 1: Figure S8B). Reinforced parallel F-actin stress fibres were observed in the lean hASCs cultured on the obese hASC-derived matrix (Additional file 1: Figure S8B). The intensity of YAP was significantly increased in the lean hASCs cultured on the obese hASC-derived matrix compared to those cultured on the lean hASC-derived matrix (Additional file 1: Figure S8C). Together, these findings indicated that the response of lean hASCs to the obese hASC-derived matrix is similar to that of obese hASCs in obese adipose tissue in vivo*.*

### Obese hASC-derived matrix promotes hASC senescence through TGF-β1 signalling

**Fig. 6:**
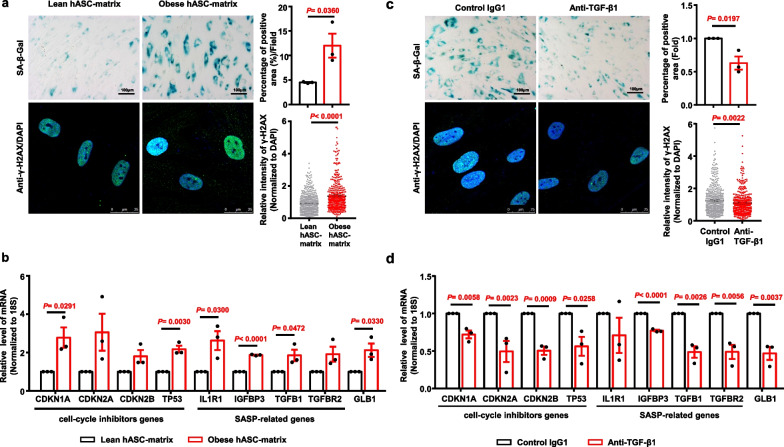
The senescence properties were determined in lean hASCs cultured on different hASC-derived matrixes. **a** Representative images of lean hASCs cultured on different hASC-derived matrix for SA-β-Gal staining and immunostained with antibody against γ-H2AX. Histogram showing the difference of the positive area percentage of SA-β-Gal and the relative intensity of γ-H2AX normalized to DAPI each nuclear of cells. The number of fields for SA-β-Gal staining was 14–23 in each group, respectively. *n* = 3 independent experiments from lean hASC-derived matrix, *n* = 3 different donors from obese hASC-derived matrix. Scale bars, 100 μm. The number of cells for the expression of γ-H2AX was 519 in lean hASC-derived matrix and 407 in obese hASC-derived matrix. Scale bars, 25 μm. **b** The relative mRNA levels of cell cycle inhibitors genes, SASP-related genes, and GLB1 in lean hASCs cultured on different ECMs were determined by quantitative RT-PCR. *n* = 3 independent experiments from lean hASC-derived matrix, *n* = 3 different donors from obese hASC-derived matrix. Fold represents that the relative expression of each gene was normalized against lean hASC-derived matrix from each independent experiment. **c** Representative images of lean hASCs cultured on the anti-TGF-β1 neutralizing antibody and control IgG1-treated obese hASC-derived matrix for SA-β-Gal staining and immunostained with antibody against γ-H2AX. Histogram showing the difference of the percentage of SA-β-Gal-positive area in each field, the percentage of SA-β-Gal-positive area was normalized against IgG1 group from each independent experiment. The number of fields was 16–24 for SA-β-Gal staining in each group, respectively. *n* = 3 different donors per group. Scale bars, 100 μm. Histogram showing the relative intensity of γ-H2AX normalized to DAPI each nuclear of cells. The number of cells for the expression of γ-H2AX was 491 in IgG1 group and 429 in anti-TGF-β1 neutralizing antibody group. Scale bars, 25 μm. Fold represents that the relative expression of each gene was normalized against control IgG1 group from each independent experiment. **d** The relative mRNA levels of cell cycle inhibitors genes, SASP-related genes, and GLB1 in lean hASCs cultured on different ECMs were determined by quantitative RT-PCR. Fold represents the relative expression of each gene was normalized against IgG1 group from each independent experiment. *n* = 3 different donors per group. Significant difference by unpaired two-tailed paired t test. Data are presented as the means ± SEM. hASC-matrix, hASC-derived matrix

To investigate whether the obese hASC-derived matrix affects the senescence of hASCs, lean hASCs were further evaluated by culturing on the obese hASC-derived matrix or lean hASC-derived matrix for 5 days. The results showed that the percentage of SA-β-Gal-positive in lean hASCs cultured on the lean hASC-derived matrix was ~ 4.67%, and this proportion significantly increased to ~ 16.82% when lean hASCs were cultured on obese hASC-derived matrix from three different donors (Fig. 6a). On the other hand, the percentage of SA-β-Gal-positive obese hASCs did not change when these cells were cultured on the lean hASC-derived matrix from two different donors compared with those cultured on the obese hASC-derived matrix (Additional file 1: Figure S9). Immunohistochemical analysis showed that the relative intensity of γ-H2AX in lean hASCs was significantly increased when they were cultured on the obese hASC-derived matrix (Fig. 6a, lower panel). The mRNA levels of cell cycle inhibitors, including *CDKN1A* and *TP53,* were significantly increased in lean hASCs cultured on the obese hASC-derived matrix compared to those cultured on the lean hASC-derived matrix (Fig. 6b). Similarly, the expression levels of SASP-related genes, including *IL1R1, IGFBP3, TGF-β1* and *GLB1*, were also significantly increased in lean hASCs cultured on the obese hASC-derived matrix compared to those cultured on the lean hASC-derived matrix (Fig. 6b).

Considering that the ECM provides a repository for TGF-β1, the effect of TGF-β1 signalling on hASC senescence induced by the obese hASC-derived matrix was further investigated. Lean hASCs cultured on the obese hASC-derived matrix treated with an anti-TGF-β1 neutralizing antibody and control IgG1 were characterized. The results showed that the percentage of SA-β-Gal-positive cells and the relative intensity of γ-H2AX in lean hASCs cultured on the anti-TGF-β1 neutralizing antibody-treated obese hASC-derived matrix were significantly decreased compared to those cultured on the IgG1-treated obese hASC-derived matrix (Fig. 6c). The mRNA levels of cell cycle inhibitors and SASP-related genes were significantly decreased in lean hASCs cultured on the anti-TGF-β1 neutralizing antibody-treated obese hASC-derived matrix compared to those cultured on the IgG1-treated obese hASC-derived matrix (Fig. 6d). Together, these findings indicated that blocking TGF-β1 signalling alleviates the hASC senescence induced by the obese hASC-derived matrix*.*

### Obese hASC-derived matrix promotes hASC senescence through TGF-β1-mediated mitochondrial dysfunction

**Fig. 7:**
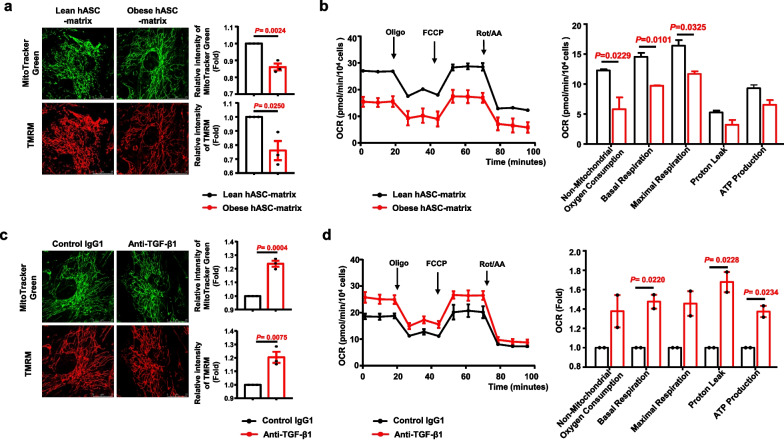
Mitochondrial function was determined in lean hASCs cultured on different hASC-derived matrix. **a** Representative images of lean hASCs cultured on different hASC-derived matrix for the mitochondrial mass and membrane potential determined by MitoTracker Green FM (Green) and TMRM (Red), respectively. Histogram showing the difference of the relative intensity of TMRM and MitoTracker Green in each cell from lean hASCs cultured on different hASC-derived matrix. The number of cells was 65–112 in each group, respectively. *n* = 3 independent experiments from one lean hASC-derived matrix; *n* = 3 different donors from obese hASC-derived matrix. Scale bars, 25 μm. Fold represents the relative intensity was normalized against lean hASC-derived matrix from each independent experiment. **b** Kinetic profile of the OCR was measured in lean hASCs cultured on different hASC-derived matrix using a Seahorse XF Cell Mito Stress test. The OCR was normalized against cells from each independent experiment. Black arrows show the times of treatment with Oligo, FCCP, Rot, and AA. Histogram showing the difference of the OCR of the lean hASC for different parameters from after cultured on different hASC-derived matrix. **c** Representative images of lean hASCs cultured on the anti-TGF-β1 neutralizing antibody and control IgG1-treated obese hASC-derived matrix for the mitochondrial mass and membrane potential determined by MitoTracker Green FM (Green) and TMRM (Red), respectively. Histogram showing the difference of the relative intensity of MitoTracker Green and TMRM in each cell from lean hASCs cultured on the anti-TGF-β1 neutralizing antibody and control IgG1-treated obese hASC-derived matrix. Fold represents the relative intensity was normalized against IgG1 group from each independent experiment. The number of cells was 51–112 in each group, respectively. *n* = 3 different donors. Scale bars, 25 μm. **d** Kinetic profile of the OCR was measured in lean hASCs cultured on the anti-TGF-β1 neutralizing antibody-treated obese hASC-derived matrix using a Seahorse XF Cell Mito Stress test. Fold represents the OCR was normalized against IgG1 group from each independent experiment. Black arrows show the times of treatment with Oligo, FCCP, Rot, and AA. Histogram showing the difference of the OCR of the lean hASC for different parameters from after cultured on different hASC-derived matrix. *n* = 2 different donors. Significant difference by unpaired two-tailed paired *t* test. Data are presented as the means ± SEM. OCR, oxygen consumption rate; oligo, oligomycin; FCCP, carbonyl cyanide 4-trifluoromethoxy-phenylhydrazone; rot: rotenone; AA, antimycin A; hASC-matrix, hASC-derived matrix; anti-TGF-β1, anti-TGF-β1 neutralizing antibody

Growing evidence indicates that mitochondrial dysfunction plays a critical role in regulating cellular senescence [63, 64]. To determine whether the obese hASC-derived matrix affects the mitochondrial function of hASCs, the mitochondrial mass, mitochondrial membrane potential, and bioenergetics status of lean hASCs cultured on the obese hASC-derived matrix or lean hASC-derived matrix were evaluated. The results showed that the relative intensities of MitoTracker Green FM (indicating mitochondrial mass) and TMRM (indicating mitochondrial membrane potential) in lean hASCs cultured on the obese hASC-derived matrix were significantly decreased compared to those of lean hASCs cultured on the lean hASC-derived matrix (Fig. 7a), which indicated a less-active mitochondrial state. The OCR-related basal respiration (representing oxygen consumption used to meet cellular ATP demand resulting from mitochondrial proton leak), maximal respiration, and nonmitochondrial (glycolysis) metabolism in lean hASCs cultured on the obese hASC-derived matrix were decreased compared to those of lean hASCs cultured on the lean hASC-derived matrix (Fig. 7b).

However, the mitochondrial mass and mitochondrial membrane potential were significantly increased in lean hASCs cultured on the anti-TGF-β1 neutralizing antibody-treated obese hASC-derived matrix compared to those cultured on the control IgG1-treated obese hASC-derived matrix (Fig. 7c). OCR-related basal respiration, proton leak (representing a mechanism to regulate mitochondrial ATP production) and ATP production (representing the mitochondria that contribute to meeting the energetic needs of the cell) were significantly increased in lean hASCs cultured on anti-TGF-β1 neutralizing antibody-treated obese hASC-derived matrix compared to those cultured on the control IgG1-treated obese hASC-derived matrix (Fig. 7d). These data indicated that TGF-β1 signalling plays a key role in promoting hASC senescence by impairing mitochondrial function mediated by the obese hASC-derived matrix. Blocked TGF-β1 signalling may partially recuse injured mitochondrial function.

Altogether, we found that the native ECM deposits in hASC microenvironments were increased in vWAT from obese patients. The obese hASC-derived matrix mimics the native ECM niche of stem cells in obese adipose tissue, which induces higher intracellular tension in lean hASCs. Obese hASC-derived matrix promotes lean hASC senescence accompanied by mitochondrial dysfunction. Blockade of TGF-β signalling alleviates lean hASC senescence and mitochondrial dysfunction induced by the obese hASC-derived matrix.

## Discussion

vWAT expansion and ECM remodelling directly impact the risk of developing metabolic syndrome in obesity. However, subcutaneous WAT expansion is protective [4]. Understanding the mechanism of cell-ECM microenvironment interactions in obesity-induced senescence of hASCs derived from vWAT may provide opportunities to uncouple obesity from metabolic disease. In the present study, we demonstrated that TGF-β1 signalling in the obese hASC-derived matrix plays a key role in promoting hASC senescence by impairing mitochondrial function. Blocked TGF-β1 signalling may partially recuse the injured mitochondrial function.

Evidence indicates that obesity triggers ECM remodelling in adipose tissue [1, 6, 11]. Our results confirmed that the ECM microenvironment of hASCs in the vWAT from obese patients contains higher amounts of type I collagen, fibronectin, and TGF-β1 than lean adipose tissue. Meanwhile, we revealed that hASCs from the vWAT of obese patients exhibited senescence properties, consistent with research on ASCs from human subcutaneous WAT [47, 65, 66].

Previous studies have indicated that cellular responses to pericellular ECM stiffness and composition are mediated by integrin assembly, focal adhesion, cytoskeletal reorganization, and nuclear translocation of YAP [67, 68]. Although the upregulation of α5 integrin and nuclear YAP and higher intracellular tension were found in obese hASCs, reflecting the responses of hASCs to ECM cues in obese adipose tissue, knocking down the expression of α5 integrin did not directly decrease the senescence of hASCs derived from obese patients. These results suggest that the altered behaviour of hASCs is associated with the influence of the ECM microenvironment components of obese adipose tissue.

Cumulative evidence indicates that ECM components secreted by hASCs have ideal physicochemical properties that closely mimic the native ECM microenvironment [37, 41]. To determine whether matrix components secreted by obese hASCs are different from those secreted by lean hASCs, we analysed the signalling pathways closely associated with matrix remodelling and the secreted ECM in vitro by employing the decellularization approach. The data showed that fibronectin, type I collagen, and TGF-β1 were more abundant in the obese hASC-derived matrix than in the lean hASC-derived matrix. These results suggested that obesity affects the protein composition remodelling of the hASC-derived matrix. Similar to the response of hASCs to obese adipose tissue in vivo, lean hASCs cultured on the obese hASC-derived matrix displayed higher expression of α5 integrin and YAP as well as higher intracellular tension than those cultured on the lean hASC-derived matrix. Taken together, these results indicated that the hASC-derived matrix mimics the native environment of hASCs in vivo and better facilitates the study of adipose tissue microenvironmental effects on hASC senescence in vitro.

These findings prompted us to investigate whether the obese hASC-derived matrix induces hASC senescence. Our findings showed that the percentage of aged lean hASCs was significantly increased by culturing on the obese hASC-derived matrix. Mitochondria play an important role in maintaining cell function, and cellular senescence is accompanied by mitochondrial dysfunction [47, 69, 70]. The present study showed that the mitochondrial mass, mitochondrial membrane potential, and OCR in lean hASCs were reduced by the obese hASC-derived matrix. Overall, these findings suggest that the properties of the microenvironment play a critical role in facilitating cell senescence, which may contribute to mitochondrial dysfunction.

TGF-β1 is an important component of the ECM; it can be secreted out of the cell and stored in the ECM in a latent state, and activation of TGF-β1 in the ECM presupposes binding to integrins containing RGD sites on the cell surface, such as αV integrin and α5 integrin [26, 71]. The present study found that TGF-β1 was more abundant in the obese hASC-derived matrix than in the lean hASC-derived matrix. Despite studies demonstrating the ability of soluble TGF-β1 to promote cellular senescence in vitro [58], few studies have reported that TGF-β1 in obese hASC-derived matrix promotes lean hASC senescence. The present study revealed that lean hASCs cultured on the anti-TGF-β1 neutralizing antibody-treated obese hASC-derived matrix exhibited fewer senescent cells and the upregulation of mitochondrial mass and mitochondrial function. This result suggested that inhibition of TGF-β1 in the obese hASC-derived matrix partially suppressed mitochondrial impairment, thereby delaying cellular senescence. Due to the complex composition and structure of the obese hASC-derived matrix, there may be multiple pathways that can disrupt mitochondrial bioenergetics, which requires further study.

## Conclusion

In this study, we demonstrated that obesity drives the senescence of hASCs and native ECM deposits in vWAT. The matrix secreted by obese hASCs causes cellular senescence and mitochondrial damage through TGF-β1 signalling. Thus, the present findings suggest that TGF-β1 is a putative therapeutic target and that mitochondria are a key element of the obese adipose tissue microenvironment affecting the senescence phenotype of hASCs. Moreover, further studies will be need to determine whether cytoskeletal tension has a direct effect on cellular senescence.

### Supplementary Information

**Additional file 1.** Supplementary Figures and Table S1, S2 of Blockade of TGF-β signalling alleviates human adipose stem cell senescence induced by native ECM in obesity visceral white adipose tissue.

**Additional file 2.** Supplementary Table S3, S4 and S5 of Blockade of TGF-β signalling alleviates human adipose stem cell senescence induced by native ECM in obesity visceral white adipose tissue.

## Data Availability

The RNA sequencing data have been deposited at GEO (GSE203371) and are publicly available as of the date of publication, repository at https://www.ncbi.nlm.nih.gov/geo/query/acc.cgi?acc=GSE203371. The datasets used and/or analysed in this study are available from the corresponding author on reasonable request.

## Abbreviations

ATP: Adenosine triphosphate
BMI: Body mass index
CDKN1A: Cyclin-dependent kinase inhibitor 1A
CDKN2A: Cyclin-dependent kinase inhibitor 2A
CDKN2B: Cyclin-dependent kinase inhibitor 2B
COL16A1: Type XVI collagen alpha 1 chain
CTHRC1: Collagen triple helix repeat containing 1
DAPI: 4’, 6-Diamidino-2-phenylindole
ECM: Extracellular matrix
EDTA: Ethylene diamine tetraacetic acid
ENG: Endoglin
FBLN1: Fibulin 1
FBLN2: Fibulin 2
FBLN5: Fibulin 5
FBS: Foetal bovine serum
FCCP: Carbonyl cyanide 4-trifluoromethoxy-phenylhydrazone
FITC: Fluorescein isothiocyanate
FPKM: Fragments per kilobase million
GEO: Gene Expression Omnibus database
GLB1: Galactosidase beta 1
GO: Gene ontology
GSEA: Gene set enrichment analysis
hASC: Human adipose stem cell
hASC-matrix: Human adipose stem cell-derived matrix
HOMA-IR: Homeostasis model assessment of insulin resistance
H&E: Haematoxylin–eosin
IGFBP3: Insulin like growth factor binding protein 3
IL1R1: Interleukin 1 receptor type 1
ITGA5: α5 integrin
ITGAV: αV integrin
ITGB8: β8 integrin
KEGG: Kyoto Encyclopedia of Genes and Genomes
LAMC2: Laminin subunit gamma 2
NANOG: Nanog homeobox
OCR: Oxygen consumption rate
OCT4: Organic cation/carnitine transporter 4
Oligo: Oligomycin
PBS: Phosphate-buffered saline
PE: Phycoerythrin
PRG4: Proteoglycan 4
Rot: Rotenone
RTCA: Real-time cellular analysis
RT-PCR: Reverse transcription-polymerase chain reaction
SALL4: Spalt-like transcription factor 4
SASP: Senescence-associated secretory phenotype
SA-β-Gal: Senescence β-galactosidase
SEM: Scanning electron microscopy
siRNA: Small interfering RNA
SOX2: SRY-box transcription factor 2
SVF: Stromal vascular fraction
TGFBR2: Transforming growth factor beta receptor 2
TGF-β1: Transforming growth factor-β1
TIMP1: TIMP metallopeptidase inhibitor 1
TIMP3: TIMP metallopeptidase inhibitor 3
TMRM: Tetramethylrhodamine methyl ester
TNXB: Tenascin XB
TP53: Tumour protein p53
vWAT: Visceral white adipose tissue
X-Gal: X-galactosidase
YAP: Yes-associated protein
α-SMA: Alpha-smooth muscle actin
γ-H2AX: Gamma H2A histone family member X

## References

[1] Chouchani ET, Kajimura S (2019). Metabolic adaptation and maladaptation in adipose tissue. Nat Metab.

[2] Jaacks LM, Vandevijvere S, Pan A, McGowan CJ, Wallace C, Imamura F, Mozaffarian D, Swinburn B, Ezzati M (2019). The obesity transition: stages of the global epidemic. Lancet Diabetes Endocrinol.

[3] Bluher M (2019). Obesity: global epidemiology and pathogenesis. Nat Rev Endocrinol.

[4] Vishvanath L, Gupta RK (2019). Contribution of adipogenesis to healthy adipose tissue expansion in obesity. J Clin Invest.

[5] Longo M, Zatterale F, Naderi J, Parrillo L, Formisano P, Raciti GA, Beguinot F, Miele C (2019). Adipose tissue dysfunction as determinant of obesity-associated metabolic complications. Int J Mol Sci.

[6] Marcelin G, Silveira ALM, Martins LB, Ferreira AV, Clement K (2019). Deciphering the cellular interplays underlying obesity-induced adipose tissue fibrosis. J Clin Invest.

[7] Neeland IJ, Ross R, Després J-P, Matsuzawa Y, Yamashita S, Shai I, Seidell J, Magni P, Santos RD, Arsenault B (2019). Visceral and ectopic fat, atherosclerosis, and cardiometabolic disease: a position statement. Lancet Diabetes Endocrinol.

[8] Sun K, Kusminski CM, Scherer PE (2011). Adipose tissue remodeling and obesity. J Clin Invest.

[9] Quail DF, Dannenberg AJ (2019). The obese adipose tissue microenvironment in cancer development and progression. Nat Rev Endocrinol.

[10] Sun K, Tordjman J, Clement K, Scherer PE (2013). Fibrosis and adipose tissue dysfunction. Cell Metab.

[11] Crewe C, An YA, Scherer PE (2017). The ominous triad of adipose tissue dysfunction: inflammation, fibrosis, and impaired angiogenesis. J Clin Invest.

[12] Cawthorn WP, Scheller EL, MacDougald OA (2012). Adipose tissue stem cells: the great WAT hope. Trends Endocrinol Metab.

[13] Li X, Yuan J, Li W, Liu S, Hua M, Lu X, Zhang H (2014). Direct differentiation of homogeneous human adipose stem cells into functional hepatocytes by mimicking liver embryogenesis. J Cell Physiol.

[14] Sakers A, De Siqueira MK, Seale P, Villanueva CJ (2022). Adipose-tissue plasticity in health and disease. Cell.

[15] Zuk PA, Zhu M, Mizuno H, Huang J, Futrell JW, Katz AJ, Benhaim P, Lorenz HP, Hedrick MH (2001). Multilineage cells from human adipose tissue: implications for cell-based therapies. Tissue Eng.

[16] Emont MP, Jacobs C, Essene AL, Pant D, Tenen D, Colleluori G, Di Vincenzo A, Jorgensen AM, Dashti H, Stefek A (2022). A single-cell atlas of human and mouse white adipose tissue. Nature.

[17] Merrick D, Sakers A, Irgebay Z, Okada C, Calvert C, Morley MP, Percec I, Seale P (2019). Identification of a mesenchymal progenitor cell hierarchy in adipose tissue. Science.

[18] Isakson P, Hammarstedt A, Gustafson B, Smith U (2009). Impaired preadipocyte differentiation in human abdominal obesity: role of Wnt, tumor necrosis factor-alpha, and inflammation. Diabetes.

[19] Louwen F, Ritter A, Kreis NN, Yuan J (2018). Insight into the development of obesity: functional alterations of adipose-derived mesenchymal stem cells. Obes Rev.

[20] Shin S, El-Sabbagh AS, Lukas BE, Tanneberger SJ, Jiang Y (2020). Adipose stem cells in obesity: challenges and opportunities. Biosci Rep.

[21] Serena C, Keiran N, Ceperuelo-Mallafre V, Ejarque M, Fradera R, Roche K, Nunez-Roa C, Vendrell J, Fernandez-Veledo S (2016). Obesity and type 2 diabetes alters the immune properties of human adipose derived stem cells. Stem Cells.

[22] Strong AL, Bowles AC, Wise RM, Morand JP, Dutreil MF, Gimble JM, Bunnell BA (2016). Human adipose stromal/stem cells from obese donors show reduced efficacy in halting disease progression in the experimental autoimmune encephalomyelitis model of multiple sclerosis. Stem Cells.

[23] Ejarque M, Ceperuelo-Mallafre V, Serena C, Maymo-Masip E, Duran X, Diaz-Ramos A, Millan-Scheiding M, Nunez-Alvarez Y, Nunez-Roa C, Gama P (2019). Adipose tissue mitochondrial dysfunction in human obesity is linked to a specific DNA methylation signature in adipose-derived stem cells. Int J Obes (Lond).

[24] Ermolaeva M, Neri F, Ori A, Rudolph KL (2018). Cellular and epigenetic drivers of stem cell ageing. Nat Rev Mol Cell Biol.

[25] Ren R, Ocampo A, Liu G-H, Izpisua Belmonte JC (2017). Regulation of stem cell aging by metabolism and epigenetics. Cell Metab.

[26] Hynes RO (2009). The extracellular matrix: not just pretty fibrils. Science.

[27] Brizzi MF, Tarone G, Defilippi P (2012). Extracellular matrix, integrins, and growth factors as tailors of the stem cell niche. Curr Opin Cell Biol.

[28] Chaudhuri O, Cooper-White J, Janmey PA, Mooney DJ, Shenoy VB (2020). Effects of extracellular matrix viscoelasticity on cellular behaviour. Nature.

[29] Sart S, Jeske R, Chen X, Ma T, Li Y (2020). Engineering stem cell-derived extracellular matrices: decellularization, characterization, and biological function. Tissue Eng Part B Rev.

[30] Prewitz MC, Seib FP, von Bonin M, Friedrichs J, Stissel A, Niehage C, Muller K, Anastassiadis K, Waskow C, Hoflack B (2013). Tightly anchored tissue-mimetic matrices as instructive stem cell microenvironments. Nat Methods.

[31] Lai Y, Sun Y, Skinner CM, Son EL, Lu Z, Tuan RS, Jilka RL, Ling J, Chen XD (2010). Reconstitution of marrow-derived extracellular matrix ex vivo: a robust culture system for expanding large-scale highly functional human mesenchymal stem cells. Stem Cells Dev.

[32] Rakian R, Block TJ, Johnson SM, Marinkovic M, Wu J, Dai Q, Dean DD, Chen XD (2015). Native extracellular matrix preserves mesenchymal stem cell “stemness” and differentiation potential under serum-free culture conditions. Stem Cell Res Ther.

[33] Riis S, Stensballe A, Emmersen J, Pennisi CP, Birkelund S, Zachar V, Fink T (2016). Mass spectrometry analysis of adipose-derived stem cells reveals a significant effect of hypoxia on pathways regulating extracellular matrix. Stem Cell Res Ther.

[34] Guneta V, Zhou Z, Tan NS, Sugii S, Wong MTC, Choong C (2017). Recellularization of decellularized adipose tissue-derived stem cells: role of the cell-secreted extracellular matrix in cellular differentiation. Biomater Sci.

[35] Chen XD, Dusevich V, Feng JQ, Manolagas SC, Jilka RL (2007). Extracellular matrix made by bone marrow cells facilitates expansion of marrow-derived mesenchymal progenitor cells and prevents their differentiation into osteoblasts. J Bone Miner Res.

[36] Marinkovic M, Tran ON, Block TJ, Rakian R, Gonzalez AO, Dean DD, Yeh CK, Chen XD (2020). Native extracellular matrix, synthesized ex vivo by bone marrow or adipose stromal cells, faithfully directs mesenchymal stem cell differentiation. Matrix Biol Plus.

[37] Ragelle H, Naba A, Larson BL, Zhou F, Prijic M, Whittaker CA, Del Rosario A, Langer R, Hynes RO, Anderson DG (2017). Comprehensive proteomic characterization of stem cell-derived extracellular matrices. Biomaterials.

[38] Zeitouni S, Krause U, Clough BH, Halderman H, Falster A, Blalock DT, Chaput CD, Sampson HW, Gregory CA (2012). Human mesenchymal stem cell-derived matrices for enhanced osteoregeneration. Sci Transl Med.

[39] Hoch AI, Mittal V, Mitra D, Vollmer N, Zikry CA, Leach JK (2016). Cell-secreted matrices perpetuate the bone-forming phenotype of differentiated mesenchymal stem cells. Biomaterials.

[40] Marinkovic M, Block TJ, Rakian R, Li Q, Wang E, Reilly MA, Dean DD, Chen XD (2016). One size does not fit all: developing a cell-specific niche for in vitro study of cell behavior. Matrix Biol.

[41] Riis S, Hansen AC, Johansen L, Lund K, Pedersen C, Pitsa A, Hyldig K, Zachar V, Fink T, Pennisi CP (2020). Fabrication and characterization of extracellular matrix scaffolds obtained from adipose-derived stem cells. Methods.

[42] Seo BR, Bhardwaj P, Choi S, Gonzalez J, Andresen Eguiluz RC, Wang K, Mohanan S, Morris PG, Du B, Zhou XK (2015). Obesity-dependent changes in interstitial ECM mechanics promote breast tumorigenesis. Sci Transl Med.

[43] Springer NL, Iyengar NM, Bareja R, Verma A, Jochelson MS, Giri DD, Zhou XK, Elemento O, Dannenberg AJ, Fischbach C (2019). Obesity-associated extracellular matrix remodeling promotes a macrophage phenotype similar to tumor-associated macrophages. Am J Pathol.

[44] De Girolamo L, Stanco D, Salvatori L, Coroniti G, Arrigoni E, Silecchia G, Russo MA, Niada S, Petrangeli E, Brini AT (2013). Stemness and osteogenic and adipogenic potential are differently impaired in subcutaneous and visceral adipose derived stem cells (ASCs) isolated from obese donors. Int J Immunopathol Pharmacol.

[45] Roldan M, Macias-Gonzalez M, Garcia R, Tinahones FJ, Martin M (2011). Obesity short-circuits stemness gene network in human adipose multipotent stem cells. FASEB J.

[46] Perez LM, Bernal A, de Lucas B, San Martin N, Mastrangelo A, Garcia A, Barbas C, Galvez BG (2015). Altered metabolic and stemness capacity of adipose tissue-derived stem cells from obese mouse and human. PLoS ONE.

[47] Alicka M, Major P, Wysocki M, Marycz K (2019). Adipose-derived mesenchymal stem cells isolated from patients with type 2 diabetes show reduced “stemness” through an altered secretome profile, impaired anti-oxidative protection, and mitochondrial dynamics deterioration. J Clin Med.

[48] Pachon-Pena G, Serena C, Ejarque M, Petriz J, Duran X, Oliva-Olivera W, Simo R, Tinahones FJ, Fernandez-Veledo S, Vendrell J (2016). Obesity determines the immunophenotypic profile and functional characteristics of human mesenchymal stem cells from adipose tissue. Stem Cells Transl Med.

[49] Onate B, Vilahur G, Camino-Lopez S, Diez-Caballero A, Ballesta-Lopez C, Ybarra J, Moscatiello F, Herrero J, Badimon L (2013). Stem cells isolated from adipose tissue of obese patients show changes in their transcriptomic profile that indicate loss in stemcellness and increased commitment to an adipocyte-like phenotype. BMC Genom.

[50] Li Y, Lin Y, Han X, Li W, Yan W, Ma Y, Lu X, Huang X, Bai R, Zhang H (2021). GSK3 inhibitor ameliorates steatosis through the modulation of mitochondrial dysfunction in hepatocytes of obese patients. iScience.

[51] Guo X, Li W, Ma M, Lu X, Zhang H (2017). Endothelial cell-derived matrix promotes the metabolic functional maturation of hepatocyte via integrin-Src signalling. J Cell Mol Med.

[52] Tello M, Spenle C, Hemmerle J, Mercier L, Fabre R, Allio G, Simon-Assmann P, Goetz JG (2016). Generating and characterizing the mechanical properties of cell-derived matrices using atomic force microscopy. Methods.

[53] Yan X, He Y, Yang S, Zeng T, Hua Y, Bao S, Yang F, Duan N, Sun C, Liang Y (2022). A positive feedback loop: RAD18-YAP-TGF-beta between triple-negative breast cancer and macrophages regulates cancer stemness and progression. Cell Death Discov.

[54] Sano S, Horitani K, Ogawa H, Halvardson J, Chavkin NW, Wang Y, Sano M, Mattisson J, Hata A, Danielsson M (2022). Hematopoietic loss of Y chromosome leads to cardiac fibrosis and heart failure mortality. Science.

[55] Arena ET, Rueden CT, Hiner MC, Wang S, Yuan M, Eliceiri KW (2017). Quantitating the cell: turning images into numbers with ImageJ. Wiley Interdiscip Rev Dev Biol.

[56] Ma Y, Zhang W, Li W, Lu X, Li Y, Han X, Zhang H (2022). α5 integrin regulates hepatic tight junctions through SRC-TET1-mediated DNA hydroxymethylation. iScience.

[57] Ma Y, Ma M, Sun J, Li W, Li Y, Guo X, Zhang H (2019). CHIR-99021 regulates mitochondrial remodelling via beta-catenin signalling and miRNA expression during endodermal differentiation. J Cell Sci.

[58] Rapisarda V, Borghesan M, Miguela V, Encheva V, Snijders AP, Lujambio A, O'Loghlen A (2017). Integrin Beta 3 regulates cellular senescence by activating the TGF-beta pathway. Cell Rep.

[59] Schumacher S, Dedden D, Nunez RV, Matoba K, Takagi J, Biertumpfel C, Mizuno N (2021). Structural insights into integrin alpha5beta1 opening by fibronectin ligand. Sci Adv.

[60] Panciera T, Azzolin L, Cordenonsi M, Piccolo S (2017). Mechanobiology of YAP and TAZ in physiology and disease. Nat Rev Mol Cell Biol.

[61] Case LB, Baird MA, Shtengel G, Campbell SL, Hess HF, Davidson MW, Waterman CM (2015). Molecular mechanism of vinculin activation and nanoscale spatial organization in focal adhesions. Nat Cell Biol.

[62] Boudaoud A, Burian A, Borowska-Wykret D, Uyttewaal M, Wrzalik R, Kwiatkowska D, Hamant O (2014). FibrilTool, an ImageJ plug-in to quantify fibrillar structures in raw microscopy images. Nat Protoc.

[63] Kornicka K, Szlapka-Kosarzewska J, Smieszek A, Marycz K (2019). 5-Azacytydine and resveratrol reverse senescence and ageing of adipose stem cells via modulation of mitochondrial dynamics and autophagy. J Cell Mol Med.

[64] Huang X, Zhang H, Liang X, Hong Y, Mao M, Han Q, He H, Tao W, Jiang G, Zhang Y (2019). Adipose-derived mesenchymal stem cells isolated from patients with abdominal aortic aneurysm exhibit senescence phenomena. Oxid Med Cell Longev.

[65] Gustafson B, Nerstedt A, Smith U (2019). Reduced subcutaneous adipogenesis in human hypertrophic obesity is linked to senescent precursor cells. Nat Commun.

[66] Conley SM, Hickson LJ, Kellogg TA, McKenzie T, Heimbach JK, Taner T, Tang H, Jordan KL, Saadiq IM, Woollard JR (2020). Human obesity induces dysfunction and early senescence in adipose tissue-derived mesenchymal stromal/stem cells. Front Cell Dev Biol.

[67] Stanton AE, Tong X, Yang F (2019). Extracellular matrix type modulates mechanotransduction of stem cells. Acta Biomater.

[68] Talele NP, Fradette J, Davies JE, Kapus A, Hinz B (2015). Expression of alpha-smooth muscle actin determines the fate of mesenchymal stromal cells. Stem Cell Rep.

[69] Li X, Hong Y, He H, Jiang G, You W, Liang X, Fu Q, Han S, Lian Q, Zhang Y (2019). FGF21 mediates mesenchymal stem cell senescence via regulation of mitochondrial dynamics. Oxid Med Cell Longev.

[70] Mansell E, Sigurdsson V, Deltcheva E, Brown J, James C, Miharada K, Soneji S, Larsson J, Enver T (2021). Mitochondrial potentiation ameliorates age-related heterogeneity in hematopoietic stem cell function. Cell Stem Cell.

[71] Rifkin DB, Rifkin WJ, Zilberberg L (2018). LTBPs in biology and medicine: LTBP diseases. Matrix Biol.

