# Supplementary material for: Blockade of TGF-β signalling alleviates human adipose stem cell senescence induced by native ECM in obesity visceral white adipose tissue
Source: Stem Cell Res Ther. 2023 Oct 8;14:291. doi: 10.1186/s13287-023-03525-y (PMC10561428; doi:10.1186/s13287-023-03525-y)
Supplement: Supplementary file 1 — Additional file 1. Supplementary Figures and Table S1, S2 of Blockade of TGF-β signalling alleviates human adipose stem cell senescence induced by native ECM in obesity visceral white adipose tissue. [file 13287_2023_3525_MOESM1_ESM.pdf]

Blockade of TGF-β signalling alleviates human adipose stem cell senescence induced by native ECM in obesity visceral white adipose tissue

Supplementary Figures and Figure legends

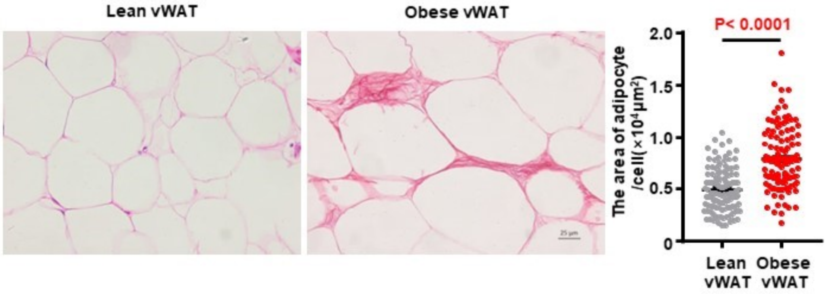

Figure S1 Adipocytes is larger in obese vWAT compared to lean vWAT. Related to Figure 1. Representative image of the morphology of vWAT in lean and obese donors was evaluated by HE staining. Histogram showing the difference of the area of adipocyte in adipose tissue from lean and obese donors. The number of adipocytes was 116 in lean vWAT and 120 in obese vWAT. Data are presented as the means±SEM. Significant difference by unpaired two-tailed paired t test. Scale bars, 25 μm. vWAT, visceral white adipose tissue.

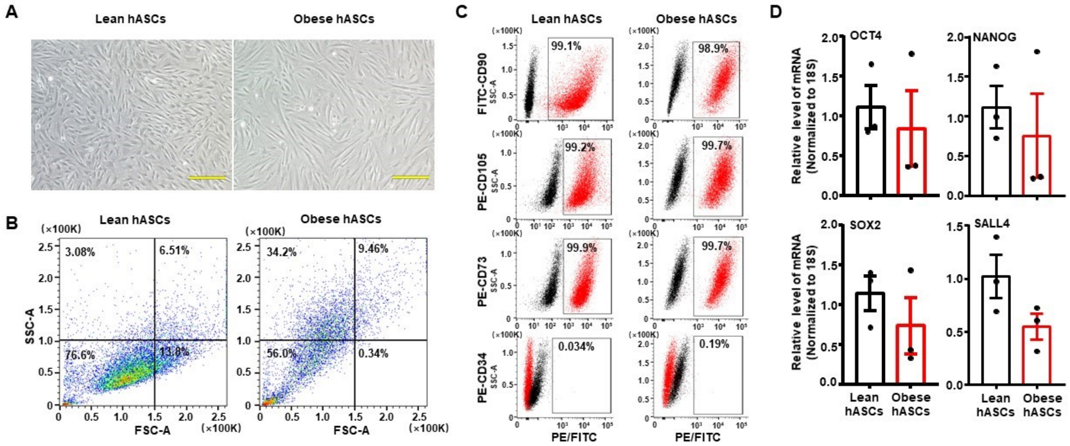

Figure S2. The characteristic of hASCs from lean donors and obese donors. Related to Figure 2. (A). Representative images of hASCs from lean donor and obese donor were determined using phase-contrast microscopy, Scale bars, 200 μm. (B). Representative images for analysing the cell size and particles in hASCs at Passage 6 using flow cytometry. Quadrant gating was applied to dot plots of FSC versus SSC for the indicated cells. The percentage of cells in each quadrant is shown as indicated. (C). Representative images for analysing immunophenotype of hASCs from lean donor and obese donor using flow cytometry after labelled with antibodies against the indicated antigens. The percentage of cells staining positive is indicated in the right corner of each box. The black dots indicate the isotype-matched monoclonal antibody control positive-staining cells. (D) Relative mRNA expression levels of multipotent stem cell markers, including OCT4, NANOG, SOX2, and SALL4 determined by quantitative RT-PCR. The relative expression of each gene was normalized against 18S rRNA. n=3 different donors. Data are presented as the means±SEM. Significant difference by unpaired two-tailed paired t test. FSC, forward scatter; SSC, scatter; FITC, fluorescein isothiocyanate; PE, phycoerythrin; CD73, 5'-nucleotidase ecto, NT5E; CD90, Thy-1 cell surface antigen, THY1; CD105, endoglin, ENG; OCT4, POU class 5 homeobox 1, POU5F1; NANOG, Nanog homeobox; SOX2, SRY-box transcription factor 2; SALL4, spalt like transcription factor 4.

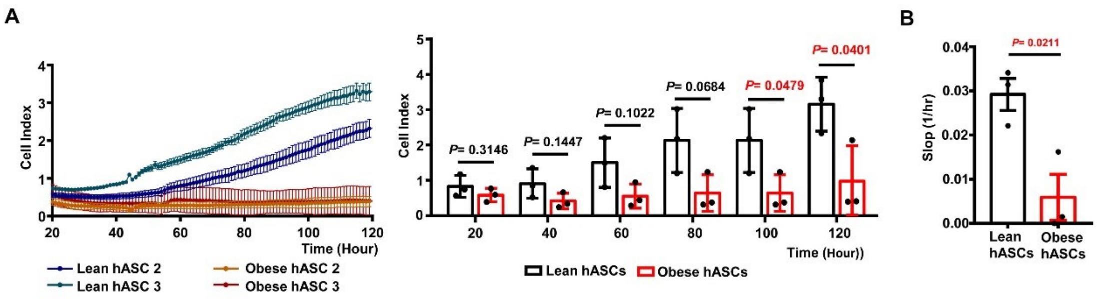

Figure S3. The growth curve of hASCs from lean donor and obese donor. Related to Figure 2. (A) Graph showing the cell growth curve of hASCs from lean donor and obese donor monitored and recorded by Realtime xCELLigence analysis (RTCA). Histogram showing the significant difference of the cell index of hASCs from two groups. Statistical significance compared to lean hASCs. Data are presented as the means±SD. (B) Histogram showing the significant difference of the hASCs proliferative capacity (represent as slop) from the 20th hour to the 120th hour from two groups. n =3 different donors per group. Statistical significance compared to lean hASCs. Data are presented as the means±SEM. $P > 0.05$ by unpaired two-tailed paired t test. hASCs, human adipose stem cells.

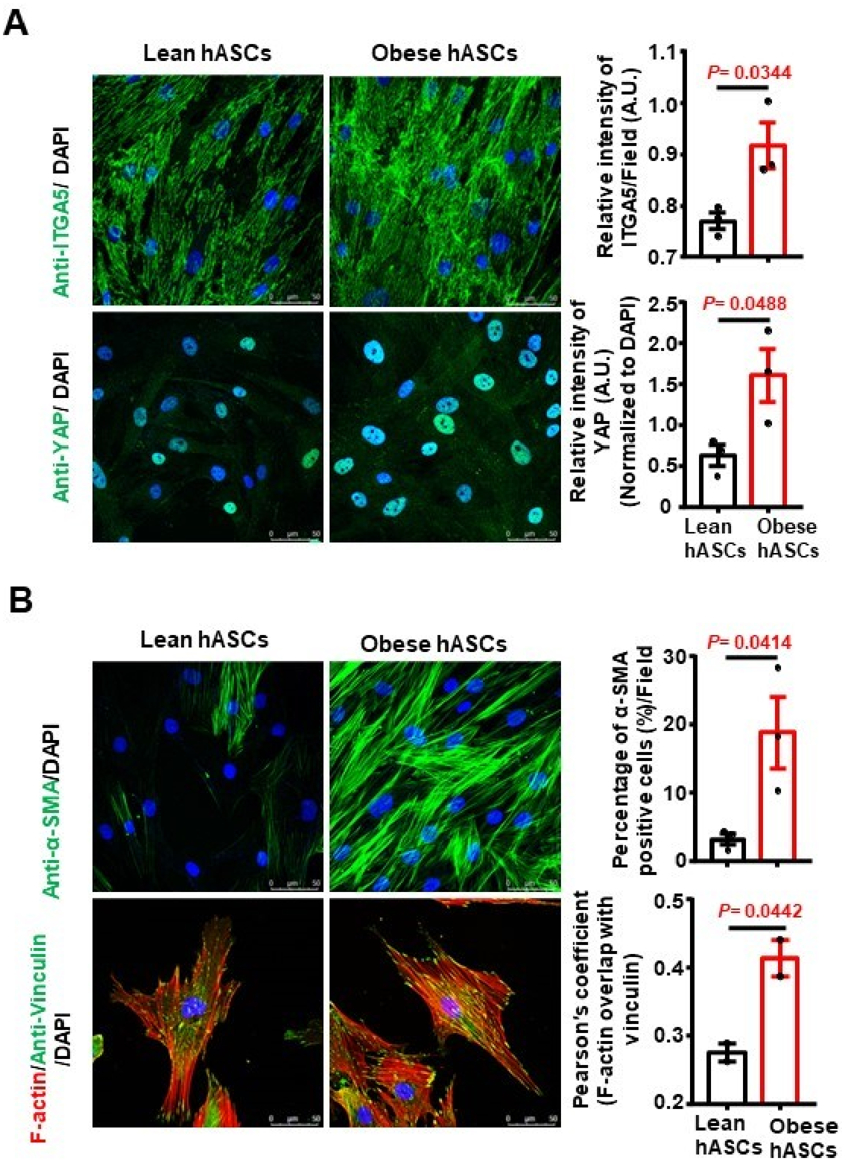

Figure S4. Response of hASCs to ECM in obese and lean adipose tissue. (A) Representative images of hASCs from lean and obese donors for immunostained with antibody against ITGA5 and YAP. Histogram showing the difference of the relative intensity of ITGA5 in the field and YAP normalized to DAPI each nuclear of cells. The number of fields for the expression of ITGA5 was 17-25 in each group respectively. The number of cells for the expression of YAP was 102-135 in each group respectively. n = 3 different donors. Data are presented as the means±SEM. Scale bars, 50 μm. (B) Representative images of hASC from obese and lean donors for the expression of α-SMA, vinculin and F-actin determined by immunofluorescence and Alexa Fluor 568 conjugated phalloidin. Histogram showing the difference of the positive cell percentage of α-SMA and co-localization between

F-actin signal and vinculin. The number of fields was 5-16 for the expression of α-SMA in each group respectively. n=3 different donors. The number of cells was 35-37 for co-localization between F-actin signal and vinculin in each group respectively. n = 2 different donors; Data are presented as the means±SEM. Significant difference by unpaired two-tailed paired t test. Scale bars, 50 μm. A.U. Arbitrary Unit. ITGA5, α5 integrin; YAP, Yes1 associated transcriptional regulator.

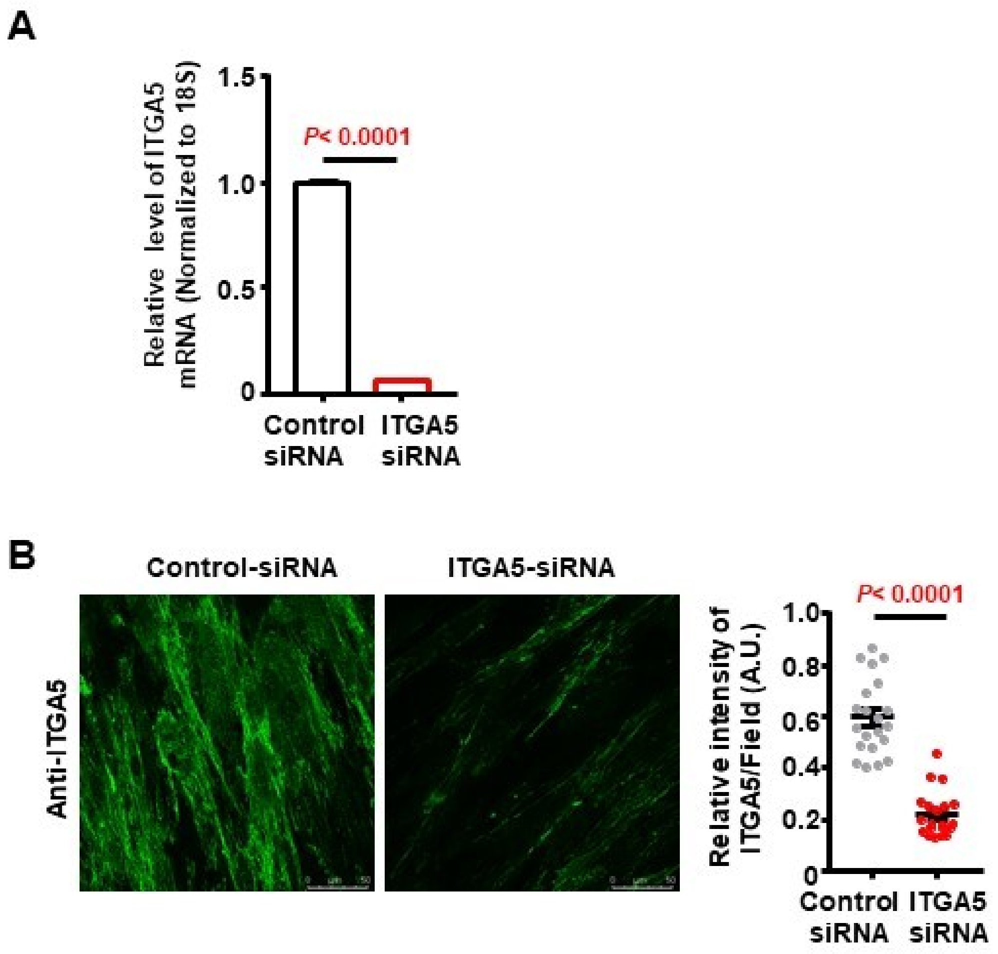

Figure S5. The expression of ITGA5 was knocked down in obese hASCs using ITGA5 siRNA and control siRNA. Related to Figure 3. (A) The relative mRNA levels of in obese hASCs using ITGA5 siRNA and control siRNA were determined by quantitative real time RT-PCR. The relative expression of each gene was normalized against 18S rRNA. Data are presented as the means±SEM. (B) Representative images of in obese hASCs using ITGA5 siRNA and control siRNA for the expression of ITGA5 determined by immunofluorescence. Histogram showing the difference of the the relative intensity of ITGA5 in the field. The number of fields was 20-21 for the expression of ITGA5 in each group respectively. Data are presented as the means±SEM. Significant difference by unpaired two-tailed paired t test. Scale bars, 50 μm. A.U. Arbitrary Unit. ITGA5, α5 integrin.

A

	GO ID	GOTerm	Corrected p-value	Associated gene count	GeneRatio
BP	GO:0030198	extracellular_matrix_organization	0.00000005	15	0.065217391
	GO:0085029	extracellular_matrix_assembly	0.00146009	3	0.013043478
	GO:0022617	extracellular_matrix_disassembly	0.03742011	3	0.008356546
	GO:0048251	elastic_fiber_assembly	0.00213044	2	0.008695652
	GO:0030199	collagen_fibril_organization	0.04828588	2	0.008695652
MF	GO:0001968	fibronectin_binding	0.00008754	4	0.017094017
	GO:0050431	transforming_growth_factor_beta_binding	0.00084277	3	0.012820513
	GO:0034713	type_I_transforming_growth_factor_beta_receptor_binding	0.00006176	3	0.012820513
	GO:0005201	extracellular_matrix_structural_constituent	0.00001403	9	0.038461538
	GO:0008191	metalloendopeptidase_inhibitor_activity	0.00000729	4	0.017094017
CC	GO:0062023	collagen-containing_extracellular_matrix	0.00000395	14	0.058823529
	GO:0071953	elastic_fiber	0.00031304	2	0.008403361
	GO:0031012	extracellular_matrix	0.00000005	19	0.079831933

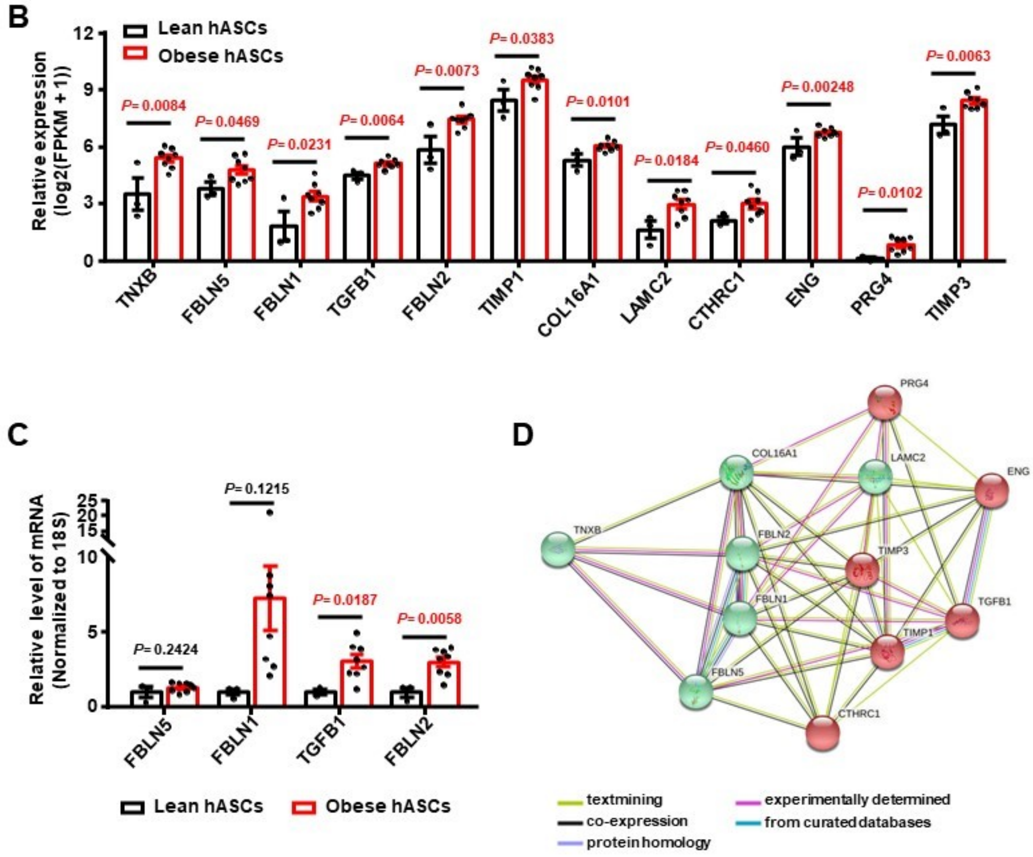

Figure S6. A functional gene annotation analysis of the identified upregulated differentially expressed genes between the lean hASCs and the obese hASCs involved in ECM remodelling. Related to Figure 5. (A) Selected GO categories enriched in differential expression genes between the lean hASCs and the obese hASCs which involved in with the extracellular matrix remodeling. (B) Histogram showing the relative expression of each gene was normalized log scaled FPKM (log2 (FPKM+1)). The differential expression genes involvement in three or more go categories associated with extracellular matrix remodelling were selected. Full results are reported in Table S5 in Additional file 2. Statistical significance

compared to lean hASCs. Significant difference by unpaired two-tailed paired t test. Data are presented as the means±SEM. Lean hASCs, n=3 different donors. Obese hASCs, n=8 different donors. Significant difference by unpaired two-tailed paired t test. Error bars represent SEM. FPKM, fragments per kilobase of transcript per million mapped reads. (C) Histogram showing the relative mRNA expression levels of interested gene determined by quantitative RT-PCR. The relative expression of each gene was normalized against 18S rRNA. n=3 different donors from lean hASCs, n=8 different donors from obese hASCs. Statistical significance compared to lean hASCs. Data are presented as the means±SEM. Significant difference by unpaired two-tailed paired t test. (D) Graph show the network view the associations between proteins from the regulated genes involved in extracellular matix remolded in lean hASCs and obese hASCs. The network node is protein. The edges represent the functional associations with differently colored lines. These proteins were clustered using KMEANS clustering algorithms. Every color of node corresponds to a cluster. TNXB, tenascin XB; FBLN5, fibulin 5; FBLN1, fibulin 1; TGFB1, transforming growth factor beta 1; FBLN2, fibulin 2; TIMP1, TIMP metallopeptidase inhibitor 1; COL16A1, type XVI collagen alpha 1 chain; LAMC2, laminin subunit gamma 2; CTHRC1, collagen triple helix repeat containing 1; ENG, endoglin; PRG4, proteoglycan 4; TIMP3, TIMP metallopeptidase inhibitor 3.

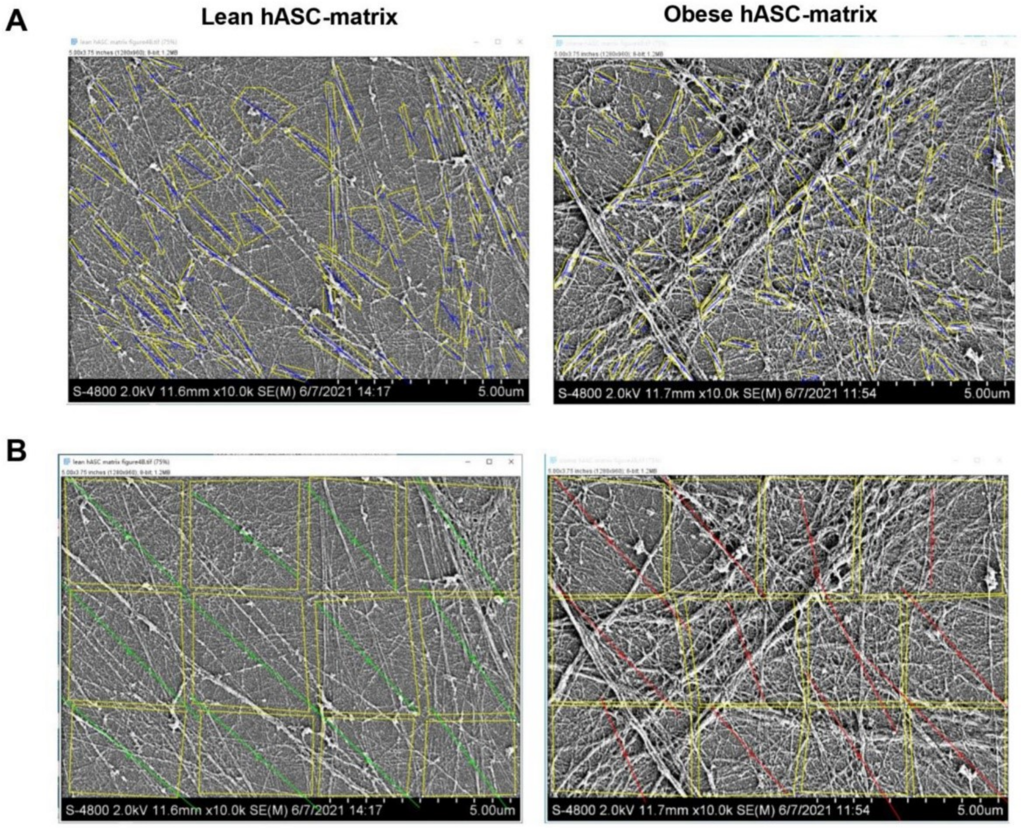

Figure S7. Illustration of fibres and ROI selection using the FibrilTool. Related to Figure 5B. (A) The polygon yellow area showing the selected fibres in the hASC-derived matrix from lean donor and obese donor. A blue line segment is drawn by FibrilTool according to the selected fibres. A log file collects the quantifications, notably including the orientation and anisotropy of the fiber arrays. (B) The polygon yellow area showing a given region of interest (ROI) in the hASC-derived matrix from lean donor and obese donor. The green or red line segment is drawn by FibrilTool according to ROI. A log file collects the quantifications, notably including the orientation and anisotropy of the ROI. hASC-matrix, hASC-derived matrix.

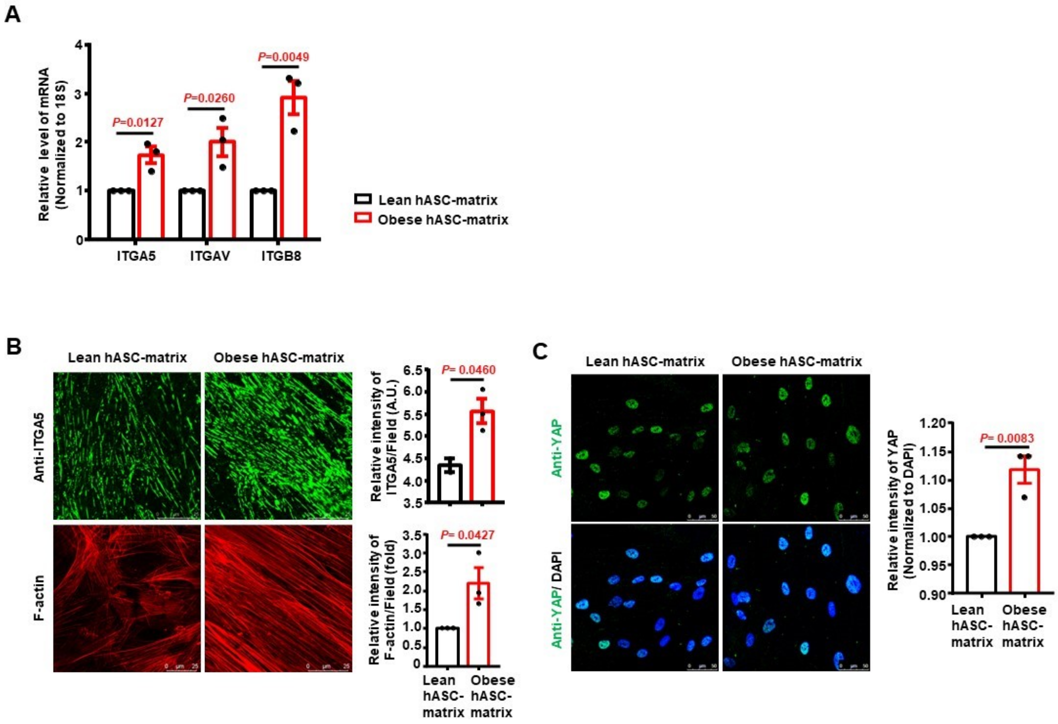

Figure S8. The response of lean hASCs to different hASC-derived matrix were determined. (A) The relative mRNA levels of ITGA5, ITGAV and ITGB8 in the lean hASCs cultured on lean hASC-derived matrix and obese hASC-derived matrix were determined by quantitative RT-PCR. The relative expression of each gene was normalized against 18S rRNA. n=3 independent experiments from lean hASC-derived matrix group, n=3 different donors from obese hASC-derived matrix group. (B) Representative images of lean hASC cultured on different hASC-derived matrix for immunostained with antibody against ITGA5 and the expression of F-actin determined by Alexa Fluor 568 conjugated phalloidin. Histogram showing the difference of the relative intensity of ITGA5 in each field from the lean hASC cultured on different hASC-derived matrix. n=2 independent experiments from lean hASC-derived matrix group; n=3 different donors from obese hASC-derived matrix. The number of fields was 15-24 in each group respectively. Histogram showing the difference of the relative intensity of F-actin in each field from the lean hASC cultured on different hASC-derived matrix. n=3 donor from lean hASC-derived matrix group; The number of fields was 18-23 in each group respectively. n=3 different donors from obese hASC-derived matrix group. Scale bars, 25 μm. (C) Representative images of lean hASC cultured on different hASC-derived matrix for immunostained with antibody against YAP. Histogram showing the difference of the relative intensity of YAP normalized to DAPI each nuclear of cells. The

number of cells was 218-457 in each group respectively. n = 3 donor from lean hASC-derived matri; n=3 different donors from obese hASC-derived matrix. Scale bars, 50 μm. Data are presented as the means±SEM. Significant difference by unpaired two-tailed paired t test. A.U. Arbitrary Unit. Fold represents the relative intensity was normalized against lean hASCs cultured on lean hASC-derived matrix from each independent experiment. hASC-matrix, hASC-derived matrix; ITGA5, α5 integrin; YAP, Yes1 associated transcriptional regulator.

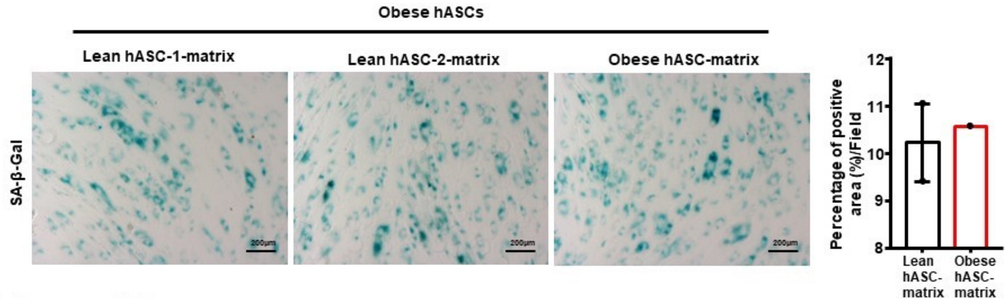

Figure S9. SA-β-Gal staining of obese hASCs cultured on different lean hASC-derived matrix. Representative images SA-β-Gal staining of obese hASCs cultured on lean hASC-derived matrix derived from different donors and obese hASC-derived matrix. Scale bars, 200 μm. Histogram showing the difference of the positive area percentage of SA-β-Gal from different lean hASC-derived matrix groups. The number of fields was 12-16 in each group respectively. n=2 independent experiments from lean hASC-derived matrix, n=1 different donors from obese hASC-derived matrix. Data are presented as the means±SEM. Significant difference by unpaired two-tailed paired t test. hASC-matrix, hASC-derived matrix.

.

Table S1. List of antibodies used in the study.

Primary Antibodies

**Primary Antibody**	**Dilution**	**Company**	**Clonality**	**Cat. No.**
Rabbit anti-Ki-67	1:150	ZSGB-BIO	monoclonal	ZA-0502
Rabbit anti-type I Collagen	1:100	ZSGB-BIO	monoclonal	ZA-0616
Mouse anti-Fibronectin	1:500	Merck Millipore	monoclonal	MAB88916-C
Mouse anti-SMA	1:100	ZSGB-BIO	monoclonal	ZM-0003
Mouse anti-CD31	1:3200	Cell Signaling Technology	monoclonal	3528
Rabbit anti-TGF-beta-1	1:50	proteintech	polyclonal	21898-1-AP
Rabbit anti-integrin alpha5	1:500	Abcam	monoclonal	ab150361
Mouse anti-YAP1	1:500	Merck Millipore	monoclonal	MABC203
NeutraKine® TGF beta 1 Monoclonal antibody	10μg/ml	proteintech	monoclonal	69012-1-Ig
Mouse anti-CD90	1:100	BD Biosciences	monoclonal	555595
Mouse anti-CD73	1:100	BD Biosciences	monoclonal	550257
Mouse anti-CD105	1:100	BD Biosciences	monoclonal	560839
Mouse anti-CD34	1:100	BD Biosciences	monoclonal	550761
PE Mouse IgG1, κ Isotype Control	1:100	BD Biosciences		555749
FITC Mouse IgG1, κ Isotype Control	1:100	BD Biosciences		555748

Secondary Antibodies

**Secondary Antibody**	**Dilution**	**Company**	**Clonality**	**Cat. No.**
Alexa Fluor® 488 Conjugate (Goat Anti-Mouse IgG)	1:500	Cell Signaling Technology		4408S
Alexa Fluor® 488 Conjugate (Goat Anti-Rabbit IgG)	1:500	Cell Signaling Technology		4412S
Alexa Fluor™ 594 (Goat anti-Rabbit IgG)	1:500	Invitrogen		A11012
Alexa Fluor™ 594 (Goat anti-Mouse IgG)	1:500	Invitrogen		A11005

Table S2. Summary of primer sequences used in the study.

Accession number	Name	5'-sequence-3'
NM_000877.4	IL1R1	F: ACCAGCCACTAAGGAGAAAC<br>R: ACAGGAGGCACCTAAAGAAC
NM_000598.5	IGFB3	F: CTAGTGAGTCGGAGGAAGACC<br>R: ACTCGTAGTCAACTTTGTAGCG
NM_000660.7	TGFB1	F: CAGCAACAATTCCTGGCGATAC<br>R: GCTAAGGCGAAAGCCCTCAAT
NM_001024847.2	TGFBR2	F: CCCAGGTAAGGATAGCAGAT<br>R: CAGGTAGGCAGTGGAAAGAG
NM_000389.5	CDKN1A	F: CCTGTCACTGTCTTGTACCCTTGT<br>R: GCTTCCTGTGGGCGGATTAG
NM_000077.5	CDKN2A	F: TCATGTGGGCATTTCTTGCG<br>R: GCTTTGGTTCTGCCATTTGCT
NM_004936.4	CDKN2B	F: TGGACCTGGTGGCTACGAAT<br>R: GCCGTCACCCAAGTGCTAAT
NM_000546.6	TP53	F: TACCACCATCCACTACAACTACAT<br>R: CCAGGACAGGCACAAACACG
NM_002205.4	ITGA5	F: TGTGACTACTTTGCCGTGAACC<br>R: CGGAGATGAGGGACTGTAAACC
NM_001144999.3	ITGAV	F: AAGTAAGCCCAGTTGTATCTC<br>R: TTCTTCAGTCTCAGGGTTCT
NM_002214.3	ITGB8	F: TAGTTACATTCTTGATTGGGTTGC<br>R: CGTCGGTAGGTGACTGCTCTT
NM_112957.3	OCT4	F: ATTCAGCCAAACGACCATCT<br>R: TTGTTGTCAGCTTCCTCCAC
NM_003106.4	SOX2	F: TCCATGACCAGCTCGCAGAC<br>R: TGGGAGGAAGAGGTAACCACAGG
NM_001318031.2	SALL4	F: CCCTTCAGTGAATGTGGACC<br>R: CTTGGAGACAGTGGCGTTAT
NM_001297698.2	NANOG	F: CCTATGCCTGTGATTTGTGG<br>R: TTTGCCTTTGGGACTGGTGG
NM_000404.4	GLB1	F: TCCACAATCAAGACCGAAGC<br>R: AATTGGTCCCACCTATAAACAT
NR_003286.4	18S	F: GTAACCCGTTGAACCCCATT<br>R: CCATCCAATCGGTAGTAGCG
