# Supplementary material for: Blockade of TGF-β signalling alleviates human adipose stem cell senescence induced by native ECM in obesity visceral white adipose tissue
Source: Stem Cell Res Ther. 2023 Oct 8;14:291. doi: 10.1186/s13287-023-03525-y (PMC10561428; doi:10.1186/s13287-023-03525-y)
Supplement: Supplementary file 2 — Additional file 2. Supplementary Table S3, S4 and S5 of Blockade of TGF-β signalling alleviates human adipose stem cell senescence induced by native ECM in obesity visceral white adipose tissue. [file 13287_2023_3525_MOESM2_ESM.pdf]

Table S3. The differentially expressed genes in the obese hASCs and lean hASCs

Gene_Name	log2FC	Fold_Change	p_value	q_value	Obese_FPKM	Control_FPKM	obese 1	obese 2	obese 3	obese 4	obese 5	obese 6	obese 7	obese 8	lean 1	lean 2	lean 3
PI16	3.559	11.787	0.012	0.239	5.639	2.080	138.439	23.409	304.697	39.476	24.978	39.152	9.410	82.233	0.088	17.874	2.681
STMN2	2.875	7.334	0.034	0.323	4.083	1.208	16.871	40.792	0.513	18.395	6.112	18.479	73.563	28.979	0.000	7.270	0.490
ALDH1A3	2.670	6.363	0.024	0.287	5.250	2.580	61.693	17.717	19.469	35.869	51.657	39.400	73.901	30.122	0.463	52.113	1.752
IGFBP3	2.499	5.655	0.024	0.289	8.370	5.871	186.949	197.847	405.252	371.571	385.995	442.859	296.844	496.046	5.621	168.253	177.850
TFPI2	2.450	5.464	0.001	0.123	6.325	3.875	111.249	100.689	86.143	147.941	71.640	88.116	79.259	21.127	9.132	20.006	13.828
ISLR	2.449	5.460	0.015	0.253	7.220	4.771	196.318	73.314	208.441	94.367	109.992	157.290	264.472	177.747	4.052	108.920	35.671
CRLF1	2.404	5.293	0.021	0.279	5.829	3.424	79.267	80.971	112.505	33.380	12.341	33.104	146.929	61.946	2.233	32.731	10.347
TOMM6	2.355	5.117	0.009	0.223	4.878	2.523	35.768	45.555	49.800	53.878	5.304	15.022	30.762	35.509	11.541	2.300	3.586
DKK1	2.276	4.844	0.016	0.258	5.631	3.355	90.036	94.053	143.791	35.330	15.583	36.632	53.749	22.419	2.539	16.849	15.953
CYGB	2.217	4.650	0.003	0.160	3.967	1.749	16.110	30.173	19.038	11.967	7.642	7.360	15.989	19.973	0.336	5.078	3.680
KCTD12	2.092	4.263	0.032	0.317	4.596	2.504	58.418	18.412	52.006	20.579	17.380	16.744	16.535	14.510	0.181	22.193	5.663
CDKN2B	2.015	4.043	0.004	0.196	4.636	2.621	23.985	14.637	34.949	23.294	15.020	21.691	21.752	50.821	1.327	12.525	6.394
UNC5B	1.956	3.880	0.045	0.356	4.699	2.743	12.466	23.374	26.341	15.971	11.039	42.600	49.486	50.315	0.358	23.386	8.058
CFH	1.941	3.839	0.003	0.176	4.248	2.307	17.153	8.083	21.597	47.252	23.746	16.896	7.806	23.170	3.394	5.057	3.551
SERPINE2	1.895	3.720	0.009	0.223	8.584	6.689	455.147	308.395	508.143	261.947	261.722	304.506	537.716	575.752	33.839	301.187	103.312
TNXB	1.894	3.716	0.008	0.220	5.399	3.505	48.785	29.123	47.960	59.639	65.679	22.072	33.508	41.454	6.941	34.929	4.129
SLC22A3	1.860	3.631	0.035	0.324	2.755	0.895	0.616	6.715	2.323	22.239	2.810	7.220	12.707	9.463	0.433	1.511	0.788
HTRA3	1.852	3.610	0.017	0.262	3.042	1.190	6.865	7.767	17.529	5.102	4.025	3.238	17.847	5.778	0.444	5.397	0.287
GALNT12	1.834	3.566	0.008	0.220	3.142	1.308	11.376	2.561	3.362	13.571	9.966	10.241	7.005	12.331	0.240	3.470	1.736
LXN	1.819	3.529	0.004	0.187	5.826	4.007	53.344	56.842	27.046	122.723	49.332	87.008	60.087	35.290	14.800	27.029	8.376
GFPT2	1.803	3.489	0.024	0.289	3.759	1.956	16.681	7.956	6.316	16.615	8.212	22.220	25.710	8.699	0.172	9.661	3.679
SAMD11	1.774	3.420	0.001	0.124	2.883	1.109	6.356	5.076	3.064	6.001	4.672	6.989	13.420	9.556	0.449	2.054	1.268
BST2	1.753	3.370	0.013	0.243	5.825	4.072	21.122	195.225	52.688	50.106	61.450	64.686	53.631	38.860	11.154	27.025	12.966
IL1R1	1.686	3.217	0.014	0.249	5.621	3.935	46.871	37.265	51.384	93.204	60.223	37.231	38.246	40.420	6.414	49.803	8.505
ANK3	1.644	3.126	0.029	0.308	2.889	1.245	5.312	19.737	8.760	14.911	8.574	2.266	1.631	4.430	0.757	1.946	1.573
BMP6	1.629	3.092	0.033	0.323	3.439	1.810	20.792	3.665	16.901	6.421	8.813	5.723	8.691	21.145	0.484	7.967	2.242
FBLN2	1.608	3.048	0.007	0.214	7.433	5.825	168.770	106.374	300.557	179.154	129.582	169.843	197.337	180.521	27.711	144.261	42.699
SGCG	1.606	3.044	0.049	0.362	3.764	2.158	9.487	10.856	17.806	15.730	4.340	23.282	11.648	17.052	0.513	16.804	2.297
FBLN1	1.590	3.010	0.023	0.286	3.408	1.818	8.623	10.641	23.505	10.134	4.652	5.716	7.576	15.167	1.171	9.035	1.011
NDN	1.584	2.998	0.049	0.362	3.725	2.141	9.131	6.733	5.648	13.444	11.723	13.430	24.180	25.840	0.287	3.814	12.840
AADAC	1.576	2.981	0.010	0.226	1.864	0.288	4.859	2.339	5.246	3.773	1.724	0.306	1.768	4.361	0.000	0.819	0.000
ABCA9	1.569	2.967	0.037	0.332	3.747	2.178	5.754	11.310	9.213	26.376	13.499	15.281	12.502	13.253	0.506	14.780	2.899
LACC1	1.567	2.962	0.026	0.299	4.601	3.034	35.594	13.701	73.918	29.000	22.471	12.417	13.881	20.179	3.058	15.043	7.438
SEMA3C	1.564	2.957	0.018	0.268	6.779	5.215	110.044	107.338	153.662	111.330	73.478	87.775	125.684	119.777	13.842	125.784	26.209
EPDR1	1.555	2.939	0.048	0.361	4.621	3.066	24.853	14.294	20.626	16.175	20.221	24.015	28.810	56.920	3.252	34.976	2.839
ZFPM2	1.546	2.921	0.002	0.157	2.377	0.831	5.488	4.468	4.672	3.351	1.805	4.272	3.894	7.369	0.018	1.656	1.081
MMP16	1.540	2.908	0.015	0.255	2.824	1.284	8.000	11.254	8.606	8.959	1.261	4.687	7.041	4.791	0.449	3.068	1.448
HMOX1	1.538	2.904	0.018	0.269	4.407	2.869	15.949	16.439	9.126	19.531	25.743	33.991	47.549	13.668	3.738	15.147	4.092
ENC1	1.538	2.903	0.034	0.323	4.384	2.846	26.140	24.904	10.290	18.861	15.616	22.072	15.550	35.121	0.902	12.549	13.435
FZD8	1.533	2.894	0.001	0.107	2.661	1.128	4.055	5.612	6.820	7.032	3.220	5.377	5.968	5.501	0.248	2.496	1.391
IFI30	1.519	2.867	0.001	0.123	3.641	2.122	18.724	12.861	5.579	12.092	12.398	15.740	12.606	7.173	2.937	3.020	4.209
HOXA5	1.482	2.794	0.017	0.262	4.446	2.964	30.560	11.892	20.178	10.395	15.565	24.315	41.512	28.153	3.509	15.835	5.257
CRABP2	1.475	2.781	0.048	0.361	5.023	3.547	103.710	17.199	59.518	27.493	11.009	23.245	45.582	26.986	14.304	15.626	5.279
ABCA8	1.449	2.730	0.019	0.270	4.227	2.778	11.491	9.603	9.714	20.231	22.464	27.684	29.794	23.213	2.442	13.821	5.325
CFI	1.448	2.728	0.041	0.343	2.904	1.456	5.559	3.194	2.072	10.176	12.061	10.814	4.872	10.491	0.376	5.956	1.157
PLXDC2	1.436	2.705	0.021	0.278	2.725	1.290	2.806	3.042	4.548	5.657	5.179	7.394	8.287	12.368	0.006	3.521	2.213
MYCT1	1.378	2.598	0.036	0.327	3.351	1.973	20.629	12.811	11.779	9.542	5.008	7.125	5.981	7.571	0.339	5.545	5.911
PKDCC	1.372	2.588	0.004	0.192	3.093	1.721	4.856	10.952	4.738	11.653	14.110	6.827	5.091	6.660	1.304	3.200	2.702

CDH2	1.357	2.561	0.030	0.310	7.336	5.980	177.264	154.421	159.676	109.109	90.680	223.635	197.376	231.513	20.839	142.198	79.402
P4HA3	1.349	2.547	0.003	0.176	2.830	1.481	6.424	7.261	7.685	7.536	2.748	7.312	7.350	4.525	0.622	2.932	2.412
PID1	1.345	2.541	0.046	0.357	2.777	1.432	7.236	2.556	10.911	3.401	2.800	8.485	9.692	7.255	0.170	5.753	1.487
MRAS	1.339	2.530	0.047	0.359	2.720	1.381	3.289	1.976	9.596	5.748	2.128	12.481	15.919	4.460	0.905	1.808	2.302
LAMC2	1.332	2.517	0.018	0.269	2.985	1.653	9.442	2.724	5.316	7.529	11.869	3.789	11.923	8.241	0.730	3.885	2.680
ENOX1	1.320	2.497	0.002	0.159	1.814	0.494	2.193	2.473	4.354	5.406	2.158	1.625	1.945	1.512	0.046	0.818	0.469
HOXB9	1.308	2.476	0.017	0.264	3.551	2.243	9.997	9.704	10.435	5.946	6.941	19.685	18.751	10.763	1.694	8.824	3.012
ID1	1.284	2.435	0.034	0.323	5.764	4.479	72.487	58.762	55.291	30.393	35.251	23.382	114.515	94.803	28.496	26.947	12.467
SESN3	1.282	2.432	0.032	0.317	3.802	2.520	26.877	9.909	9.482	12.114	8.299	16.798	8.490	20.840	1.326	8.074	7.937
KLF4	1.275	2.419	0.007	0.214	3.677	2.403	14.424	13.627	17.137	13.513	8.316	9.536	11.481	8.869	1.630	8.863	4.702
CFHR1	1.271	2.414	0.001	0.106	1.402	0.131	1.945	0.706	2.304	1.956	2.961	1.404	0.865	1.725	0.236	0.000	0.061
IL33	1.260	2.395	0.017	0.264	2.113	0.852	5.350	1.526	1.306	6.611	3.794	2.447	5.904	2.808	0.226	1.737	0.755
CYP1B1	1.259	2.392	0.007	0.214	6.341	5.082	48.595	78.912	158.097	84.679	68.754	69.181	109.186	62.856	32.463	49.757	21.884
TIMP3	1.255	2.387	0.006	0.214	8.423	7.168	558.899	297.441	259.842	464.162	252.603	359.500	278.207	371.574	110.373	271.046	97.183
DACT1	1.251	2.380	0.013	0.243	4.859	3.608	26.162	26.333	17.054	24.456	21.401	45.309	38.451	34.971	5.078	21.273	12.386
IGFBP6	1.235	2.354	0.000	0.100	7.924	6.688	292.217	193.677	201.235	216.266	242.159	323.098	213.722	283.505	91.174	143.807	81.166
SH3BP5	1.212	2.317	0.036	0.327	3.997	2.784	18.332	7.386	13.459	13.329	12.438	16.361	16.287	30.108	2.194	15.216	5.310
FAM20A	1.205	2.305	0.009	0.223	1.686	0.481	2.866	0.598	2.835	2.397	2.130	2.171	2.494	3.123	0.000	1.643	0.029
PTGES	1.204	2.303	0.007	0.214	1.817	0.614	1.543	4.231	1.973	1.834	1.253	2.115	4.889	4.133	0.233	1.081	0.395
KIAA1324L	1.203	2.303	0.034	0.324	2.322	1.119	4.112	3.177	2.698	1.304	7.691	4.026	3.985	8.859	0.106	2.419	1.707
FLRT2	1.183	2.271	0.035	0.324	2.138	0.955	2.891	1.708	1.466	4.278	4.931	4.950	4.893	3.928	0.019	3.690	0.523
ABCA6	1.176	2.260	0.021	0.277	1.956	0.780	1.897	3.010	1.597	6.467	3.924	3.170	2.654	2.041	0.112	2.646	0.249
ELK3	1.168	2.246	0.001	0.123	3.998	2.830	17.929	9.876	17.834	16.032	15.447	14.770	17.947	12.085	4.443	5.150	9.747
GRIK2	1.143	2.209	0.034	0.323	2.584	1.441	4.567	4.376	5.807	6.610	4.516	3.761	6.523	4.460	0.052	5.853	1.776
MN1	1.138	2.201	0.005	0.198	3.878	2.740	11.395	14.176	15.043	14.399	12.768	11.976	18.107	12.752	2.986	12.110	4.705
XAF1	1.136	2.198	0.003	0.160	3.496	2.360	6.063	18.184	10.419	9.654	7.521	11.706	11.914	10.384	3.414	5.698	3.575
FSTL3	1.136	2.197	0.023	0.287	4.838	3.702	23.763	26.693	40.728	18.695	22.403	23.864	30.581	42.206	24.118	4.837	14.041
SCG2	1.132	2.192	0.030	0.309	1.723	0.591	0.650	3.134	2.586	7.589	1.053	1.860	2.930	1.913	0.258	0.853	0.467
ADGRG6	1.131	2.191	0.016	0.261	2.978	1.847	8.245	9.844	4.515	10.982	7.177	7.852	5.783	3.566	0.896	3.538	4.405
RCN3	1.125	2.180	0.000	0.042	6.620	5.495	130.826	97.572	115.099	88.635	88.300	96.932	96.960	74.552	45.491	41.923	44.982
ADAM12	1.119	2.172	0.015	0.255	5.409	4.289	31.277	58.758	19.523	59.619	40.365	55.866	65.851	27.111	16.602	19.594	19.629
CCL26	1.104	2.149	0.011	0.236	1.895	0.791	2.223	2.349	1.133	3.683	1.311	4.368	3.773	4.730	0.485	0.494	1.335
TIMP1	1.100	2.144	0.038	0.334	9.537	8.437	1074.618	352.076	1152.687	887.673	569.813	847.838	610.720	803.327	184.346	699.666	319.492
SH3D19	1.097	2.139	0.011	0.236	4.383	3.286	18.270	15.128	11.493	25.789	25.482	23.544	23.737	20.477	4.353	15.062	9.800
RASSF8	1.090	2.129	0.007	0.214	3.054	1.963	2.952	8.796	6.806	8.656	6.915	9.228	10.059	7.652	4.608	2.162	2.344
SORCS2	1.086	2.122	0.013	0.243	1.647	0.562	2.435	0.727	2.074	1.113	1.811	3.170	4.391	2.806	0.024	0.909	0.645
MFAP3L	1.062	2.087	0.014	0.249	3.535	2.473	15.136	15.642	9.641	11.324	6.075	8.638	8.858	12.764	2.279	8.393	4.560
KCND2	1.061	2.087	0.008	0.217	2.038	0.977	2.283	1.342	2.536	4.680	5.789	2.675	3.354	3.833	0.597	1.574	0.854
RNF6	1.059	2.084	0.020	0.274	3.384	2.324	7.722	10.147	10.475	12.586	4.926	15.502	15.175	4.874	2.734	4.152	5.532
MED21	1.047	2.066	0.002	0.157	2.935	1.888	6.576	8.152	7.083	7.481	7.303	6.735	3.224	8.063	2.405	3.944	2.011
CMTM3	1.017	2.024	0.001	0.106	3.272	2.254	6.784	11.012	12.264	9.680	8.554	6.995	6.401	9.100	3.398	4.823	3.240
CLEC11A	0.998	1.998	0.005	0.196	4.961	3.963	34.761	21.744	50.011	28.076	31.605	23.280	30.508	28.490	9.543	19.029	16.956
EN1	0.998	1.997	0.043	0.348	1.358	0.360	2.109	0.677	0.028	2.586	3.548	2.437	1.561	1.419	0.016	0.614	0.288
MEX3B	0.997	1.996	0.043	0.349	4.499	3.502	22.836	25.519	37.786	21.085	20.823	8.860	14.820	36.056	7.894	16.865	8.144
ARHGAP20	0.997	1.995	0.028	0.305	1.949	0.952	1.973	2.603	1.452	3.740	6.626	3.115	2.210	2.932	0.134	2.222	0.982
NCOA7	0.993	1.991	0.023	0.284	4.342	3.349	13.693	23.404	23.815	20.497	15.710	16.912	19.335	23.641	6.426	21.564	5.316
PCDHGC4	0.990	1.986	0.050	0.364	1.172	0.182	1.636	1.453	0.052	0.166	2.446	1.095	1.430	3.774	0.195	0.099	0.112
GPX7	0.982	1.975	0.010	0.223	3.255	2.273	5.510	7.606	4.861	6.704	10.007	11.622	12.781	13.243	4.182	3.867	3.481
FBLN5	0.979	1.971	0.047	0.359	4.768	3.789	35.615	17.324	48.737	18.500	29.248	15.135	19.349	46.050	10.692	20.815	9.358
C2orf74	0.977	1.969	0.019	0.271	2.980	2.002	4.389	9.275	9.801	5.637	4.538	12.605	4.258	8.529	3.908	3.442	1.949
PRPH2	0.974	1.964	0.020	0.274	1.709	0.735	1.978	1.317	2.844	3.083	1.880	2.740	4.280	1.113	1.871	0.391	0.154
KCNT2	0.972	1.962	0.010	0.231	1.367	0.394	1.867	0.568	0.763	2.942	2.382	2.081	1.037	1.950	0.023	0.533	0.447
SAMD9	0.972	1.961	0.006	0.214	3.781	2.810	15.109	21.784	16.106	13.202	10.658	10.245	11.392	7.823	5.500	8.108	4.822

FLRT3	0.634	1.552	0.000	0.089	0.891	0.257	1.124	0.542	0.688	0.827	0.994	0.747	1.163	0.844	0.093	0.257	0.242
GBP4	0.958	1.943	0.032	0.317	1.241	0.283	4.036	2.863	1.174	0.425	1.621	0.933	0.802	0.774	0.089	0.501	0.102
UST	0.951	1.933	0.041	0.344	3.662	2.711	18.398	10.142	17.715	7.693	10.515	12.408	9.745	10.283	2.873	13.357	4.045
PCSK9	0.949	1.931	0.000	0.105	1.180	0.231	0.915	1.611	2.061	1.211	0.896	1.296	0.963	1.408	0.000	0.524	0.061
HEG1	0.940	1.919	0.021	0.278	7.260	6.320	198.226	135.550	156.971	112.004	172.463	118.480	149.381	200.101	56.422	160.256	54.047
SYNDIG1	0.936	1.913	0.010	0.228	1.658	0.722	1.539	1.690	1.727	2.239	1.285	3.218	3.523	2.749	0.032	1.246	0.937
SH3KBP1	0.934	1.911	0.001	0.127	4.432	3.498	25.883	24.425	17.129	22.963	16.073	23.180	23.256	14.862	8.422	13.088	9.863
ID3	0.934	1.910	0.023	0.285	6.226	5.292	77.211	82.447	73.888	61.879	55.071	40.787	114.055	117.855	51.998	40.895	26.085
PMEPA1	0.926	1.899	0.025	0.292	2.771	1.845	4.547	5.574	7.263	7.788	3.506	6.233	6.794	6.004	0.891	5.563	2.738
SDC1	0.922	1.895	0.046	0.356	2.166	1.244	4.940	5.515	1.127	1.973	2.762	4.233	3.303	6.950	0.978	1.041	2.292
S100A13	0.915	1.885	0.002	0.157	4.226	3.311	24.562	14.313	19.014	22.771	21.522	14.799	14.552	13.572	10.339	7.121	9.613
OR2W3	0.911	1.880	0.049	0.362	1.138	0.228	3.011	2.430	2.417	1.620	0.674	0.501	0.391	0.280	0.242	0.212	0.067
SNAI1	0.909	1.878	0.009	0.223	2.978	2.069	6.206	6.197	5.044	6.480	5.647	7.774	11.910	7.436	1.917	5.452	2.927
PROCR	0.904	1.872	0.007	0.214	5.106	4.201	38.335	40.895	34.727	48.478	33.057	27.612	24.541	26.241	23.198	20.160	11.158
AKR1C1	0.899	1.865	0.038	0.335	1.697	0.798	5.748	1.516	3.287	2.825	1.240	1.328	2.974	1.114	0.895	1.106	0.316
PRRX2	0.898	1.864	0.027	0.302	3.805	2.906	17.780	9.463	21.600	16.249	9.177	6.409	16.397	13.477	6.522	8.340	4.998
TPST1	0.891	1.854	0.011	0.234	3.934	3.044	12.952	9.719	21.209	15.397	12.263	13.245	20.376	12.558	5.703	11.868	5.498
ANKRD11	0.889	1.852	0.000	0.028	4.186	3.296	16.623	20.307	16.739	16.237	17.058	16.062	17.684	17.205	7.605	8.206	10.973
SPATA18	0.884	1.846	0.045	0.356	2.195	1.311	4.895	2.317	2.800	4.882	1.745	4.863	3.828	4.690	0.358	3.545	1.473
CCDC137	0.884	1.845	0.018	0.266	2.558	1.674	5.442	2.086	7.507	5.425	4.516	5.127	4.489	6.171	1.660	1.528	3.834
OAF	0.875	1.833	0.023	0.285	3.651	2.776	15.068	10.304	9.912	8.890	11.162	12.780	16.874	9.547	2.716	10.475	6.539
CTHRC1	0.873	1.831	0.046	0.357	3.005	2.132	7.858	6.267	3.488	14.706	5.038	11.585	8.117	4.482	3.222	2.619	4.512
PDGFRA	0.871	1.829	0.013	0.246	6.118	5.247	83.585	50.376	47.015	77.202	68.386	85.554	104.559	51.490	37.986	49.338	26.932
CHSY3	0.868	1.825	0.043	0.348	2.161	1.293	3.140	4.434	1.806	3.796	3.309	4.335	4.211	3.416	0.241	1.397	3.950
CDK14	0.864	1.821	0.013	0.245	3.652	2.788	9.094	13.372	6.187	13.614	11.667	13.217	14.718	13.484	3.839	8.863	5.902
CLMP	0.862	1.818	0.016	0.261	5.554	4.692	68.212	42.180	26.694	46.144	44.218	50.785	54.899	45.574	16.016	37.437	25.408
PGD	0.854	1.808	0.012	0.236	5.237	4.383	35.213	44.735	49.447	37.454	48.001	30.775	33.885	22.416	27.880	20.503	13.612
ADD3	0.854	1.808	0.009	0.223	5.303	4.449	31.644	41.212	50.139	54.844	40.490	38.421	27.951	30.688	18.972	31.198	15.205
NMNAT2	0.851	1.804	0.008	0.216	1.926	1.075	3.056	2.064	1.480	3.259	2.454	3.467	5.277	2.420	1.173	1.372	0.812
RGS2	0.849	1.802	0.002	0.157	1.833	0.984	3.082	4.077	1.289	2.968	2.594	2.396	2.595	2.151	0.931	1.025	0.979
MARCH3	0.844	1.795	0.049	0.362	2.469	1.625	4.692	2.485	4.737	6.318	4.515	5.891	4.000	4.575	0.498	4.862	2.342
TP53I3	0.844	1.795	0.013	0.243	3.911	3.067	17.885	11.572	10.135	12.165	12.759	21.810	11.104	18.838	4.850	9.483	8.606
CLCN4	0.842	1.793	0.048	0.361	1.788	0.946	2.531	2.389	3.558	1.605	1.579	1.480	3.214	4.288	0.225	2.768	0.548
DPYSL3	0.833	1.781	0.032	0.316	5.428	4.595	34.805	51.358	67.797	40.607	33.500	39.648	42.660	34.899	14.314	44.388	19.308
DOCK11	0.833	1.781	0.042	0.348	4.156	3.323	18.945	13.916	7.653	16.628	20.513	27.367	21.971	15.032	8.779	13.703	5.976
SLC20A1	0.833	1.781	0.009	0.223	4.453	3.620	35.794	27.727	16.831	20.152	19.643	14.435	19.050	19.749	10.972	13.985	9.361
CRCP	0.830	1.778	0.048	0.361	1.771	0.941	1.276	4.467	4.417	2.641	3.327	0.878	1.238	3.124	0.913	0.748	1.116
TMEM171	0.824	1.770	0.029	0.308	1.740	0.916	5.185	3.067	2.574	2.872	1.708	2.119	1.831	0.860	0.486	1.102	1.151
FXYD5	0.822	1.767	0.005	0.198	4.413	3.591	27.834	18.491	21.415	16.200	17.127	27.703	24.127	13.946	13.000	9.541	10.857
ANKRD37	0.816	1.760	0.024	0.289	4.319	3.503	14.342	12.696	16.245	17.170	31.988	19.306	21.547	24.346	7.775	16.568	8.459
GAS7	0.804	1.745	0.044	0.354	1.201	0.397	1.061	0.741	0.264	2.887	0.907	2.332	1.418	1.878	0.000	0.773	0.288
SCARA3	0.799	1.740	0.046	0.357	3.121	2.322	11.717	8.497	6.288	6.209	5.481	5.079	7.786	13.952	4.114	2.161	6.738
SLC16A4	0.794	1.734	0.015	0.255	2.588	1.794	4.228	3.739	4.009	5.504	4.648	6.184	5.740	6.758	1.059	4.072	2.996
GATA6	0.792	1.732	0.025	0.294	5.165	4.373	34.412	33.404	35.065	38.844	31.203	33.631	38.330	34.799	12.820	41.782	14.052
CNRIP1	0.786	1.725	0.015	0.255	3.938	3.151	14.449	21.957	13.668	20.808	10.487	11.267	11.061	14.777	5.957	11.244	7.234
TWIST2	0.785	1.723	0.040	0.339	4.053	3.268	18.566	12.398	15.977	15.060	20.497	13.732	18.688	11.963	6.813	18.301	4.932
AKR1C3	0.784	1.722	0.036	0.326	1.576	0.792	2.154	1.930	1.661	3.899	3.135	1.099	1.904	1.054	0.637	1.647	0.197
FILIP1L	0.782	1.719	0.027	0.299	4.014	3.232	12.351	10.202	19.538	17.645	11.405	15.391	12.480	28.554	7.494	6.990	11.222
FGFRL1	0.781	1.718	0.029	0.308	3.418	2.637	16.104	8.682	15.019	7.217	6.203	8.751	9.255	9.862	3.221	6.909	6.215
HOXA7	0.780	1.717	0.005	0.201	2.254	1.474	3.942	4.159	5.641	3.260	3.505	2.375	4.208	3.704	2.562	1.944	1.045
INPP1	0.780	1.717	0.001	0.119	2.564	1.784	4.024	6.583	5.441	5.274	5.022	5.365	4.299	3.787	2.162	3.292	2.011
HIF1A	0.776	1.712	0.021	0.278	7.624	6.848	186.966	225.529	135.590	257.141	239.250	161.081	239.897	161.659	179.943	79.196	104.427

TGFBR2	0.770	1.705	0.015	0.253	5.159	4.389	42.673	30.826	34.259	39.798	32.142	38.381	42.724	22.225	15.025	31.681	16.562
IDH1	0.767	1.701	0.003	0.178	4.403	3.636	21.313	18.697	17.050	20.175	26.320	19.834	18.209	20.860	8.276	17.268	10.351
AHNAK2	0.766	1.700	0.012	0.239	2.550	1.785	4.509	2.826	3.942	5.343	3.951	6.206	5.795	7.650	3.439	2.100	1.971
KDELC2	0.765	1.700	0.008	0.219	4.344	3.579	24.623	24.514	19.809	21.153	17.661	12.183	18.267	19.306	13.710	12.568	7.553
SLC22A4	0.763	1.698	0.000	0.100	1.125	0.362	1.663	0.847	0.995	0.958	1.292	1.196	1.490	1.129	0.078	0.516	0.297
LRFN4	0.759	1.693	0.007	0.214	2.168	1.409	2.733	5.012	3.546	4.689	2.690	2.608	3.723	3.559	0.922	2.456	1.818
TLCD2	0.759	1.692	0.013	0.248	2.410	1.652	6.322	2.921	5.914	3.755	3.067	3.840	4.268	5.518	1.962	3.345	1.410
ENG	0.758	1.691	0.025	0.292	6.771	6.013	85.759	99.871	95.071	117.557	96.116	110.863	129.249	142.506	53.103	115.316	41.796
CRK	0.756	1.689	0.001	0.124	4.757	4.001	18.009	29.591	32.209	27.754	27.076	25.998	23.958	26.182	15.027	17.649	12.724
SNX12	0.754	1.686	0.019	0.269	4.008	3.254	16.962	16.854	17.634	10.205	17.791	14.189	14.341	14.294	15.430	6.072	6.476
FKBP11	0.753	1.685	0.004	0.180	3.113	2.360	7.543	9.427	7.763	9.373	4.556	9.962	7.446	6.534	3.987	4.577	3.866
COL16A1	0.752	1.684	0.010	0.228	6.046	5.295	66.772	67.229	56.956	62.824	58.134	50.196	88.347	77.714	39.697	55.269	25.409
KCNQ5	0.748	1.680	0.009	0.223	1.266	0.518	1.780	2.182	0.975	2.090	1.921	1.363	0.741	0.727	0.261	0.489	0.563
MAZ	0.747	1.678	0.016	0.258	3.893	3.146	13.793	19.781	9.791	23.446	10.026	14.394	11.889	12.391	7.975	8.545	7.105
NAAA	0.745	1.676	0.007	0.214	2.155	1.410	4.558	2.847	5.094	4.444	2.707	3.017	3.425	2.312	1.670	2.301	1.130
RBMS3	0.745	1.676	0.005	0.205	3.011	2.266	5.861	6.612	7.166	7.045	10.678	5.645	7.478	6.907	2.460	5.374	4.049
NR2F1	0.742	1.673	0.040	0.339	3.743	3.000	14.900	8.333	11.221	12.664	17.399	13.597	9.563	13.658	3.363	10.493	9.217
PRG4	0.737	1.667	0.010	0.229	0.857	0.120	1.371	0.413	1.184	1.420	0.525	0.248	0.582	1.175	0.071	0.197	0.000
PRAF2	0.734	1.663	0.027	0.302	4.614	3.880	21.798	29.890	39.057	31.984	18.088	24.017	17.252	14.906	14.381	12.671	14.178
SLC44A1	0.725	1.652	0.013	0.243	3.349	2.624	10.420	7.789	5.584	9.566	7.695	11.075	14.843	8.981	5.222	6.013	4.369
PDE10A	0.720	1.647	0.047	0.358	1.310	0.591	2.903	2.441	0.600	0.755	1.809	1.674	1.495	1.025	0.014	0.817	0.853
SNAI2	0.714	1.640	0.008	0.219	5.761	5.047	37.832	58.073	60.169	62.186	53.858	58.851	68.659	35.940	30.875	37.536	28.440
LHFPL2	0.712	1.638	0.010	0.231	2.913	2.201	5.757	9.106	6.828	9.351	5.332	7.086	5.166	4.932	2.932	5.159	3.015
C19orf48	0.708	1.634	0.007	0.214	2.056	1.348	2.936	5.625	3.176	4.197	2.740	2.537	2.388	2.517	1.299	1.690	1.665
GRIN2D	0.708	1.633	0.022	0.282	1.252	0.544	2.574	1.055	2.045	0.858	0.804	0.905	2.081	1.347	0.199	0.905	0.357
H2AFY2	0.705	1.631	0.000	0.062	3.310	2.605	8.471	11.586	9.148	10.125	7.877	8.226	8.178	8.249	4.973	5.517	4.780
C8orf33	0.705	1.630	0.009	0.223	3.254	2.549	8.059	5.668	9.766	7.533	7.814	9.947	10.331	10.299	4.181	7.330	3.645
CYBA	0.702	1.626	0.015	0.255	4.221	3.519	21.463	20.819	23.539	16.256	10.355	16.682	17.419	18.029	9.913	14.121	8.129
CENPX	0.697	1.621	0.035	0.324	2.045	1.348	4.217	6.038	2.771	4.058	3.178	1.623	2.097	2.536	1.168	1.701	1.816
ACVRL1	0.693	1.616	0.022	0.282	2.005	1.312	3.980	2.340	3.772	2.348	2.490	2.611	3.173	3.825	1.426	2.886	0.625
FLNB	0.690	1.614	0.015	0.255	5.323	4.633	52.659	52.736	30.441	29.896	37.930	34.244	53.842	30.363	26.305	20.719	24.767
CNDP2	0.685	1.608	0.011	0.236	1.934	1.249	3.413	4.209	2.827	3.080	1.799	3.127	1.641	3.144	1.137	1.128	1.950
CDC42EP2	0.682	1.605	0.035	0.324	3.967	3.285	15.410	17.992	14.514	15.485	17.457	13.154	13.335	10.985	4.456	14.155	10.193
OLFM1	0.682	1.604	0.003	0.177	1.183	0.502	0.968	0.914	1.505	0.919	1.407	1.122	1.771	1.764	0.092	0.665	0.561
ZFPM1	0.680	1.603	0.004	0.183	1.706	1.026	2.991	1.780	1.946	2.478	1.799	2.138	2.883	2.319	1.073	1.664	0.529
PROSER2	0.680	1.602	0.005	0.201	1.074	0.394	1.534	0.733	2.032	0.882	1.241	0.846	0.699	1.189	0.138	0.405	0.419
XRCC4	0.672	1.594	0.036	0.327	2.236	1.563	3.455	4.679	8.208	3.929	2.324	2.893	3.121	2.953	1.757	2.183	1.940
WARS	0.672	1.593	0.026	0.298	5.210	4.538	32.313	38.880	31.117	42.475	36.677	43.550	33.049	32.162	20.980	38.008	13.614
HOXC5	0.669	1.590	0.002	0.154	1.289	0.620	1.828	1.097	1.048	2.071	1.567	1.076	1.460	1.605	0.591	0.778	0.283
CBLL1	0.668	1.589	0.001	0.123	2.142	1.474	2.777	3.670	3.963	3.689	4.099	2.863	3.313	3.126	2.086	1.158	2.217
MFSD7	0.668	1.588	0.013	0.245	0.939	0.271	1.356	0.443	0.671	0.677	0.752	0.672	1.622	1.490	0.000	0.517	0.158
MAPK14	0.666	1.587	0.025	0.292	3.062	2.395	7.092	9.606	7.843	8.756	4.369	7.187	8.156	6.924	4.068	2.678	6.813
RNASEK-C17c	0.666	1.587	0.042	0.345	2.996	2.330	4.662	4.842	9.376	9.356	6.732	7.654	7.114	7.525	2.193	6.588	4.252
BOC	0.665	1.585	0.029	0.308	1.334	0.669	1.362	0.778	1.479	1.266	1.163	1.567	2.862	2.229	0.114	1.133	0.693
HGD	0.664	1.584	0.031	0.314	0.860	0.196	0.772	1.251	1.890	1.189	1.029	0.447	0.186	0.342	0.132	0.177	0.129
MFSD1	0.663	1.583	0.001	0.130	3.919	3.256	16.442	11.511	14.306	15.163	11.222	15.884	15.646	13.789	7.488	10.766	7.730
MBNL2	0.661	1.581	0.032	0.318	5.154	4.492	39.890	30.030	24.278	35.670	34.552	39.154	36.974	39.421	12.674	34.388	22.565
DDAH2	0.659	1.579	0.012	0.240	3.935	3.276	10.841	18.560	20.269	17.318	12.301	11.111	11.830	15.022	10.291	7.365	8.615
LY6E	0.658	1.578	0.022	0.284	4.517	3.859	24.940	19.756	30.105	13.002	16.984	22.239	24.568	29.084	15.396	11.714	13.647
MGST1	0.657	1.576	0.001	0.123	3.689	3.033	13.876	10.919	12.471	11.319	12.140	10.807	12.270	11.672	8.931	8.116	5.056
ZNF641	0.655	1.574	0.048	0.361	1.278	0.623	1.859	0.660	1.451	2.323	0.455	1.656	1.229	2.590	0.360	0.398	0.921
TIAM1	0.655	1.574	0.026	0.294	1.441	0.787	2.077	2.861	0.748	2.739	1.427	2.160	1.368	1.101	0.600	0.840	0.745
PTX3	0.654	1.573	0.038	0.334	9.744	9.091	842.321	824.780	814.488	714.202	912.640	1008.985	950.495	820.675	338.542	994.041	478.923

RNF138	0.647	1.565	0.044	0.354	2.045	1.398	2.434	3.807	3.280	4.102	4.121	4.226	1.492	2.494	1.057	1.283	2.898
NFIA	0.646	1.564	0.013	0.248	1.846	1.200	2.407	3.247	4.184	2.449	2.473	1.884	1.891	2.718	0.822	2.121	1.133
ZDHHC18	0.641	1.559	0.001	0.139	1.954	1.313	3.064	3.204	2.820	3.090	2.793	2.896	2.419	2.756	1.020	2.426	1.215
SLC16A5	0.639	1.557	0.043	0.350	1.673	1.034	2.580	2.222	1.654	1.292	1.656	1.492	3.667	3.942	0.778	1.684	0.801
HOXC8	0.638	1.556	0.043	0.348	4.041	3.403	12.913	11.611	17.396	16.700	25.199	17.858	13.466	12.182	13.384	10.715	6.024
SLC1A5	0.637	1.556	0.008	0.219	6.723	6.086	85.990	88.027	98.101	84.132	115.182	133.443	130.740	114.377	61.286	85.697	56.998
POPDC3	0.637	1.555	0.004	0.189	2.538	1.901	5.204	5.336	5.128	5.973	5.472	4.860	3.664	3.351	3.120	3.207	2.004
PITX2	0.635	1.553	0.049	0.362	0.960	0.325	1.156	1.189	2.171	0.460	1.223	0.590	1.054	0.290	0.087	0.729	0.046
AKR1C2	0.635	1.553	0.010	0.224	1.302	0.667	2.015	0.938	1.942	1.805	0.934	0.986	2.198	1.305	0.646	0.757	0.384
FLRT3	0.634	1.552	0.000	0.089	0.891	0.257	1.124	0.542	0.688	0.827	0.994	0.747	1.163	0.844	0.093	0.257	0.242
TNFSF12	0.634	1.552	0.017	0.263	2.281	1.647	2.777	3.407	2.493	5.205	5.409	4.593	3.362	4.519	1.752	1.838	2.934
ACLY	0.634	1.551	0.008	0.220	5.974	5.341	62.643	68.819	48.766	81.020	54.379	59.746	65.566	59.119	28.513	41.497	52.059
RAB13	0.633	1.551	0.011	0.236	5.469	4.835	44.230	26.064	53.133	42.595	56.159	43.466	45.620	42.160	26.529	31.397	25.094
UCHL1	0.631	1.549	0.023	0.284	6.920	6.289	147.835	136.694	92.255	117.488	119.429	117.334	117.470	119.786	123.413	71.099	52.257
TSKU	0.630	1.548	0.008	0.219	3.208	2.578	8.942	9.250	7.392	8.706	5.714	7.909	10.551	8.285	3.484	6.590	5.257
IRAK3	0.630	1.547	0.026	0.299	1.681	1.051	2.488	1.644	1.526	2.294	2.995	2.570	2.641	1.799	0.835	2.267	0.483
AMMECR1	0.627	1.545	0.006	0.214	2.944	2.317	6.929	7.577	6.087	7.738	7.155	6.090	6.820	5.467	3.469	6.226	2.830
MID1IP1	0.625	1.543	0.022	0.282	3.188	2.562	7.377	10.476	7.149	12.653	4.876	8.698	9.135	6.687	4.978	5.234	4.529
TSHZ1	0.624	1.542	0.017	0.264	2.100	1.475	3.792	2.919	1.912	4.827	4.159	2.686	3.696	3.004	1.353	2.585	1.548
TXN	0.622	1.539	0.049	0.362	7.135	6.514	159.197	164.517	187.486	196.760	162.479	103.317	99.874	88.843	79.315	102.664	90.655
TGFB1	0.620	1.537	0.006	0.214	5.125	4.505	32.542	38.831	45.649	33.412	25.508	32.046	36.915	29.928	16.907	25.570	23.629
ZNF503	0.619	1.536	0.044	0.354	4.499	3.880	11.542	24.617	22.867	24.571	24.822	21.574	30.862	17.745	10.248	16.564	15.140
EMP1	0.618	1.535	0.020	0.276	4.929	4.311	41.084	28.280	26.209	21.161	26.287	28.742	35.846	32.462	22.126	23.034	13.079
SLC50A1	0.617	1.533	0.003	0.178	3.062	2.446	8.477	6.999	9.433	8.086	8.233	5.103	6.104	7.233	3.893	4.995	4.511
PLAUR	0.615	1.531	0.034	0.324	3.267	2.652	12.653	8.467	9.159	6.640	6.413	10.116	9.696	7.343	3.474	4.956	8.323
NCOA4	0.614	1.531	0.005	0.201	5.066	4.451	31.778	24.317	42.408	36.014	35.507	37.882	29.272	26.642	21.536	23.305	18.118
IL21R	0.614	1.530	0.020	0.273	0.896	0.282	0.481	0.582	1.626	0.593	0.847	0.405	1.477	1.281	0.111	0.353	0.196
PPIB	0.614	1.530	0.006	0.213	8.128	7.514	337.861	246.410	304.115	291.431	231.815	333.764	244.375	261.016	141.244	236.681	179.683
LIMK1	0.613	1.529	0.015	0.255	2.612	1.999	7.999	3.889	6.491	5.469	4.045	3.908	5.336	4.820	3.861	2.492	2.760
HOXA2	0.613	1.529	0.032	0.317	1.638	1.025	2.324	1.585	1.794	2.046	1.363	2.649	4.101	1.740	0.939	1.707	0.607
TMTC2	0.612	1.528	0.010	0.223	1.114	0.502	1.399	0.588	1.432	1.060	1.322	0.810	1.245	1.676	0.073	0.729	0.532
FKBP9	0.611	1.527	0.031	0.315	5.488	4.877	43.904	35.206	46.595	36.867	42.127	36.584	54.758	61.156	20.547	43.470	25.477
AUTS2	0.610	1.526	0.009	0.223	1.515	0.905	1.328	2.144	1.088	1.817	1.804	2.214	2.412	2.353	0.659	1.427	0.630
HYOU1	0.606	1.522	0.049	0.362	4.571	3.965	22.413	28.293	19.601	27.182	15.020	26.528	22.044	24.203	9.586	24.382	13.166
DSCC1	0.602	1.518	0.023	0.284	1.722	1.120	2.408	4.312	2.689	2.956	1.674	1.600	1.904	1.633	1.100	1.369	1.066
ANTXR2	0.599	1.515	0.009	0.223	5.054	4.456	39.002	30.788	22.488	28.412	27.301	40.097	35.248	39.156	19.591	23.737	19.737
LMAN2L	0.598	1.514	0.002	0.156	3.006	2.408	7.425	8.352	9.401	6.315	5.729	6.379	6.707	6.549	4.021	5.088	3.886
SLC31A2	0.596	1.511	0.024	0.289	2.355	1.760	5.004	2.275	4.094	3.722	4.379	4.337	3.717	6.338	1.819	3.225	2.260
BACE2	0.594	1.509	0.038	0.333	1.927	1.333	1.882	2.127	1.873	2.321	3.643	4.256	3.648	3.477	0.867	2.023	1.834
ECHDC1	0.589	1.505	0.009	0.223	2.902	2.313	6.915	6.738	7.329	7.747	6.812	5.961	5.487	5.190	3.647	5.952	2.794
ABHD14A	0.587	1.502	0.008	0.218	1.800	1.213	1.967	2.290	3.705	3.251	1.717	2.491	2.113	2.746	1.477	1.550	0.971
NUP50	0.587	1.502	0.024	0.287	2.718	2.131	5.206	8.112	4.796	6.191	5.553	5.104	5.006	5.198	1.828	4.574	4.330
BLOC1S2	0.586	1.501	0.006	0.214	3.622	3.036	9.562	10.712	14.257	12.493	9.259	14.502	11.185	9.675	6.802	9.118	5.987
ACAN	-4.112	0.058	0.004	0.180	1.581	5.693	1.858	0.854	6.514	0.423	5.602	0.093	1.332	5.752	306.415	7.483	52.135
ELN	-3.780	0.073	0.002	0.154	3.347	7.126	1.520	12.839	22.977	23.673	8.883	1.743	18.915	9.309	330.778	106.360	75.575
ITGA7	-3.400	0.095	0.001	0.131	2.507	5.908	1.809	3.678	20.025	8.012	12.788	1.933	1.266	3.787	69.175	31.525	93.822
IGFBP2	-3.336	0.099	0.009	0.222	2.271	5.607	7.149	17.669	32.365	2.810	1.187	0.787	0.658	1.350	68.887	39.305	40.136
ACTG2	-3.235	0.106	0.015	0.255	2.939	6.174	2.378	18.105	44.829	12.051	12.291	4.363	0.430	2.045	107.622	23.140	142.524
PLPPR4	-3.213	0.108	0.002	0.157	0.836	4.049	0.124	2.029	1.321	1.810	2.382	0.044	0.214	0.083	66.913	4.097	12.115
BEX1	-2.951	0.129	0.007	0.214	2.747	5.698	5.055	13.320	25.818	3.978	13.825	3.125	3.084	0.427	86.865	27.378	55.094
C11orf96	-2.873	0.136	0.000	0.062	1.137	4.010	0.487	0.933	1.201	3.735	1.771	0.690	0.710	1.277	24.374	6.770	20.212

CYTIP	-2.867	0.137	0.000	0.028	0.397	3.264	0.606	0.435	2.120	0.142	0.000	0.000	0.099	0.000	9.161	8.528	8.161
CSPG4	-2.756	0.148	0.015	0.254	1.832	4.588	1.539	17.067	11.636	1.706	1.154	0.638	1.491	0.868	47.150	6.513	37.419
GALNT5	-2.677	0.156	0.001	0.123	2.749	5.426	4.273	17.735	11.245	7.226	6.513	2.930	3.767	1.976	63.883	26.620	43.303
CDK18	-2.673	0.157	0.002	0.157	0.405	3.077	0.000	0.029	4.666	0.172	0.082	0.074	0.149	0.033	15.470	2.556	9.264
MCAM	-2.499	0.177	0.022	0.283	1.284	3.784	0.306	8.819	9.053	1.535	0.469	0.447	0.228	0.451	27.621	3.244	20.499
HEYL	-2.491	0.178	0.002	0.157	0.412	2.903	0.000	0.129	1.806	0.739	0.474	0.115	0.037	0.048	22.675	2.117	4.670
SUSD2	-2.424	0.186	0.006	0.205	0.588	3.012	0.145	0.733	0.716	1.150	0.754	0.000	0.568	0.296	15.722	0.651	18.005
SCN3A	-2.403	0.189	0.000	0.046	0.423	2.826	0.712	0.151	0.205	1.029	0.803	0.025	0.032	0.136	9.395	7.851	2.874
MYH11	-2.336	0.198	0.000	0.100	0.312	2.648	0.006	0.044	2.783	0.143	0.197	0.005	0.010	0.020	7.019	3.432	5.925
DES	-2.292	0.204	0.011	0.236	0.633	2.925	0.110	0.347	1.730	0.220	0.377	0.155	0.572	1.688	12.600	0.503	20.428
NTRK2	-2.285	0.205	0.000	0.105	0.349	2.633	0.018	0.024	2.853	0.345	0.181	0.008	0.002	0.073	6.462	3.149	6.715
HSPB3	-2.276	0.206	0.004	0.180	0.073	2.349	0.000	0.000	0.207	0.113	0.113	0.000	0.000	0.000	20.312	2.090	1.009
ALPL	-2.245	0.211	0.035	0.324	1.565	3.811	0.936	3.422	0.354	9.533	1.918	0.402	1.416	3.874	1.866	17.918	49.958
LMOD1	-2.219	0.215	0.021	0.279	3.678	5.897	6.841	8.881	27.698	18.083	36.040	5.456	2.205	21.186	103.200	25.287	76.237
HSPB7	-2.214	0.216	0.003	0.178	2.728	4.942	4.692	8.132	11.352	3.983	4.528	1.580	7.392	8.686	45.539	11.438	49.177
JPH2	-2.214	0.216	0.005	0.201	1.315	3.528	0.268	1.445	5.238	1.441	2.759	0.555	1.181	1.438	24.520	3.215	13.284
LBH	-2.212	0.216	0.001	0.131	3.060	5.272	7.184	5.202	12.093	6.734	9.771	8.390	3.341	9.360	58.922	14.123	62.641
RGS5	-2.210	0.216	0.001	0.105	0.861	3.071	0.818	0.468	0.985	0.774	0.642	0.212	0.630	2.885	8.208	3.195	14.361
TNFSF15	-2.208	0.216	0.013	0.248	0.722	2.930	0.000	0.169	1.857	2.326	3.825	0.021	0.000	0.000	16.211	1.652	8.689
TM4SF1	-2.199	0.218	0.004	0.196	2.165	4.364	2.792	12.088	5.070	5.239	3.180	0.792	2.691	2.152	26.745	8.794	31.151
PALMD	-2.181	0.221	0.000	0.062	0.299	2.480	0.193	0.126	0.078	1.041	0.489	0.010	0.107	0.065	5.528	1.960	7.984
CLEC3B	-2.179	0.221	0.029	0.308	3.792	5.971	7.080	14.217	78.966	21.429	22.276	4.245	2.703	12.573	57.514	51.109	79.885
EREG	-2.134	0.228	0.000	0.028	0.266	2.400	0.000	0.174	0.321	0.629	0.586	0.021	0.071	0.000	6.287	5.027	2.347
MAP3K7CL	-2.128	0.229	0.019	0.269	2.509	4.637	3.257	4.621	32.855	6.314	3.077	3.464	0.679	5.084	20.392	18.994	35.032
ANO1	-2.119	0.230	0.008	0.219	0.140	2.260	0.000	0.047	1.065	0.000	0.000	0.000	0.007	0.000	19.867	1.131	1.471
GDF5	-2.118	0.230	0.020	0.275	1.415	3.533	0.414	2.971	4.378	6.091	3.528	0.129	0.600	0.462	32.292	6.380	5.318
LEP	-2.070	0.238	0.005	0.205	0.146	2.216	0.000	0.000	0.069	0.026	0.139	0.093	0.243	0.325	0.222	12.508	5.073
SEMA3F	-2.017	0.247	0.001	0.106	0.471	2.488	0.180	0.110	1.347	0.609	0.513	0.097	0.415	0.172	10.553	1.806	4.444
KLF5	-1.996	0.251	0.002	0.157	1.066	3.061	1.872	0.414	1.548	0.457	0.342	0.640	1.323	3.780	15.512	5.247	4.638
PLCB4	-1.986	0.252	0.000	0.105	1.223	3.209	1.535	0.881	4.497	1.461	1.554	0.796	0.469	1.029	11.610	6.004	7.959
PPL	-1.983	0.253	0.001	0.105	0.883	2.866	0.044	1.809	0.424	2.116	1.793	0.718	0.398	0.532	9.832	5.608	4.410
KRTAP1-5	-1.931	0.262	0.004	0.194	1.052	2.984	2.844	0.481	3.852	0.815	0.607	0.285	1.858	0.157	4.504	5.511	12.815
TGM2	-1.923	0.264	0.009	0.223	4.171	6.095	14.989	12.505	16.152	47.018	11.882	9.692	32.072	12.694	27.302	85.004	130.106
MRVI1	-1.916	0.265	0.016	0.260	3.398	5.314	8.919	13.349	16.819	8.351	28.577	1.974	5.622	10.034	68.472	20.235	41.685
FGF5	-1.902	0.268	0.007	0.214	2.228	4.130	7.182	4.870	8.063	2.545	3.801	2.100	2.296	2.072	42.176	6.874	14.794
RDH5	-1.882	0.271	0.001	0.109	0.958	2.840	1.051	0.613	2.368	1.306	1.138	0.417	0.453	0.795	8.828	2.520	9.616
KIAA1644	-1.853	0.277	0.000	0.043	0.795	2.648	0.436	0.578	1.140	2.119	0.858	0.507	0.618	0.202	5.578	5.308	4.938
ATP8B1	-1.835	0.280	0.008	0.217	3.493	5.328	18.895	12.575	21.438	4.943	18.769	6.293	3.576	9.877	48.142	28.487	43.723
HBA2	-1.827	0.282	0.001	0.119	0.235	2.062	0.000	0.000	0.000	0.000	0.000	0.099	2.349	0.000	2.902	2.888	3.795
MSRB3	-1.820	0.283	0.019	0.272	2.748	4.568	6.069	4.352	5.471	6.062	8.924	3.171	10.218	4.173	55.651	4.682	40.491
RAMP1	-1.802	0.287	0.015	0.253	1.037	2.839	0.869	0.246	0.992	0.000	1.088	1.275	3.354	2.285	17.410	1.778	6.169
IL7R	-1.793	0.288	0.005	0.201	1.703	3.496	5.240	3.877	2.622	1.004	3.813	0.594	2.726	0.995	14.503	5.294	13.719
ADAMTS5	-1.775	0.292	0.005	0.201	1.433	3.208	0.302	0.526	6.532	1.452	1.352	2.066	2.542	2.011	5.889	8.378	11.220
RAB11FIP1	-1.769	0.293	0.000	0.105	1.224	2.993	0.740	0.478	2.598	0.528	1.624	1.285	1.686	2.894	8.103	6.329	6.569
ACTA2	-1.769	0.293	0.023	0.285	7.979	9.749	108.809	299.190	462.470	330.109	550.170	188.699	89.901	341.265	1527.828	401.138	1034.288
ARSJ	-1.762	0.295	0.001	0.131	2.631	4.393	4.568	6.584	9.165	6.426	5.698	3.856	3.085	4.127	27.932	9.283	30.178
IL34	-1.748	0.298	0.000	0.077	0.256	2.003	0.000	0.043	0.249	0.344	0.874	0.197	0.050	0.000	2.757	1.626	5.532
SYNPO2	-1.742	0.299	0.041	0.343	4.480	6.222	13.371	28.571	38.363	32.287	56.997	9.468	5.251	28.137	115.716	30.578	111.918
SYTL2	-1.733	0.301	0.001	0.105	1.016	2.750	0.275	0.531	1.342	1.731	2.528	0.747	0.845	0.977	7.716	3.270	7.173
ITGA3	-1.726	0.302	0.007	0.214	3.181	4.907	7.352	11.118	17.002	4.067	9.589	4.798	5.549	11.347	52.153	12.401	36.898
SCUBE3	-1.719	0.304	0.031	0.315	4.283	6.002	28.871	37.022	37.202	12.249	16.592	4.033	9.345	38.248	94.281	41.754	63.610
TNS3	-1.713	0.305	0.005	0.199	1.682	3.396	0.738	2.039	5.539	1.030	3.294	1.151	3.453	2.903	17.657	7.727	6.160
HBA1	-1.707	0.306	0.001	0.107	0.235	1.942	0.000	0.000	0.000	0.000	0.106	0.102	2.013	0.000	2.551	2.471	3.601

LAMA5	-1.686	0.311	0.042	0.348	2.277	3.963	1.325	8.634	7.658	7.798	8.494	2.528	1.258	1.353	35.190	5.599	14.879
EDN1	-1.677	0.313	0.037	0.332	1.345	3.021	1.306	1.494	12.762	0.774	2.078	0.499	0.510	0.770	6.724	3.543	14.256
GALNT18	-1.660	0.317	0.001	0.127	0.954	2.613	0.813	0.330	2.934	0.679	0.910	0.797	0.810	1.001	4.429	2.987	9.579
GSTA1	-1.649	0.319	0.001	0.131	0.097	1.746	0.000	0.000	0.000	0.000	0.087	0.000	0.066	0.478	5.119	0.476	3.180
RBM24	-1.648	0.319	0.002	0.159	1.291	2.939	0.779	2.263	4.674	0.736	1.234	1.058	2.119	0.569	7.610	4.483	8.557
CPED1	-1.638	0.321	0.000	0.105	1.491	3.129	1.090	2.404	2.792	2.249	1.319	2.691	2.368	0.541	8.862	5.623	9.248
PTGES3L-AAR	-1.632	0.323	0.022	0.284	0.405	2.037	0.038	0.113	5.272	0.037	0.109	0.036	0.000	0.096	6.487	1.969	2.112
SGCA	-1.626	0.324	0.010	0.228	1.232	2.858	0.600	2.630	3.677	0.367	0.814	0.593	2.620	1.392	11.610	2.505	7.623
MYOCD	-1.620	0.325	0.002	0.159	1.821	3.441	4.768	0.645	2.747	1.512	5.102	2.189	2.685	2.793	13.749	7.273	9.487
CRYAB	-1.617	0.326	0.014	0.251	3.770	5.387	11.937	16.846	28.245	10.389	13.497	7.137	7.649	14.279	84.860	15.106	52.020
PRUNE2	-1.603	0.329	0.015	0.255	2.715	4.318	5.633	3.507	4.496	8.260	14.840	3.281	1.636	11.692	13.710	18.425	26.787
CCBE1	-1.600	0.330	0.008	0.219	1.862	3.462	2.314	3.465	5.346	4.743	1.912	2.264	2.141	0.897	18.594	3.945	12.817
EFHD1	-1.598	0.330	0.021	0.278	0.922	2.520	0.073	0.660	3.744	1.134	0.775	0.201	0.310	2.300	12.249	1.819	4.052
CCDC190	-1.585	0.333	0.000	0.105	0.308	1.893	0.212	1.018	0.185	0.249	0.136	0.020	0.021	0.287	4.573	1.162	3.250
CADM4	-1.580	0.334	0.000	0.086	0.804	2.384	0.156	0.355	1.469	0.591	1.142	0.848	1.198	0.614	5.848	2.859	4.387
ADGRE5	-1.575	0.336	0.003	0.176	2.931	4.507	5.177	12.850	10.891	6.070	6.283	4.584	6.592	4.166	29.607	10.969	31.067
BCAM	-1.572	0.336	0.003	0.179	2.067	3.639	1.856	3.880	3.115	2.036	5.493	2.471	4.040	3.804	25.134	5.645	10.145
TMEM158	-1.559	0.339	0.002	0.156	1.849	3.408	1.672	1.565	1.639	2.719	3.588	3.249	5.146	2.523	4.947	14.037	12.372
SLC9A3R1	-1.555	0.340	0.001	0.123	0.777	2.332	0.455	0.684	1.563	1.643	0.550	0.519	0.367	0.392	5.067	1.861	6.350
CASQ2	-1.554	0.341	0.000	0.046	0.097	1.652	0.000	0.000	0.117	0.159	0.287	0.031	0.000	0.000	3.764	0.995	2.263
FP15737	-1.554	0.341	0.014	0.253	0.774	2.329	0.242	0.429	0.382	0.843	0.255	0.234	5.978	0.502	3.777	5.523	3.068
KLHL30	-1.538	0.344	0.014	0.248	0.628	2.166	0.000	1.180	2.005	0.450	1.721	0.152	0.029	0.062	8.010	1.338	3.293
INAFM2	-1.528	0.347	0.008	0.219	3.372	4.900	7.560	10.116	18.347	7.560	13.016	5.569	7.349	9.921	68.135	15.353	22.547
FAM107B	-1.526	0.347	0.005	0.198	1.376	2.901	2.222	2.621	4.613	1.386	1.841	0.888	0.521	0.611	8.149	4.201	7.765
ALDH1A1	-1.524	0.348	0.010	0.228	0.175	1.699	0.807	0.101	0.085	0.031	0.000	0.045	0.000	0.136	6.605	0.161	2.875
SYNM	-1.522	0.348	0.006	0.213	1.627	3.148	1.404	3.058	3.077	2.493	1.806	1.935	1.488	1.908	18.715	2.670	8.632
PRKAG2	-1.505	0.352	0.003	0.160	2.384	3.888	3.206	3.957	6.873	3.705	6.768	3.679	2.493	4.610	24.343	6.919	15.180
PGM5	-1.500	0.354	0.004	0.185	1.035	2.534	1.186	0.369	0.800	0.572	1.125	1.182	0.730	3.570	4.982	2.469	8.367
SORT1	-1.482	0.358	0.014	0.253	2.377	3.860	3.388	4.458	10.359	2.892	4.517	1.860	2.354	8.472	22.978	6.904	15.143
RRAD	-1.464	0.362	0.018	0.268	1.317	2.781	0.120	0.286	4.697	1.370	2.060	2.998	1.673	1.333	10.296	5.170	3.662
ENDOD1	-1.459	0.364	0.025	0.294	3.288	4.747	8.021	15.894	15.453	10.625	14.995	4.892	2.777	6.998	51.200	14.497	22.941
PRSS12	-1.443	0.368	0.003	0.160	3.570	5.013	16.834	8.884	16.841	5.419	16.503	8.943	8.325	11.081	32.178	22.735	41.722
CAMK2N1	-1.440	0.369	0.008	0.219	1.750	3.190	1.750	0.768	3.753	1.498	3.108	2.055	5.114	2.705	9.402	3.908	13.885
GPRC5A	-1.430	0.371	0.018	0.268	3.075	4.505	16.416	4.864	9.263	4.324	10.190	6.320	5.693	7.291	8.036	35.181	34.789
KIAA1217	-1.395	0.380	0.000	0.094	0.457	1.852	0.301	0.267	0.612	1.316	0.217	0.164	0.169	0.238	3.406	1.646	3.032
SUSD5	-1.384	0.383	0.012	0.239	1.276	2.661	0.913	4.565	3.402	1.581	0.781	0.430	1.156	0.784	5.306	3.428	8.055
C10orf10	-1.384	0.383	0.008	0.219	3.817	5.200	4.969	10.637	16.346	11.725	18.963	10.101	17.123	24.271	38.912	45.556	25.754
FOXC1	-1.378	0.385	0.001	0.127	1.323	2.701	0.565	1.185	1.365	1.292	2.683	2.863	1.429	1.390	5.482	3.632	8.155
MTSS1L	-1.371	0.387	0.011	0.234	2.592	3.963	2.993	6.155	6.504	6.628	8.187	2.712	5.000	4.211	29.988	6.557	15.195
ITIH5	-1.371	0.387	0.032	0.317	0.994	2.365	0.000	0.112	3.225	0.751	0.496	2.118	0.905	2.387	1.637	8.336	4.553
NPTX1	-1.368	0.388	0.009	0.223	0.635	2.003	1.099	1.128	0.661	0.628	0.779	0.044	0.346	0.123	8.150	1.239	2.144
CSRP2	-1.367	0.388	0.002	0.157	2.201	3.567	4.223	5.028	6.580	3.529	3.053	1.480	3.818	2.815	13.105	7.229	13.353
KCNMB1	-1.346	0.393	0.015	0.255	0.360	1.706	0.000	0.573	2.275	0.375	0.024	0.000	0.000	0.015	4.826	0.786	2.337
SNTB1	-1.340	0.395	0.009	0.223	2.634	3.974	1.701	9.198	3.537	5.794	8.355	5.668	6.341	4.671	19.868	9.112	17.406
AFF3	-1.337	0.396	0.005	0.201	1.474	2.811	1.662	3.959	0.563	1.517	1.616	1.134	2.181	2.842	10.581	4.571	4.358
NLRP10	-1.336	0.396	0.001	0.105	0.639	1.975	0.584	0.112	1.533	0.735	0.311	0.521	0.569	0.424	4.420	3.056	1.761
RAPGEF5	-1.331	0.397	0.019	0.272	0.215	1.546	0.000	0.009	1.594	0.026	0.155	0.018	0.022	0.018	0.272	2.035	5.445
ITIH3	-1.330	0.398	0.001	0.119	0.332	1.662	0.189	0.175	0.462	0.242	0.360	0.049	0.084	0.607	3.705	0.764	2.816
SLC20A2	-1.321	0.400	0.017	0.262	2.662	3.983	5.941	6.144	8.977	3.936	10.655	2.906	2.759	5.167	30.789	9.196	11.194
CACNA1H	-1.317	0.401	0.027	0.302	0.877	2.194	0.042	1.417	0.162	0.877	0.982	2.172	0.579	1.374	3.484	1.031	9.523
BCL7A	-1.315	0.402	0.001	0.127	1.283	2.598	2.580	1.622	1.839	0.848	0.789	1.368	1.579	1.286	2.866	5.164	8.311
ERRFI1	-1.303	0.405	0.006	0.205	4.421	5.724	23.200	19.022	48.104	16.663	15.383	13.575	17.657	22.735	51.524	36.882	73.230
MYLK	-1.296	0.407	0.040	0.341	3.418	4.714	4.854	4.894	16.378	6.668	17.140	10.555	8.167	18.312	51.780	11.546	26.303

SPEG	-1.295	0.408	0.002	0.144	2.118	3.413	1.799	2.836	3.642	3.798	5.087	2.871	3.242	4.267	14.660	5.540	10.795
SEMA5B	-1.291	0.409	0.007	0.214	0.056	1.348	0.000	0.000	0.367	0.000	0.000	0.000	0.000	0.000	5.193	0.720	0.548
RGS16	-1.288	0.410	0.007	0.214	0.852	2.140	0.460	0.930	0.550	1.283	2.857	0.518	0.126	0.711	5.588	1.885	3.504
FHOD3	-1.281	0.411	0.007	0.214	1.584	2.865	1.191	1.491	3.874	1.796	3.731	2.225	1.301	1.495	12.709	3.885	4.774
SLC6A6	-1.279	0.412	0.021	0.281	2.778	4.057	7.740	3.216	11.937	11.604	4.878	3.283	4.561	4.827	26.273	17.510	8.135
MPP7	-1.274	0.413	0.000	0.035	0.515	1.790	0.502	0.161	0.675	0.671	0.813	0.329	0.143	0.298	2.032	2.533	2.859
TRPC6	-1.269	0.415	0.001	0.123	0.105	1.374	0.033	0.017	0.287	0.107	0.112	0.027	0.049	0.000	1.262	0.631	3.721
RASGRP1	-1.263	0.417	0.022	0.282	0.949	2.212	0.539	1.077	1.994	0.995	2.222	0.790	0.275	0.376	1.575	2.539	9.923
NOTCH3	-1.254	0.419	0.030	0.310	4.437	5.691	20.850	34.716	42.834	16.432	26.718	8.635	14.816	18.281	84.360	27.542	55.648
ALDH1B1	-1.251	0.420	0.021	0.278	4.286	5.537	15.401	10.197	14.746	13.782	25.121	20.564	20.698	39.207	30.225	32.387	94.980
ATF3	-1.246	0.422	0.013	0.246	1.347	2.593	0.720	2.092	1.477	2.936	1.684	0.754	1.619	1.742	10.911	1.876	5.416
SLC40A1	-1.245	0.422	0.026	0.298	0.855	2.100	0.214	0.483	0.296	1.950	3.289	0.424	0.589	0.717	6.579	3.458	1.332
ATP2B4	-1.241	0.423	0.001	0.131	4.080	5.321	23.763	16.979	11.681	13.659	20.222	10.010	18.643	16.598	45.548	27.429	47.278
ARHGAP29	-1.241	0.423	0.002	0.154	2.078	3.318	5.756	1.310	2.902	3.191	4.327	3.004	2.921	3.729	7.743	8.096	11.483
ARHGEF10L	-1.240	0.423	0.007	0.214	1.316	2.556	1.040	1.672	3.073	1.885	1.377	1.091	1.198	1.109	10.130	2.118	4.855
AC009779.3	-1.237	0.424	0.005	0.201	1.040	2.277	0.744	0.723	2.344	1.853	1.527	0.837	0.297	0.848	4.525	1.968	5.937
LDB3	-1.236	0.425	0.011	0.236	0.314	1.550	0.047	0.227	1.587	0.040	0.350	0.039	0.089	0.083	4.484	0.613	1.839
MYPN	-1.234	0.425	0.035	0.324	0.428	1.662	0.896	0.470	0.215	0.447	0.896	0.012	0.125	0.013	8.694	0.891	0.728
MTSS1	-1.222	0.429	0.027	0.302	1.116	2.338	0.391	0.689	1.346	0.850	4.119	2.309	0.778	0.584	8.475	3.605	1.961
CITED2	-1.217	0.430	0.015	0.255	4.834	6.050	26.825	19.167	47.360	14.229	28.083	22.424	43.984	33.546	88.092	36.805	85.442
FAM212B	-1.212	0.432	0.019	0.269	2.425	3.637	2.637	4.788	11.426	4.578	7.293	3.332	1.549	4.180	12.701	12.099	9.725
MAP3K5	-1.184	0.440	0.014	0.248	1.067	2.251	0.796	1.710	3.418	0.624	0.939	0.465	0.513	1.477	7.084	2.398	2.928
GFRA1	-1.183	0.440	0.016	0.258	1.153	2.336	0.613	0.607	0.483	0.676	2.361	3.453	1.752	1.251	3.973	6.739	2.343
AJUBA	-1.180	0.441	0.007	0.214	2.831	4.011	6.118	6.602	9.997	6.278	9.723	3.215	5.450	4.196	23.697	9.667	14.911
RNF144B	-1.180	0.441	0.000	0.022	0.496	1.676	0.231	0.273	0.382	0.265	0.356	0.493	0.698	0.665	2.058	1.840	2.761
FAM162B	-1.170	0.445	0.035	0.324	1.305	2.475	0.858	2.178	1.028	4.024	3.820	0.794	0.562	0.710	8.967	3.374	2.939
MATN2	-1.159	0.448	0.035	0.324	1.311	2.470	0.811	2.051	0.992	2.418	4.849	1.522	0.704	0.518	8.806	2.159	4.493
LIF	-1.157	0.448	0.047	0.358	2.540	3.698	7.256	8.187	11.132	6.346	3.816	1.750	4.021	1.917	11.925	7.522	18.826
NID2	-1.154	0.449	0.048	0.361	5.223	6.377	19.504	54.087	30.450	38.309	86.737	23.243	46.428	25.825	159.198	45.380	76.283
FBXL22	-1.154	0.449	0.001	0.123	0.299	1.453	0.211	0.173	0.152	0.297	0.291	0.053	0.126	0.618	3.226	0.713	1.836
TBC1D1	-1.148	0.451	0.003	0.160	2.208	3.356	3.388	4.304	6.458	2.745	3.911	2.760	3.134	3.190	14.390	5.526	9.697
THSD7B	-1.142	0.453	0.041	0.343	0.414	1.555	0.068	0.319	0.308	0.314	2.947	0.032	0.007	0.000	4.443	1.785	0.674
COPRS	-1.138	0.454	0.003	0.173	3.868	5.006	16.907	13.175	18.907	15.587	11.631	10.244	9.505	15.527	49.510	19.924	30.409
ZBTB38	-1.135	0.455	0.040	0.341	2.162	3.297	4.621	4.664	0.238	3.929	4.003	4.757	5.212	3.619	11.093	6.071	10.096
HMGA2	-1.133	0.456	0.015	0.255	1.109	2.242	1.770	2.421	1.558	1.473	1.063	0.426	0.769	0.499	6.382	1.434	4.888
STEAP1B	-1.129	0.457	0.002	0.159	0.549	1.678	0.326	0.418	0.735	0.842	0.912	0.206	0.401	0.080	4.117	1.959	1.163
IGF2BP3	-1.126	0.458	0.015	0.253	0.535	1.661	0.138	0.657	1.078	0.682	1.089	0.127	0.178	0.065	5.290	0.948	1.582
GCNT4	-1.118	0.461	0.008	0.219	1.050	2.168	0.212	0.826	1.255	0.964	2.395	0.553	0.803	2.622	4.111	2.767	3.714
ANKRD33B	-1.117	0.461	0.014	0.253	0.768	1.885	1.813	0.434	0.151	0.143	2.211	0.872	0.300	0.701	4.172	1.764	2.523
TUBA4A	-1.115	0.462	0.032	0.316	0.768	1.883	0.348	1.597	2.448	0.502	0.418	0.226	0.612	0.390	4.424	0.708	4.415
THBS1	-1.114	0.462	0.012	0.241	9.280	10.393	532.526	401.602	812.157	314.191	555.681	781.963	966.809	956.979	1249.893	1380.719	1406.664
COL21A1	-1.109	0.464	0.039	0.335	0.705	1.814	0.070	3.117	0.568	1.571	0.769	0.243	0.056	0.209	4.503	1.318	2.408
ITPR1	-1.105	0.465	0.000	0.105	1.396	2.501	1.278	1.672	1.133	2.109	2.854	2.060	1.287	1.114	4.898	3.623	5.654
ENPEP	-1.103	0.465	0.007	0.214	0.926	2.030	0.584	1.062	0.520	0.224	2.492	0.867	1.366	0.814	4.885	2.014	2.837
LPP	-1.095	0.468	0.040	0.339	2.863	3.958	8.915	7.440	1.729	4.151	7.521	5.312	9.435	10.895	11.069	11.425	24.022
CAV1	-1.089	0.470	0.045	0.356	6.465	7.554	76.157	110.550	156.537	141.760	140.645	60.870	39.196	53.382	247.586	127.717	206.564
MOK	-1.085	0.472	0.008	0.218	1.150	2.234	4.034	0.719	1.253	1.023	0.844	1.089	0.943	0.992	2.515	4.879	4.043
AGTR1	-1.077	0.474	0.010	0.223	0.735	1.812	0.408	1.993	0.314	0.909	1.083	0.721	0.366	0.141	3.084	1.206	3.811
GRIK5	-1.070	0.476	0.001	0.130	0.199	1.269	0.000	0.074	0.632	0.359	0.116	0.065	0.043	0.018	1.963	0.545	2.054
PLEKHA2	-1.064	0.478	0.028	0.305	2.680	3.744	3.010	11.278	2.145	5.458	4.206	6.755	6.596	8.274	14.673	8.587	15.021
SLC8A1	-1.063	0.479	0.003	0.178	1.626	2.689	2.113	1.427	2.232	1.640	1.043	3.081	4.333	1.870	5.969	4.387	6.139
MYO1D	-1.056	0.481	0.001	0.142	3.108	4.164	6.577	4.800	10.027	8.551	9.908	5.805	7.007	10.091	20.416	13.155	18.014
AIF1L	-1.056	0.481	0.001	0.123	0.078	1.134	0.000	0.018	0.176	0.247	0.033	0.000	0.000	0.000	2.374	0.382	1.269

LRRC2	-1.053	0.482	0.009	0.223	1.888	2.942	1.233	3.717	4.331	3.013	3.987	3.062	1.296	2.369	9.562	4.996	6.162
MET	-1.051	0.482	0.047	0.360	2.557	3.608	5.213	12.197	2.240	5.930	6.603	3.521	3.555	3.990	8.005	7.314	23.234
ACTN4	-1.049	0.483	0.042	0.346	5.963	7.012	57.717	89.716	88.544	58.367	77.395	38.954	43.216	57.462	237.872	53.111	165.408
LYN	-1.040	0.486	0.005	0.201	1.258	2.298	1.050	0.458	0.851	0.963	2.723	1.891	1.775	2.295	4.165	4.204	3.421
RTKN2	-1.035	0.488	0.028	0.305	0.446	1.482	0.083	1.147	1.284	0.230	0.300	0.049	0.083	0.230	4.658	1.346	0.640
CD82	-1.030	0.490	0.002	0.157	1.049	2.079	1.155	0.406	2.224	1.705	1.176	0.474	1.018	0.966	3.395	2.902	3.400
ICAM5	-1.028	0.490	0.004	0.190	0.540	1.568	0.310	0.049	1.662	0.456	0.655	0.066	0.492	0.426	2.179	2.509	1.338
THSD4	-1.026	0.491	0.030	0.308	2.228	3.254	4.439	4.185	1.086	3.753	7.368	3.385	5.842	2.298	7.991	6.207	12.399
LMCD1	-1.025	0.491	0.027	0.303	2.539	3.564	2.795	1.749	8.613	6.635	4.816	5.900	4.896	6.194	9.803	7.542	16.930
UNC5C	-1.023	0.492	0.003	0.160	0.218	1.241	0.065	0.107	0.000	0.587	0.037	0.436	0.153	0.039	0.539	1.323	2.692
FHL1	-1.021	0.493	0.034	0.323	5.412	6.433	41.082	31.108	81.589	33.894	44.817	27.169	27.977	73.207	119.945	51.957	99.811
DOPEY2	-1.021	0.493	0.001	0.123	1.353	2.374	1.093	2.002	1.245	1.468	1.702	1.753	1.568	1.727	6.034	2.244	5.105
CORO6	-1.018	0.494	0.007	0.214	0.843	1.860	0.864	0.463	1.154	0.930	1.082	0.336	0.824	0.858	5.475	1.135	2.461
NEURL1B	-1.017	0.494	0.049	0.362	0.540	1.557	0.074	2.188	0.115	0.855	0.956	0.199	0.166	0.034	4.891	0.812	1.388
ADAM19	-1.014	0.495	0.022	0.282	3.974	4.988	7.115	23.932	12.763	20.480	20.789	13.029	12.905	13.634	32.726	19.206	45.899
ADAMTS7	-1.009	0.497	0.023	0.286	3.165	4.174	7.873	14.641	8.936	11.959	7.878	3.852	6.609	6.153	27.562	11.065	16.076
EMP2	-1.008	0.497	0.004	0.187	1.669	2.676	1.085	3.891	1.992	2.605	2.026	2.066	2.873	1.645	7.387	3.567	5.821
RNF150	-1.007	0.498	0.004	0.194	1.096	2.103	1.661	0.531	2.556	1.061	1.000	0.598	0.984	1.298	3.146	2.334	4.730
RCAN2	-1.006	0.498	0.024	0.289	0.962	1.968	1.566	0.651	0.477	0.819	1.398	0.316	0.626	2.558	2.981	1.317	5.493
CA12	-0.997	0.501	0.015	0.255	3.295	4.292	6.717	5.097	13.194	15.773	9.798	8.772	9.965	5.632	13.704	24.646	18.937
MAP2K3	-0.996	0.501	0.004	0.184	3.324	4.320	12.731	7.305	12.206	7.373	10.496	4.923	9.544	10.187	23.238	15.541	18.889
HBB	-0.994	0.502	0.001	0.123	0.120	1.114	0.000	0.000	0.000	0.000	0.000	0.000	0.949	0.000	1.034	1.411	1.068
TGFB3	-0.990	0.503	0.018	0.264	1.114	2.104	0.631	0.743	1.189	1.963	1.968	0.612	0.676	2.256	4.557	1.483	4.757
ARVCF	-0.988	0.504	0.003	0.159	0.388	1.376	0.312	0.232	0.648	0.249	0.631	0.042	0.040	0.465	3.010	0.848	1.362
RBM20	-0.984	0.505	0.006	0.214	0.285	1.269	0.000	0.446	0.240	0.156	1.023	0.120	0.006	0.028	2.811	0.800	1.042
SHROOM3	-0.983	0.506	0.033	0.322	1.266	2.248	1.513	0.751	1.407	1.119	1.342	0.921	1.426	3.564	3.667	1.485	8.250
TMTC1	-0.976	0.508	0.030	0.310	0.975	1.951	0.520	0.743	0.417	1.678	3.029	0.799	0.647	0.854	4.713	3.662	1.171
NRXN2	-0.975	0.509	0.007	0.214	0.666	1.640	0.450	1.218	1.628	0.812	0.172	0.248	0.454	0.230	2.637	1.349	2.545
ABLIM1	-0.974	0.509	0.029	0.308	1.425	2.400	0.725	1.746	1.327	3.575	2.246	1.914	0.366	3.159	5.831	3.017	4.355
ADAMTS14	-0.972	0.510	0.001	0.123	0.520	1.492	0.230	0.358	0.495	1.039	0.375	0.170	0.752	0.243	2.796	1.507	1.337
CRIM1	-0.970	0.510	0.002	0.159	6.811	7.782	109.222	94.781	125.204	77.757	148.513	103.536	124.442	122.131	274.534	147.538	259.363
JAM2	-0.969	0.511	0.028	0.304	0.995	1.964	1.334	0.376	1.532	0.448	0.464	0.694	1.654	2.213	1.957	1.831	6.088
NATD1	-0.966	0.512	0.003	0.178	2.584	3.549	2.836	3.258	6.485	5.532	6.216	4.278	5.541	7.374	11.664	11.148	9.428
PAG1	-0.965	0.512	0.000	0.029	0.302	1.267	0.173	0.481	0.181	0.370	0.280	0.139	0.111	0.175	1.280	1.090	1.927
TACC1	-0.962	0.513	0.022	0.282	2.554	3.515	2.906	6.195	6.525	2.896	10.803	5.363	3.949	3.611	11.767	7.645	12.544
DAPK1	-0.960	0.514	0.029	0.308	1.665	2.625	1.685	1.633	3.829	1.687	1.346	0.846	2.962	5.506	3.988	6.151	5.584
FAT1	-0.957	0.515	0.000	0.043	4.953	5.911	34.726	28.979	23.212	27.500	30.043	33.044	29.378	34.775	62.851	50.239	65.564
NR2F2	-0.952	0.517	0.032	0.316	4.805	5.757	20.292	34.045	15.730	28.237	47.316	23.712	20.953	37.940	89.613	34.507	48.131
HIP1	-0.951	0.517	0.026	0.294	1.988	2.939	2.310	2.717	2.090	2.550	3.084	3.500	4.621	3.400	14.656	3.011	6.183
MICAL1	-0.945	0.519	0.021	0.281	3.131	4.077	7.860	11.561	8.697	7.850	10.197	5.936	6.190	5.514	30.555	8.640	14.795
TLL1	-0.945	0.519	0.006	0.213	0.030	0.975	0.000	0.000	0.064	0.023	0.000	0.000	0.086	0.000	1.687	0.017	1.779
KRT86	-0.942	0.521	0.020	0.273	0.295	1.237	0.047	0.267	1.970	0.037	0.074	0.005	0.063	0.094	0.779	1.726	1.700
SNX18	-0.931	0.524	0.023	0.284	3.647	4.578	7.285	9.368	14.971	14.363	18.975	10.931	8.663	11.511	41.231	17.466	16.489
ALPK3	-0.927	0.526	0.006	0.208	0.452	1.379	0.139	0.949	0.969	0.364	0.215	0.148	0.125	0.310	0.960	1.327	2.858
HK2	-0.926	0.526	0.004	0.180	3.862	4.788	9.150	14.169	17.342	11.592	20.751	11.444	12.730	14.107	33.081	19.815	28.703
ARID5B	-0.924	0.527	0.002	0.157	4.840	5.764	23.107	20.481	27.877	27.252	36.715	28.159	25.965	35.090	70.772	37.977	56.351
SERPINI1	-0.923	0.527	0.020	0.274	1.375	2.299	2.235	2.079	2.589	1.139	1.233	1.359	1.065	1.466	2.434	2.556	8.752
DAB2IP	-0.922	0.528	0.025	0.292	1.963	2.885	2.079	3.658	4.788	4.025	4.402	2.297	1.359	2.052	11.021	5.031	4.562
SMTN	-0.922	0.528	0.022	0.283	2.275	3.197	3.974	5.221	4.723	4.133	3.189	3.478	2.946	3.479	8.678	3.601	16.312
MYH9	-0.922	0.528	0.008	0.219	7.814	8.736	190.425	290.459	214.838	214.278	323.087	191.763	160.318	250.776	589.915	269.700	483.172
APCDD1	-0.921	0.528	0.007	0.214	0.087	1.008	0.000	0.024	0.449	0.075	0.015	0.000	0.000	0.000	2.459	0.199	0.962
EPHB6	-0.921	0.528	0.011	0.231	0.885	1.805	0.475	1.289	1.255	1.933	1.083	0.341	0.178	0.839	3.179	2.768	1.712
PDLIM1	-0.918	0.529	0.020	0.273	4.399	5.317	16.301	19.980	31.558	18.848	34.138	18.673	15.758	13.462	57.808	26.152	38.676

WNT5B	-0.914	0.531	0.006	0.214	0.873	1.787	0.635	1.219	2.315	0.857	0.824	0.545	0.492	0.347	1.887	2.470	3.101
CABP1	-0.914	0.531	0.046	0.356	0.903	1.817	1.255	0.220	3.291	0.557	1.149	0.834	0.540	0.341	2.731	1.254	4.201
KIRREL3	-0.912	0.531	0.025	0.292	1.153	2.066	1.330	0.535	0.884	0.442	0.757	2.183	2.656	2.019	2.441	2.522	5.055
MYL4	-0.910	0.532	0.039	0.336	0.254	1.164	0.083	0.367	0.629	0.287	0.218	0.052	0.027	0.000	4.332	0.374	0.534
CREB5	-0.908	0.533	0.001	0.139	0.657	1.565	0.536	0.526	0.945	0.517	0.259	0.634	0.484	0.812	1.107	1.862	3.295
HSPB8	-0.905	0.534	0.023	0.286	3.183	4.088	7.316	6.198	9.503	4.752	10.251	7.112	9.616	12.200	26.203	8.856	17.351
PRDM16	-0.905	0.534	0.010	0.228	0.396	1.301	0.215	1.332	0.259	0.191	0.163	0.235	0.128	0.305	2.393	0.538	1.867
LRP5	-0.905	0.534	0.037	0.331	3.078	3.983	5.597	14.847	6.328	8.888	9.691	6.635	8.744	3.286	17.961	9.592	18.679
DUSP5	-0.903	0.535	0.006	0.205	1.417	2.321	2.268	1.597	1.565	1.991	1.604	1.220	1.762	1.492	5.184	1.837	6.106
AL365205.1	-0.902	0.535	0.038	0.334	1.399	2.301	1.190	1.168	1.576	1.213	5.211	3.094	0.750	0.940	3.856	4.180	3.754
SEMA6D	-0.897	0.537	0.005	0.196	0.446	1.343	0.567	0.331	0.173	0.244	0.667	0.305	0.279	0.401	0.674	1.311	3.219
MYH3	-0.897	0.537	0.011	0.236	0.533	1.430	0.245	0.532	0.333	0.158	0.890	0.277	0.444	0.878	2.076	0.537	3.139
TNFSF12-TNF	-0.895	0.538	0.006	0.213	0.000	0.895	0.000	0.000	0.000	0.000	0.000	0.000	0.000	0.000	1.761	1.331	0.000
A4GALT	-0.893	0.539	0.022	0.282	2.471	3.364	3.968	7.492	7.787	2.922	4.786	2.682	4.621	4.136	14.610	8.256	6.553
WFS1	-0.892	0.539	0.035	0.324	2.837	3.730	4.315	4.460	12.963	2.783	8.871	5.379	7.220	7.572	11.349	11.286	14.385
ARL8B	-0.890	0.539	0.025	0.292	2.590	3.480	4.317	5.030	4.698	5.406	4.514	4.743	5.148	6.561	6.118	23.673	6.910
NT5DC3	-0.885	0.542	0.001	0.123	1.052	1.936	1.194	1.159	1.222	1.127	1.279	0.558	0.914	1.240	2.896	1.761	4.209
SLC4A3	-0.883	0.542	0.012	0.240	0.995	1.878	0.969	0.440	0.716	0.410	2.136	0.886	1.453	1.501	3.610	1.553	3.221
ETS2	-0.881	0.543	0.017	0.264	1.899	2.780	1.529	3.694	2.123	3.356	4.709	2.883	1.933	2.556	9.954	5.185	3.782
DMPK	-0.881	0.543	0.035	0.324	2.895	3.775	6.678	5.217	13.492	5.596	6.691	3.288	6.199	7.644	21.271	8.883	10.665
TPST2	-0.877	0.544	0.027	0.301	2.339	3.216	2.787	2.004	7.323	5.498	4.132	3.522	3.628	5.513	13.337	5.991	7.011
RASAL2	-0.873	0.546	0.008	0.219	1.746	2.619	2.062	1.691	1.195	3.634	2.101	2.452	4.589	2.193	5.526	4.750	5.181
MEF2C	-0.873	0.546	0.014	0.252	0.770	1.642	0.502	1.228	0.690	1.026	1.082	0.352	0.365	0.621	4.515	1.085	1.645
DOK5	-0.872	0.546	0.001	0.106	3.033	3.905	7.107	6.240	7.696	5.722	9.733	9.498	6.406	6.018	16.820	12.412	13.054
NEDD9	-0.872	0.546	0.011	0.236	1.854	2.726	1.394	2.865	4.526	1.836	3.537	2.559	2.325	2.740	7.231	3.433	6.929
ENO2	-0.869	0.548	0.025	0.290	3.669	4.538	11.836	6.993	11.568	9.584	13.805	8.888	24.122	12.644	16.809	21.031	30.949
IFFO2	-0.866	0.549	0.000	0.100	2.137	3.003	3.083	3.564	3.012	2.608	3.555	2.971	4.243	4.489	6.335	5.997	9.048
LRIG1	-0.866	0.549	0.024	0.287	1.718	2.583	1.928	3.158	1.958	2.158	2.920	3.753	2.207	1.015	2.514	5.782	8.036
ENAH	-0.857	0.552	0.006	0.213	3.735	4.593	11.883	13.506	11.803	12.275	15.558	10.076	10.359	13.974	23.509	14.189	36.730
CACNA1C	-0.857	0.552	0.002	0.157	1.067	1.924	0.710	0.560	0.707	1.339	1.551	1.566	1.556	1.082	3.126	2.044	3.351
CELSR2	-0.856	0.552	0.002	0.157	0.470	1.327	0.208	0.447	0.488	0.503	0.538	0.124	0.263	0.589	2.026	0.666	2.130
KLHL23	-0.854	0.553	0.001	0.107	0.169	1.024	0.031	0.447	0.134	0.038	0.110	0.162	0.083	0.044	1.288	0.443	1.546
HAS3	-0.852	0.554	0.002	0.157	0.579	1.430	0.452	0.426	0.798	0.778	0.248	0.328	0.253	0.802	2.553	0.938	1.844
FLNA	-0.850	0.555	0.015	0.255	7.794	8.644	184.224	274.100	189.297	216.199	281.878	209.428	171.316	270.898	475.657	227.450	586.641
PTGER2	-0.849	0.555	0.005	0.205	0.524	1.373	0.600	0.803	0.287	0.378	0.650	0.036	0.710	0.224	1.959	0.727	2.402
COL4A1	-0.848	0.555	0.010	0.228	8.604	9.453	376.210	694.348	412.106	345.609	338.103	287.104	357.000	399.250	748.014	507.536	902.031
NXPH3	-0.848	0.555	0.002	0.157	0.211	1.059	0.093	0.085	0.359	0.078	0.083	0.000	0.189	0.438	0.833	0.577	2.130
PCGF5	-0.846	0.557	0.003	0.171	3.447	4.292	10.410	7.502	13.024	7.391	10.855	10.187	9.436	11.631	26.736	14.570	16.409
ADCY9	-0.846	0.557	0.000	0.105	2.372	3.218	3.767	4.452	3.492	4.010	5.434	4.067	4.572	3.864	11.085	6.133	8.345
LCLAT1	-0.844	0.557	0.000	0.105	1.667	2.510	1.624	2.193	2.824	1.598	2.647	1.979	2.216	2.547	5.534	3.448	5.365
ARNTL2	-0.840	0.558	0.007	0.214	1.113	1.953	2.610	1.384	1.868	0.962	0.833	0.869	0.691	0.707	2.429	2.928	3.313
SPCS3	-0.839	0.559	0.013	0.243	4.745	5.585	32.286	17.866	26.241	21.362	30.122	18.274	23.566	46.550	52.688	44.717	44.045
NOG	-0.838	0.559	0.032	0.317	0.502	1.340	0.095	0.203	0.570	0.400	0.439	0.324	0.584	0.849	0.486	1.093	4.212
NXN	-0.835	0.560	0.022	0.281	1.654	2.489	2.370	0.927	3.574	1.206	2.747	1.285	2.846	3.456	4.893	3.715	5.369
NFIB	-0.834	0.561	0.032	0.317	2.309	3.143	2.840	2.723	1.859	4.234	5.152	6.181	4.127	6.493	5.489	11.480	7.512
SLC29A2	-0.833	0.561	0.007	0.214	0.193	1.026	0.000	0.098	0.130	0.291	0.613	0.082	0.041	0.000	2.256	0.434	0.809
MEF2A	-0.827	0.564	0.014	0.250	2.893	3.720	5.070	7.316	6.035	4.566	7.616	4.878	5.484	13.255	14.095	12.053	10.614
SRPX	-0.826	0.564	0.005	0.196	4.157	4.983	16.395	17.104	16.064	21.498	20.601	18.947	12.123	14.032	45.689	24.976	25.087
CAV2	-0.825	0.564	0.005	0.201	4.138	4.963	14.631	15.718	28.836	16.423	19.050	14.198	13.486	14.367	35.179	23.658	33.000
FRY	-0.815	0.569	0.046	0.356	1.627	2.441	1.896	1.183	4.969	1.917	2.244	1.090	1.219	3.989	6.088	3.572	3.944
FRMPD4	-0.812	0.569	0.003	0.180	0.991	1.804	1.131	1.069	1.062	0.507	1.596	0.760	1.427	0.607	3.713	2.263	1.768
ADAMTS8	-0.809	0.571	0.012	0.243	0.049	0.858	0.000	0.000	0.000	0.286	0.019	0.000	0.000	0.000	0.068	0.775	2.139
PDK3	-0.809	0.571	0.006	0.213	0.959	1.767	0.736	1.082	0.744	0.594	1.013	0.853	1.655	1.044	2.062	1.438	4.283

SDC4	-0.803	0.573	0.008	0.219	5.033	5.836	42.278	22.209	47.208	24.980	31.098	26.445	33.154	33.892	57.764	44.675	68.481
ETV7	-0.799	0.575	0.031	0.314	0.335	1.135	0.281	0.141	0.328	0.457	0.221	0.151	0.221	0.323	3.706	0.584	0.421
EPPK1	-0.796	0.576	0.034	0.324	0.375	1.171	0.107	0.424	0.249	0.131	0.350	0.093	0.155	1.110	1.581	0.218	2.629
EHD1	-0.793	0.577	0.049	0.362	2.889	3.682	7.606	9.238	7.097	7.867	9.773	3.452	3.861	5.135	19.005	11.434	7.492
MRGPRF	-0.790	0.578	0.013	0.243	3.032	3.822	7.616	7.753	7.600	7.869	8.170	5.251	7.466	6.177	22.517	7.720	12.808
LINC00672	-0.789	0.579	0.037	0.330	0.906	1.695	0.750	0.548	1.371	0.732	1.350	0.607	0.638	1.213	4.222	0.619	3.017
GJA5	-0.777	0.583	0.022	0.283	0.503	1.280	0.000	1.025	0.897	1.089	0.210	0.294	0.055	0.226	2.230	1.095	1.117
PRKAA2	-0.776	0.584	0.011	0.234	0.875	1.650	0.293	0.480	0.879	0.491	1.203	0.989	1.136	1.546	2.528	1.251	2.893
RNF141	-0.776	0.584	0.025	0.292	3.450	4.226	9.354	9.899	18.282	9.859	11.720	6.725	7.172	9.731	26.074	12.761	16.577
LIMD1	-0.769	0.587	0.001	0.105	1.694	2.463	1.779	2.874	2.806	2.122	2.324	2.014	2.062	2.055	6.110	3.464	4.282
FAM89A	-0.768	0.587	0.048	0.361	0.771	1.539	0.131	0.371	0.163	0.479	1.804	0.690	0.881	2.017	2.772	1.608	1.494
VGLL3	-0.765	0.588	0.022	0.283	4.578	5.343	21.073	14.295	19.188	19.093	39.841	24.912	23.140	29.278	33.198	35.934	51.972
BACH1	-0.760	0.590	0.014	0.250	3.322	4.082	7.619	8.912	17.088	11.453	8.568	7.761	5.970	7.892	15.774	15.687	16.358
FXYD1	-0.757	0.592	0.019	0.269	0.634	1.391	0.089	0.174	1.329	0.706	0.440	0.239	0.610	1.308	2.017	1.128	1.810
SLMAP	-0.752	0.594	0.002	0.157	2.076	2.829	3.861	2.365	3.296	3.209	4.113	2.650	2.865	3.688	7.650	4.205	6.961
CD9	-0.752	0.594	0.045	0.356	3.787	4.540	19.113	10.869	20.614	8.799	15.793	8.010	9.725	15.097	32.260	19.753	17.226
SNTA1	-0.752	0.594	0.045	0.356	2.876	3.628	4.042	5.446	7.804	5.177	7.928	5.699	8.619	7.279	21.110	6.168	10.918
ZFHX3	-0.750	0.594	0.004	0.180	1.952	2.703	2.534	4.501	3.505	3.340	2.950	2.198	2.349	2.130	7.168	4.680	4.949
PDZRN3	-0.747	0.596	0.035	0.324	1.538	2.285	2.190	1.215	1.082	4.233	2.397	2.250	2.221	0.852	3.623	3.228	4.927
DKK3	-0.746	0.596	0.044	0.354	4.648	5.394	17.036	17.311	40.604	23.745	22.051	20.495	29.725	29.119	40.381	26.253	64.859
ARHGEF7	-0.744	0.597	0.002	0.159	2.577	3.321	4.713	5.233	4.277	5.731	5.724	4.421	4.767	5.051	13.925	6.987	7.371
RABGAP1	-0.742	0.598	0.001	0.143	3.116	3.858	6.737	8.250	8.147	7.591	8.908	7.460	6.560	7.959	19.003	9.671	13.280
DUSP8	-0.739	0.599	0.017	0.264	0.536	1.275	0.218	0.266	1.458	0.328	0.599	0.034	0.204	0.952	1.748	1.267	1.278
ARHGEF17	-0.737	0.600	0.016	0.258	3.408	4.146	7.786	9.378	9.625	9.388	12.552	7.766	9.638	11.686	27.049	10.233	16.595
DDAH1	-0.731	0.603	0.038	0.334	5.650	6.381	56.895	36.326	55.855	31.414	60.683	42.305	44.782	82.100	75.273	62.513	118.546
GMPR	-0.729	0.603	0.040	0.341	0.802	1.531	0.783	0.599	0.982	1.219	1.679	0.351	0.505	0.247	3.681	1.700	0.909
FIBCD1	-0.729	0.603	0.041	0.342	1.156	1.885	0.597	1.336	0.751	1.657	1.695	1.710	2.455	0.386	2.120	2.083	4.236
NKX2-5	-0.728	0.604	0.007	0.214	0.000	0.728	0.000	0.000	0.000	0.000	0.000	0.000	0.000	0.000	0.000	0.852	1.454
MSRB1	-0.726	0.605	0.042	0.347	2.437	3.163	4.189	3.857	7.596	4.886	5.742	3.087	2.519	4.980	12.302	4.894	8.167
OBSL1	-0.725	0.605	0.020	0.275	2.325	3.050	2.966	4.052	3.950	4.290	6.126	3.583	3.846	3.784	12.644	4.438	6.661
CPEB2	-0.725	0.605	0.012	0.242	1.500	2.225	1.518	2.457	1.189	1.758	2.127	2.526	1.342	2.011	4.404	2.030	5.238
COL4A2	-0.725	0.605	0.009	0.223	8.228	8.953	298.777	459.277	338.197	265.413	281.865	211.323	284.546	304.370	532.459	380.633	596.978
PDE5A	-0.724	0.605	0.011	0.234	2.856	3.580	4.911	9.115	6.332	6.334	8.311	3.898	5.887	6.468	8.541	10.780	14.217
DDO	-0.719	0.608	0.026	0.299	0.286	1.005	0.064	0.045	0.190	0.889	0.402	0.120	0.101	0.130	2.381	0.463	0.633
GBP2	-0.719	0.608	0.014	0.250	1.740	2.458	1.424	1.613	2.209	2.870	3.608	2.572	1.866	3.168	6.581	3.527	3.835
PLXNB1	-0.718	0.608	0.008	0.219	1.845	2.563	2.562	1.783	2.633	2.242	3.546	2.506	2.360	3.445	7.854	3.888	3.767
AP1S2	-0.714	0.610	0.010	0.228	2.281	2.995	3.443	5.059	4.316	3.951	5.884	2.439	3.239	3.378	9.284	5.323	6.790
INPP4B	-0.713	0.610	0.006	0.205	0.564	1.277	0.543	1.042	0.253	0.655	0.548	0.308	0.332	0.294	2.259	0.805	1.420
PRPH	-0.713	0.610	0.002	0.159	0.032	0.745	0.000	0.000	0.105	0.081	0.000	0.000	0.000	0.000	0.993	0.105	1.139
BIRC3	-0.709	0.612	0.000	0.004	0.171	0.881	0.134	0.045	0.110	0.134	0.141	0.141	0.198	0.111	0.693	1.015	0.830
MBNL1	-0.708	0.612	0.002	0.157	4.479	5.188	20.344	21.960	18.295	22.092	29.272	19.406	17.569	23.456	37.994	27.826	42.063
SNTB2	-0.706	0.613	0.006	0.205	2.984	3.690	4.997	7.380	5.305	8.067	10.006	6.041	6.632	8.026	13.518	9.464	13.149
TPM2	-0.703	0.614	0.023	0.284	7.213	7.916	164.910	146.768	159.619	145.064	181.586	131.309	105.136	158.357	299.658	141.098	328.900
INA	-0.694	0.618	0.035	0.324	0.657	1.351	0.568	1.023	1.236	0.408	1.210	0.064	0.162	0.397	2.436	0.943	1.486
PVR	-0.694	0.618	0.035	0.324	4.044	4.738	17.697	20.433	11.831	8.240	20.470	14.905	16.678	18.066	20.868	22.677	35.677
ZNF608	-0.694	0.618	0.042	0.346	0.640	1.334	0.324	1.325	0.372	0.770	0.946	0.361	0.329	0.324	3.405	0.934	0.881
UGCG	-0.692	0.619	0.026	0.295	5.102	5.795	45.457	34.221	46.246	26.965	37.438	24.280	42.599	20.186	52.531	56.251	54.812
GLRB	-0.692	0.619	0.004	0.187	1.463	2.155	1.634	2.075	1.834	1.686	1.658	2.606	1.491	1.262	2.388	3.306	5.052
MFSD6	-0.691	0.619	0.003	0.178	1.120	1.811	0.892	0.793	1.070	1.196	1.996	1.687	1.144	0.874	3.258	2.222	2.150
DMTN	-0.690	0.620	0.001	0.105	0.160	0.850	0.071	0.096	0.251	0.074	0.091	0.043	0.161	0.164	1.386	0.412	0.739
RIMS3	-0.690	0.620	0.001	0.105	0.440	1.130	0.035	0.426	0.455	0.437	0.292	0.394	0.346	0.538	1.644	1.124	0.868
PDIA5	-0.688	0.621	0.022	0.283	3.780	4.468	19.102	11.441	17.551	12.569	10.426	12.368	11.065	9.956	31.236	15.420	19.485
ERMN	-0.686	0.621	0.017	0.264	0.244	0.930	0.480	0.022	1.065	0.056	0.000	0.029	0.081	0.052	0.566	1.011	1.195

PM20D2	-0.681	0.624	0.001	0.123	0.727	1.408	0.216	0.463	0.516	0.760	0.939	0.736	0.793	0.967	1.785	1.494	1.689
SHROOM2	-0.679	0.624	0.006	0.208	0.089	0.769	0.000	0.093	0.125	0.011	0.106	0.000	0.083	0.101	0.188	0.591	1.615
ABCA3	-0.678	0.625	0.006	0.213	0.460	1.138	0.325	0.685	1.092	0.289	0.321	0.085	0.155	0.288	1.106	1.020	1.508
BCAS4	-0.678	0.625	0.000	0.028	0.429	1.107	0.284	0.262	0.317	0.379	0.335	0.561	0.233	0.430	1.054	1.031	1.395
MKX	-0.675	0.626	0.013	0.243	0.314	0.989	0.039	0.185	0.020	0.245	0.396	0.286	0.507	0.348	0.451	0.742	2.096
PLEKHG3	-0.675	0.627	0.011	0.236	0.301	0.976	0.136	0.380	0.457	0.282	0.270	0.028	0.050	0.325	1.907	0.314	0.991
WDR1	-0.674	0.627	0.022	0.283	5.645	6.320	50.160	60.786	58.536	46.931	58.565	38.544	32.885	53.646	113.504	56.242	76.730
CDV3	-0.671	0.628	0.019	0.269	4.439	5.111	14.025	17.115	30.896	22.832	16.067	22.423	24.996	21.846	40.905	26.354	34.985
PIK3R1	-0.670	0.628	0.007	0.214	1.688	2.358	1.544	1.819	2.187	2.140	3.025	1.451	2.710	3.412	3.538	4.199	4.708
PGAM2	-0.669	0.629	0.035	0.324	0.041	0.710	0.000	0.000	0.000	0.103	0.000	0.000	0.137	0.000	2.174	0.129	0.221
NXPH4	-0.667	0.630	0.042	0.346	2.928	3.596	4.439	8.843	12.536	5.678	6.082	6.342	7.443	4.311	10.687	9.168	13.864
TSPAN9	-0.667	0.630	0.005	0.200	2.676	3.343	4.112	3.976	6.923	4.722	6.480	5.384	5.567	6.713	7.303	9.406	11.096
FRMD4A	-0.666	0.630	0.026	0.298	2.944	3.610	8.322	9.618	7.566	6.681	7.626	5.165	5.076	4.836	15.733	7.370	12.004
PDCD6IP	-0.665	0.631	0.000	0.084	3.828	4.494	11.493	11.800	16.459	12.073	11.864	14.943	14.030	13.746	21.599	20.294	22.764
DNAJB4	-0.665	0.631	0.029	0.308	4.551	5.216	26.981	22.697	38.830	22.147	22.916	17.483	17.549	17.155	28.284	35.069	47.599
STAT4	-0.665	0.631	0.017	0.262	0.913	1.578	0.755	1.210	1.716	0.943	0.687	0.802	0.414	0.796	3.251	1.202	1.842
PKIG	-0.664	0.631	0.028	0.305	3.512	4.176	8.594	12.656	11.196	13.507	14.743	6.363	9.311	9.354	22.505	12.260	17.940
LACTB	-0.659	0.633	0.015	0.255	3.174	3.833	7.672	7.567	13.125	7.049	7.785	6.503	8.403	7.409	19.003	9.683	12.535
NEXN	-0.653	0.636	0.029	0.308	4.771	5.424	24.302	24.473	42.628	23.811	28.238	24.068	19.465	28.489	61.039	29.296	41.120
C4A	-0.653	0.636	0.019	0.269	0.887	1.540	0.355	0.834	0.362	1.165	1.775	0.700	0.843	1.144	2.578	1.871	1.392
ENPP4	-0.652	0.636	0.049	0.362	0.590	1.243	0.323	0.675	0.273	0.428	0.507	0.557	0.378	1.027	0.449	1.111	3.332
WWP2	-0.651	0.637	0.008	0.217	1.935	2.586	2.090	3.273	3.973	2.780	3.563	2.223	2.525	2.548	7.082	3.841	4.528
JAZF1	-0.644	0.640	0.009	0.223	2.912	3.556	5.617	5.290	8.920	5.216	5.443	8.738	7.226	6.769	10.892	8.373	13.591
DIAPH2	-0.644	0.640	0.006	0.214	1.362	2.006	1.432	1.353	0.782	1.348	2.403	1.988	1.767	1.834	2.730	2.645	3.768
CALD1	-0.643	0.640	0.038	0.333	7.656	8.300	157.256	198.973	248.702	191.446	253.962	144.278	157.970	305.230	392.178	228.607	345.614
OR51E2	-0.637	0.643	0.011	0.236	0.218	0.855	0.078	0.033	0.455	0.589	0.146	0.086	0.000	0.047	0.505	0.545	1.544
SATB1	-0.635	0.644	0.009	0.223	0.777	1.412	0.670	0.500	0.254	0.924	0.542	0.767	1.326	0.943	1.673	1.119	2.329
TLE4	-0.634	0.645	0.015	0.256	1.544	2.178	2.011	3.124	2.985	1.454	1.991	1.337	1.741	1.246	4.262	2.776	3.659
ANKS1B	-0.630	0.646	0.009	0.223	0.129	0.759	0.008	0.213	0.163	0.314	0.041	0.014	0.012	0.024	1.579	0.438	0.306
OPTN	-0.629	0.646	0.005	0.201	4.650	5.279	21.017	18.382	31.191	23.823	23.898	30.803	20.439	26.208	42.635	39.519	32.100
MYO18A	-0.628	0.647	0.029	0.308	1.816	2.444	2.796	2.274	3.395	1.701	2.328	2.371	2.707	2.842	8.016	2.787	3.716
ANKRD13A	-0.627	0.647	0.016	0.258	4.051	4.678	15.313	12.385	12.009	13.054	27.171	16.714	15.891	15.923	23.471	25.977	24.417
ACTN1	-0.627	0.648	0.009	0.223	5.730	6.357	44.692	59.127	54.314	46.419	55.012	55.598	41.389	64.156	85.218	58.265	106.801
SLCO3A1	-0.627	0.648	0.017	0.262	1.277	1.904	1.356	1.093	1.153	1.019	1.221	1.022	2.266	2.780	2.899	3.147	2.239
HSDL2	-0.626	0.648	0.010	0.223	3.943	4.568	15.387	16.530	14.957	13.704	17.372	13.217	14.367	10.548	32.013	22.007	16.586
TRAM2	-0.626	0.648	0.000	0.062	6.001	6.627	58.369	64.553	73.762	65.356	68.420	54.400	60.274	61.373	110.485	93.226	90.939
ENPP1	-0.624	0.649	0.009	0.221	0.439	1.063	0.162	0.761	0.344	0.331	0.398	0.345	0.462	0.132	1.666	0.468	1.329
OLFML2A	-0.624	0.649	0.014	0.252	0.259	0.883	0.123	0.089	0.618	0.141	0.321	0.082	0.218	0.067	1.392	0.224	1.141
ZNF365	-0.622	0.650	0.008	0.219	0.229	0.851	0.190	0.204	0.682	0.065	0.165	0.017	0.075	0.091	1.376	0.780	0.389
PDE1C	-0.621	0.650	0.049	0.362	3.531	4.153	8.298	7.195	9.093	9.559	12.731	12.599	13.917	13.126	24.126	10.195	19.002
SLC4A7	-0.621	0.650	0.012	0.239	3.927	4.548	20.763	11.031	11.031	13.614	11.986	15.303	18.247	14.302	18.682	24.211	24.815
FCHSD2	-0.620	0.651	0.024	0.287	2.767	3.387	4.887	6.000	6.261	7.365	6.814	5.193	4.621	5.763	15.741	6.390	8.258
LYST	-0.619	0.651	0.001	0.107	1.091	1.710	0.959	1.186	1.159	1.579	1.143	1.001	1.085	0.988	3.113	1.725	2.126
RUBCNL	-0.615	0.653	0.001	0.123	0.058	0.673	0.000	0.046	0.093	0.000	0.057	0.049	0.066	0.022	0.874	0.165	0.855
ROCK2	-0.612	0.654	0.023	0.285	3.749	4.361	10.665	13.664	9.071	12.433	16.755	11.884	12.747	13.678	30.143	14.414	17.081
FLNC	-0.611	0.655	0.011	0.236	6.000	6.612	72.374	50.995	54.436	52.477	62.618	61.685	72.826	83.744	76.337	92.672	128.095
JPT2	-0.611	0.655	0.047	0.358	1.826	2.436	3.794	3.552	1.509	3.760	3.035	1.262	2.233	2.238	5.152	4.863	3.395
SELPLG	-0.607	0.657	0.010	0.228	1.034	1.641	0.925	1.181	1.281	1.477	1.152	1.221	0.489	0.826	2.343	1.304	2.935
B3GNT2	-0.607	0.657	0.022	0.282	2.875	3.481	5.905	6.713	10.401	6.018	7.042	5.254	4.538	6.053	13.986	8.123	9.185
GLIS1	-0.606	0.657	0.002	0.158	0.256	0.863	0.029	0.120	0.491	0.498	0.028	0.123	0.279	0.091	0.783	0.636	1.062
SH3BP4	-0.606	0.657	0.025	0.293	3.827	4.433	15.716	9.113	13.227	14.243	11.300	13.092	21.585	10.444	25.032	18.954	18.392
C4B	-0.605	0.657	0.021	0.277	0.863	1.468	0.408	0.781	0.406	1.040	1.775	0.699	0.706	1.068	2.455	1.702	1.267
EPB41L1	-0.604	0.658	0.017	0.264	1.785	2.389	2.284	2.597	1.895	2.107	3.040	2.390	2.241	3.213	6.745	2.604	4.146

NEDD4L	-0.601	0.659	0.001	0.106	0.580	1.181	0.623	0.397	0.389	0.285	0.768	0.429	0.419	0.720	1.020	1.194	1.632
TOM1L1	-0.599	0.660	0.015	0.253	0.909	1.508	0.697	1.379	1.104	1.194	1.255	0.635	0.428	0.576	2.687	1.662	1.342
RELL1	-0.598	0.661	0.007	0.214	1.941	2.539	2.061	3.348	2.722	2.395	3.416	3.231	3.868	2.084	5.567	3.738	5.310
PRKAR1B	-0.597	0.661	0.048	0.361	1.918	2.515	2.422	2.742	4.002	2.887	4.196	1.917	2.459	2.184	7.790	2.807	4.578
AVPI1	-0.597	0.661	0.021	0.277	2.686	3.283	4.549	4.018	6.291	5.744	5.048	5.769	7.830	4.950	13.178	6.929	7.201
PGM2	-0.593	0.663	0.002	0.159	3.329	3.922	8.085	10.389	10.098	7.803	11.060	8.554	8.610	8.293	17.934	11.228	14.054
FEM1B	-0.593	0.663	0.019	0.269	3.354	3.948	8.891	12.368	6.967	10.144	12.180	6.829	8.139	9.812	16.249	10.771	17.093
WNT16	-0.593	0.663	0.042	0.346	0.096	0.688	0.000	0.000	0.571	0.082	0.000	0.000	0.000	0.000	1.726	0.264	0.214
WWC3	-0.592	0.663	0.005	0.197	2.997	3.590	6.654	8.486	9.011	5.911	6.509	5.567	7.654	6.716	12.467	12.628	8.510
IDH2	-0.592	0.663	0.037	0.329	3.664	4.257	9.383	13.701	12.589	13.289	12.048	10.839	11.149	11.012	22.575	10.054	25.796
C8orf58	-0.589	0.665	0.003	0.178	2.353	2.942	2.863	4.303	5.524	3.887	4.654	4.212	3.730	4.103	8.261	6.046	5.956
FAS	-0.587	0.666	0.049	0.362	3.259	3.846	8.752	6.133	10.406	9.276	6.810	5.945	11.953	11.286	19.579	10.076	12.040
MSTN	-0.587	0.666	0.000	0.105	0.134	0.721	0.000	0.034	0.134	0.126	0.161	0.103	0.115	0.114	0.373	0.568	1.079
CORO1C	-0.585	0.666	0.035	0.324	4.840	5.425	34.482	27.799	34.755	23.149	34.161	21.526	22.665	26.304	47.973	27.529	55.745

Table S4. The differentially expressed age-related genes in the obese hASCs and lean hASCs

HSA04213 Longevity regulating pathway-multiple species related genes

Gene_Name	log2FC	Fold_Change	p_value	q_value	Obese_FPKM	Control_FPKM	obese 1	obese 2	obese 3	obese 4	obese 5	obese 6	obese 7	obese 8	lean 1	lean 2	lean 3
CRYAB	-1.617	0.326	0.014	0.251	3.770	5.387	11.937	16.846	28.245	10.389	13.497	7.137	7.649	14.279	84.860	15.106	52.020
PRKAG2	-1.505	0.352	0.003	0.160	2.384	3.888	3.206	3.957	6.873	3.705	6.768	3.679	2.493	4.610	24.343	6.919	15.180
ADCY9	-0.846	0.557	0.000	0.105	2.372	3.218	3.767	4.452	3.492	4.010	5.434	4.067	4.572	3.864	11.085	6.133	8.345
PRKAA2	-0.776	0.584	0.011	0.234	0.875	1.650	0.293	0.480	0.879	0.491	1.203	0.989	1.136	1.546	2.528	1.251	2.893
PIK3R1	-0.670	0.628	0.007	0.214	1.688	2.358	1.544	1.819	2.187	2.140	3.025	1.451	2.710	3.412	3.538	4.199	4.708
ADCY3	-0.490	0.712	0.016	0.260	2.579	3.069	4.594	4.082	5.504	4.785	5.628	4.504	6.272	4.712	10.217	5.323	7.330

HSA04115 p53 signalling pathway related genes

Gene_Name	log2FC	Fold_Change	p_value	q_value	Obese_FPKM	Control_FPKM	obese 1	obese 2	obese 3	obese 4	obese 5	obese 6	obese 7	obese 8	lean 1	lean 2	lean 3
IGFBP3	2.499	5.655	0.024	0.289	8.370	5.871	186.949	197.847	405.252	371.571	385.995	442.859	296.844	496.046	5.621	168.253	177.850
SESN3	1.282	2.432	0.032	0.317	3.802	2.520	26.877	9.909	9.482	12.114	8.299	16.798	8.490	20.840	1.326	8.074	7.937
TP53I3	0.844	1.795	0.013	0.243	3.911	3.067	17.885	11.572	10.135	12.165	12.759	21.810	11.104	18.838	4.850	9.483	8.606
CYCS	0.453	1.368	0.021	0.279	4.425	3.973	23.588	24.300	17.381	22.736	18.990	19.993	17.601	20.420	10.528	17.529	17.114
CHEK2	0.403	1.322	0.036	0.327	1.703	1.300	2.718	3.289	2.549	2.200	2.036	1.914	2.176	1.474	1.323	1.984	1.153
SIAH1	0.394	1.314	0.013	0.244	2.193	1.799	2.927	2.904	4.479	3.533	3.189	4.257	4.177	3.405	2.216	2.989	2.287
PPM1D	0.389	1.309	0.030	0.308	2.437	2.049	3.521	5.032	4.784	4.739	4.596	4.298	3.711	4.855	2.489	4.578	2.639
BAX	0.371	1.293	0.033	0.322	4.452	4.081	20.618	20.852	28.803	20.420	16.089	18.872	21.395	21.962	14.725	18.779	14.585
SESN1	0.282	1.216	0.051	0.365	2.634	2.352	4.838	4.186	5.663	5.365	4.710	5.750	5.102	6.294	3.497	5.285	3.704

age-related genes

Gene_Name	log2FC	Fold_Change	p_value	q_value	Obese_FPKM	Control_FPKM	obese 1	obese 2	obese 3	obese 4	obese 5	obese 6	obese 7	obese 8	lean 1	lean 2	lean 3
IGFBP3	2.499	5.655	0.024	0.289	8.370	5.871	186.949	197.847	405.252	371.571	385.995	442.859	296.844	496.046	5.621	168.253	177.850
CDKN2B	2.015	4.043	0.004	0.196	4.636	2.621	23.985	14.637	34.949	23.294	15.020	21.691	21.752	50.821	1.327	12.525	6.394
IL1R1	1.686	3.217	0.014	0.249	5.621	3.935	46.871	37.265	51.384	93.204	60.223	37.231	38.246	40.420	6.414	49.803	8.505
BMP6	1.629	3.092	0.033	0.323	3.439	1.810	20.792	3.665	16.901	6.421	8.813	5.723	8.691	21.145	0.484	7.967	2.242
MMP16	1.540	2.908	0.015	0.255	2.824	1.284	8.000	11.254	8.606	8.959	1.261	4.687	7.041	4.791	0.449	3.068	1.448
FZD8	1.533	2.894	0.001	0.107	2.661	1.128	4.055	5.612	6.820	7.032	3.220	5.377	5.968	5.501	0.248	2.496	1.391
SESN3	1.282	2.432	0.032	0.317	3.802	2.520	26.877	9.909	9.482	12.114	8.299	16.798	8.490	20.840	1.326	8.074	7.937
IL33	1.260	2.395	0.017	0.264	2.113	0.852	5.350	1.526	1.306	6.611	3.794	2.447	5.904	2.808	0.226	1.737	0.755
TIMP3	1.255	2.387	0.006	0.214	8.423	7.168	558.899	297.441	259.842	464.162	252.603	359.500	278.207	371.574	110.373	271.046	97.183
IGFBP6	1.235	2.354	0.000	0.100	7.924	6.688	292.217	193.677	201.235	216.266	242.159	323.098	213.722	283.505	91.174	143.807	81.166
PTGES	1.204	2.303	0.007	0.214	1.817	0.614	1.543	4.231	1.973	1.834	1.253	2.115	4.889	4.133	0.233	1.081	0.395
ADAM12	1.119	2.172	0.015	0.255	5.409	4.289	31.277	58.758	19.523	59.619	40.365	55.866	65.851	27.111	16.602	19.594	19.629
CCL26	1.104	2.149	0.011	0.236	1.895	0.791	2.223	2.349	1.133	3.683	1.311	4.368	3.773	4.730	0.485	0.494	1.335
TIMP1	1.100	2.144	0.038	0.334	9.537	8.437	1074.618	352.076	1152.687	887.673	569.813	847.838	610.720	803.327	184.346	699.666	319.492
PDGFRA	0.871	1.829	0.013	0.246	6.118	5.247	83.585	50.376	47.015	77.202	68.386	85.554	104.559	51.490	37.986	49.338	26.932
TP53I3	0.844	1.795	0.013	0.243	3.911	3.067	17.885	11.572	10.135	12.165	12.759	21.810	11.104	18.838	4.850	9.483	8.606
HIF1A	0.776	1.712	0.021	0.278	7.624	6.848	186.966	225.529	135.590	257.141	239.250	161.081	239.897	161.659	179.943	79.196	104.427
TGFBR2	0.770	1.705	0.015	0.253	5.159	4.389	42.673	30.826	34.259	39.798	32.142	38.381	42.724	22.225	15.025	31.681	16.562
MAPK14	0.666	1.587	0.025	0.292	3.062	2.395	7.092	9.606	7.843	8.756	4.369	7.187	8.156	6.924	4.068	2.678	6.813
UCHL1	0.631	1.549	0.023	0.284	6.920	6.289	147.835	136.694	92.255	117.488	119.429	117.334	117.470	119.786	123.413	71.099	52.257
TXN	0.622	1.539	0.049	0.362	7.135	6.514	159.197	164.517	187.486	196.760	162.479	103.317	99.874	88.843	79.315	102.664	90.655

TGFB1	0.620	1.537	0.006	0.214	5.125	4.505	32.542	38.831	45.649	33.412	25.508	32.046	36.915	29.928	16.907	25.570	23.629
PLAUR	0.615	1.531	0.034	0.324	3.267	2.652	12.653	8.467	9.159	6.640	6.413	10.116	9.696	7.343	3.474	4.956	8.323
ADAM10	0.467	1.383	0.040	0.341	4.478	4.011	20.913	21.356	21.079	18.590	27.155	20.322	22.297	19.583	10.482	23.003	14.212
ADAM17	0.431	1.349	0.027	0.302	3.691	3.260	9.823	10.894	14.301	12.129	11.219	12.596	11.679	13.236	6.532	12.260	7.802
MMP14	0.359	1.282	0.029	0.308	6.923	6.564	137.678	132.865	137.250	100.680	106.469	96.395	126.708	133.928	86.172	88.877	107.192
CRYAB	-1.617	0.326	0.014	0.251	3.770	5.387	11.937	16.846	28.245	10.389	13.497	7.137	7.649	14.279	84.860	15.106	52.020
PRKAG2	-1.505	0.352	0.003	0.160	2.384	3.888	3.206	3.957	6.873	3.705	6.768	3.679	2.493	4.610	24.343	6.919	15.180
CREB5	-0.908	0.533	0.001	0.139	0.657	1.565	0.536	0.526	0.945	0.517	0.259	0.634	0.484	0.812	1.107	1.862	3.295
ADCY9	-0.846	0.557	0.000	0.105	2.372	3.218	3.767	4.452	3.492	4.010	5.434	4.067	4.572	3.864	11.085	6.133	8.345
PRKAA2	-0.776	0.584	0.011	0.234	0.875	1.650	0.293	0.480	0.879	0.491	1.203	0.989	1.136	1.546	2.528	1.251	2.893
CD9	-0.752	0.594	0.045	0.356	3.787	4.540	19.113	10.869	20.614	8.799	15.793	8.010	9.725	15.097	32.260	19.753	17.226
PIK3R1	-0.670	0.628	0.007	0.214	1.688	2.358	1.544	1.819	2.187	2.140	3.025	1.451	2.710	3.412	3.538	4.199	4.708

Table S5. The differentially expressed genes between lean hASCs and obese hASCs which involved in the extracellular matrix remodeling

ID	Gene_Name	log2FC	Fold_Change	p_value	q_value	Obese_FPKM	Control_FPKM	obese 1	obese 2	obese 3	obese 4	obese 5	obese 6	obese 7	obese 8	lean 1	lean 2	lean 3
**BP**																		
GO:0030198	ADAM12	1.119	2.172	0.015	0.255	5.409	4.289	31.277	58.758	19.523	59.619	40.365	55.866	65.851	27.111	16.602	19.594	19.629
	COL16A1	0.752	1.684	0.010	0.228	6.046	5.295	66.772	67.229	56.956	62.824	58.134	50.196	88.347	77.714	39.697	55.269	25.409
	CYP1B1	1.259	2.392	0.007	0.214	6.341	5.082	48.595	78.912	158.097	84.679	68.754	69.181	109.186	62.856	32.463	49.757	21.884
	ENG	0.758	1.691	0.025	0.292	6.771	6.013	85.759	99.871	95.071	117.557	96.116	110.863	129.249	142.506	53.103	115.316	41.796
	FBLN1	1.590	3.010	0.023	0.286	3.408	1.818	8.623	10.641	23.505	10.134	4.652	5.716	7.576	15.167	1.171	9.035	1.011
	FBLN2	1.608	3.048	0.007	0.214	7.433	5.825	168.770	106.374	300.557	179.154	129.582	169.843	197.337	180.521	27.711	144.261	42.699
	FBLN5	0.979	1.971	0.047	0.359	4.768	3.789	35.615	17.324	48.737	18.500	29.248	15.135	19.349	46.050	10.692	20.815	9.358
	FLRT2	1.183	2.271	0.035	0.324	2.138	0.955	2.891	1.708	1.466	4.278	4.931	4.950	4.893	3.928	0.019	3.690	0.523
	LAMC2	1.332	2.517	0.018	0.269	2.985	1.653	9.442	2.724	5.316	7.529	11.869	3.789	11.923	8.241	0.730	3.885	2.680
	MMP16	1.540	2.908	0.015	0.255	2.824	1.284	8.000	11.254	8.606	8.959	1.261	4.687	7.041	4.791	0.449	3.068	1.448
	PDGFRA	0.871	1.829	0.013	0.246	6.118	5.247	83.585	50.376	47.015	77.202	68.386	85.554	104.559	51.490	37.986	49.338	26.932
	PTX3	0.654	1.573	0.038	0.334	9.744	9.091	842.321	824.780	814.488	714.202	912.640	1008.985	950.495	820.675	338.542	994.041	478.923
	TGFB1	0.620	1.537	0.006	0.214	5.125	4.505	32.542	38.831	45.649	33.412	25.508	32.046	36.915	29.928	16.907	25.570	23.629
	TIMP1	1.100	2.144	0.038	0.334	9.537	8.437	1074.618	352.076	1152.687	887.673	569.813	847.838	610.720	803.327	184.346	699.666	319.492
	TNXB	1.894	3.716	0.008	0.220	5.399	3.505	48.785	29.123	47.960	59.639	65.679	22.072	33.508	41.454	6.941	34.929	4.129
GO:0085029	FBLN5	0.979	1.971	0.047	0.359	4.768	3.789	35.615	17.324	48.737	18.500	29.248	15.135	19.349	46.050	10.692	20.815	9.358
	TGFB1	0.620	1.537	0.006	0.214	5.125	4.505	32.542	38.831	45.649	33.412	25.508	32.046	36.915	29.928	16.907	25.570	23.629
	TNXB	1.894	3.716	0.008	0.220	5.399	3.505	48.785	29.123	47.960	59.639	65.679	22.072	33.508	41.454	6.941	34.929	4.129
GO:0022617	ADAMTS5	-1.775	0.292	0.005	0.201	1.433	3.208	0.302	0.526	6.532	1.452	1.352	2.066	2.542	2.011	5.889	8.378	11.220
	SCUBE3	-1.719	0.304	0.031	0.315	4.283	6.002	28.871	37.022	37.202	12.249	16.592	4.033	9.345	38.248	94.281	41.754	63.610
	TLL1	-0.945	0.519	0.006	0.213	0.030	0.975	0.000	0.000	0.064	0.023	0.000	0.000	0.086	0.000	1.687	0.017	1.779
GO:0048251	FBLN5	0.979	1.971	0.047	0.359	4.768	3.789	35.615	17.324	48.737	18.500	29.248	15.135	19.349	46.050	10.692	20.815	9.358
	TNXB	1.894	3.716	0.008	0.220	5.399	3.505	48.785	29.123	47.960	59.639	65.679	22.072	33.508	41.454	6.941	34.929	4.129
GO:0030199	CYP1B1	1.259	2.392	0.007	0.214	6.341	5.082	48.595	78.912	158.097	84.679	68.754	69.181	109.186	62.856	32.463	49.757	21.884
	TNXB	1.894	3.716	0.008	0.220	5.399	3.505	48.785	29.123	47.960	59.639	65.679	22.072	33.508	41.454	6.941	34.929	4.129
**MF**																		
GO:0001968	FBLN1	1.590	3.010	0.023	0.286	3.408	1.818	8.623	10.641	23.505	10.134	4.652	5.716	7.576	15.167	1.171	9.035	1.011
	FSTL3	1.136	2.197	0.023	0.287	4.838	3.702	23.763	26.693	40.728	18.695	22.403	23.864	30.581	42.206	24.118	4.837	14.041
	IGFBP3	2.499	5.655	0.024	0.289	8.370	5.871	186.949	197.847	405.252	371.571	385.995	442.859	296.844	496.046	5.621	168.253	177.850
	IGFBP6	1.235	2.354	0.000	0.100	7.924	6.688	292.217	193.677	201.235	216.266	242.159	323.098	213.722	283.505	91.174	143.807	81.166
GO:0050431	ACVRL1	0.693	1.616	0.022	0.282	2.005	1.312	3.980	2.340	3.772	2.348	2.490	2.611	3.173	3.825	1.426	2.886	0.625
	ENG	0.758	1.691	0.025	0.292	6.771	6.013	85.759	99.871	95.071	117.557	96.116	110.863	129.249	142.506	53.103	115.316	41.796
	TGFBR2	0.770	1.705	0.015	0.253	5.159	4.389	42.673	30.826	34.259	39.798	32.142	38.381	42.724	22.225	15.025	31.681	16.562
GO:0034713	ENG	0.758	1.691	0.025	0.292	6.771	6.013	85.759	99.871	95.071	117.557	96.116	110.863	129.249	142.506	53.103	115.316	41.796
	TGFB1	0.620	1.537	0.006	0.214	5.125	4.505	32.542	38.831	45.649	33.412	25.508	32.046	36.915	29.928	16.907	25.570	23.629
	TGFBR2	0.770	1.705	0.015	0.253	5.159	4.389	42.673	30.826	34.259	39.798	32.142	38.381	42.724	22.225	15.025	31.681	16.562

	COL16A1	0.752	1.684	0.010	0.228	6.046	5.295	66.772	67.229	56.956	62.824	58.134	50.196	88.347	77.714	39.697	55.269	25.409
	CTHRC1	0.873	1.831	0.046	0.357	3.005	2.132	7.858	6.267	3.488	14.706	5.038	11.585	8.117	4.482	3.222	2.619	4.512
	FBLN1	1.590	3.010	0.023	0.286	3.408	1.818	8.623	10.641	23.505	10.134	4.652	5.716	7.576	15.167	1.171	9.035	1.011
	FBLN2	1.608	3.048	0.007	0.214	7.433	5.825	168.770	106.374	300.557	179.154	129.582	169.843	197.337	180.521	27.711	144.261	42.699
GO:0005201	FBLN5	0.979	1.971	0.047	0.359	4.768	3.789	35.615	17.324	48.737	18.500	29.248	15.135	19.349	46.050	10.692	20.815	9.358
	LAMC2	1.332	2.517	0.018	0.269	2.985	1.653	9.442	2.724	5.316	7.529	11.869	3.789	11.923	8.241	0.730	3.885	2.680
	PRG4	0.737	1.667	0.010	0.229	0.857	0.120	1.371	0.413	1.184	1.420	0.525	0.248	0.582	1.175	0.071	0.197	0.000
	TFPI2	2.450	5.464	0.001	0.123	6.325	3.875	111.249	100.689	86.143	147.941	71.640	88.116	79.259	21.127	9.132	20.006	13.828
	TNXB	1.894	3.716	0.008	0.220	5.399	3.505	48.785	29.123	47.960	59.639	65.679	22.072	33.508	41.454	6.941	34.929	4.129
	BST2	1.753	3.370	0.013	0.243	5.825	4.072	21.122	195.225	52.688	50.106	61.450	64.686	53.631	38.860	11.154	27.025	12.966
	LXN	1.819	3.529	0.004	0.187	5.826	4.007	53.344	56.842	27.046	122.723	49.332	87.008	60.087	35.290	14.800	27.029	8.376
GO:0008191	TIMP1	1.100	2.144	0.038	0.334	9.537	8.437	1074.618	352.076	1152.687	887.673	569.813	847.838	610.720	803.327	184.346	699.666	319.492
	TIMP3	1.255	2.387	0.006	0.214	8.423	7.168	558.899	297.441	259.842	464.162	252.603	359.500	278.207	371.574	110.373	271.046	97.183
**CC**																		
	CDH2	1.357	2.561	0.030	0.310	7.336	5.980	177.264	154.421	159.676	109.109	90.680	223.635	197.376	231.513	20.839	142.198	79.402
	COL16A1	0.752	1.684	0.010	0.228	6.046	5.295	66.772	67.229	56.956	62.824	58.134	50.196	88.347	77.714	39.697	55.269	25.409
	CTHRC1	0.873	1.831	0.046	0.357	3.005	2.132	7.858	6.267	3.488	14.706	5.038	11.585	8.117	4.482	3.222	2.619	4.512
	FBLN1	1.590	3.010	0.023	0.286	3.408	1.818	8.623	10.641	23.505	10.134	4.652	5.716	7.576	15.167	1.171	9.035	1.011
	FBLN2	1.608	3.048	0.007	0.214	7.433	5.825	168.770	106.374	300.557	179.154	129.582	169.843	197.337	180.521	27.711	144.261	42.699
	FBLN5	0.979	1.971	0.047	0.359	4.768	3.789	35.615	17.324	48.737	18.500	29.248	15.135	19.349	46.050	10.692	20.815	9.358
GO:0062023	LAMC2	1.332	2.517	0.018	0.269	2.985	1.653	9.442	2.724	5.316	7.529	11.869	3.789	11.923	8.241	0.730	3.885	2.680
	PRG4	0.737	1.667	0.010	0.229	0.857	0.120	1.371	0.413	1.184	1.420	0.525	0.248	0.582	1.175	0.071	0.197	0.000
	SCARA3	0.799	1.740	0.046	0.357	3.121	2.322	11.717	8.497	6.288	6.209	5.481	5.079	7.786	13.952	4.114	2.161	6.738
	SERPINE2	1.895	3.720	0.009	0.223	8.584	6.689	455.147	308.395	508.143	261.947	261.722	304.506	537.716	575.752	33.839	301.187	103.312
	TGFB1	0.620	1.537	0.006	0.214	5.125	4.505	32.542	38.831	45.649	33.412	25.508	32.046	36.915	29.928	16.907	25.570	23.629
	TIMP1	1.100	2.144	0.038	0.334	9.537	8.437	1074.618	352.076	1152.687	887.673	569.813	847.838	610.720	803.327	184.346	699.666	319.492
	TIMP3	1.255	2.387	0.006	0.214	8.423	7.168	558.899	297.441	259.842	464.162	252.603	359.500	278.207	371.574	110.373	271.046	97.183
	TNXB	1.894	3.716	0.008	0.220	5.399	3.505	48.785	29.123	47.960	59.639	65.679	22.072	33.508	41.454	6.941	34.929	4.129
GO:0071953	FBLN1	1.590	3.010	0.023	0.286	3.408	1.818	8.623	10.641	23.505	10.134	4.652	5.716	7.576	15.167	1.171	9.035	1.011
	FBLN5	0.979	1.971	0.047	0.359	4.768	3.789	35.615	17.324	48.737	18.500	29.248	15.135	19.349	46.050	10.692	20.815	9.358
	CDH2	1.357	2.561	0.030	0.310	7.336	5.980	177.264	154.421	159.676	109.109	90.680	223.635	197.376	231.513	20.839	142.198	79.402
	COL16A1	0.752	1.684	0.010	0.228	6.046	5.295	66.772	67.229	56.956	62.824	58.134	50.196	88.347	77.714	39.697	55.269	25.409
	CTHRC1	0.873	1.831	0.046	0.357	3.005	2.132	7.858	6.267	3.488	14.706	5.038	11.585	8.117	4.482	3.222	2.619	4.512
	FBLN1	1.590	3.010	0.023	0.286	3.408	1.818	8.623	10.641	23.505	10.134	4.652	5.716	7.576	15.167	1.171	9.035	1.011
	FBLN2	1.608	3.048	0.007	0.214	7.433	5.825	168.770	106.374	300.557	179.154	129.582	169.843	197.337	180.521	27.711	144.261	42.699
	FBLN5	0.979	1.971	0.047	0.359	4.768	3.789	35.615	17.324	48.737	18.500	29.248	15.135	19.349	46.050	10.692	20.815	9.358
	FLRT2	1.183	2.271	0.035	0.324	2.138	0.955	2.891	1.708	1.466	4.278	4.931	4.950	4.893	3.928	0.019	3.690	0.523
	FLRT3	0.634	1.552	0.000	0.089	0.891	0.257	1.124	0.542	0.688	0.827	0.994	0.747	1.163	0.844	0.093	0.257	0.242
	LAMC2	1.332	2.517	0.018	0.269	2.985	1.653	9.442	2.724	5.316	7.529	11.869	3.789	11.923	8.241	0.730	3.885	2.680
GO:0031012	MMP16	1.540	2.908	0.015	0.255	2.824	1.284	8.000	11.254	8.606	8.959	1.261	4.687	7.041	4.791	0.449	3.068	1.448
	PRG4	0.737	1.667	0.010	0.229	0.857	0.120	1.371	0.413	1.184	1.420	0.525	0.248	0.582	1.175	0.071	0.197	0.000
	PTX3	0.654	1.573	0.038	0.334	9.744	9.091	842.321	824.780	814.488	714.202	912.640	1008.985	950.495	820.675	338.542	994.041	478.923
	SCARA3	0.799	1.740	0.046	0.357	3.121	2.322	11.717	8.497	6.288	6.209	5.481	5.079	7.786	13.952	4.114	2.161	6.738
	SERPINE2	1.895	3.720	0.009	0.223	8.584	6.689	455.147	308.395	508.143	261.947	261.722	304.506	537.716	575.752	33.839	301.187	103.312
	TFPI2	2.450	5.464	0.001	0.123	6.325	3.875	111.249	100.689	86.143	147.941	71.640	88.116	79.259	21.127	9.132	20.006	13.828
	TGFB1	0.620	1.537	0.006	0.214	5.125	4.505	32.542	38.831	45.649	33.412	25.508	32.046	36.915	29.928	16.907	25.570	23.629
	TIMP1	1.100	2.144	0.038	0.334	9.537	8.437	1074.618	352.076	1152.687	887.673	569.813	847.838	610.720	803.327	184.346	699.666	319.492

TIMP3	1.255	2.387	0.006	0.214	8.423	7.168	558.899	297.441	259.842	464.162	252.603	359.500	278.207	371.574	110.373	271.046	97.183
TNXB	1.894	3.716	0.008	0.220	5.399	3.505	48.785	29.123	47.960	59.639	65.679	22.072	33.508	41.454	6.941	34.929	4.129
